# Multi-Ferrocene-Based Ligands: From Design to Applications

**DOI:** 10.1021/acs.chemrev.4c00295

**Published:** 2025-03-17

**Authors:** Axel Straube, Liridona Useini, Evamarie Hey-Hawkins

**Affiliations:** † Faculty of Chemistry and Mineralogy, Institute of Inorganic Chemistry, Leipzig University, Johannisallee 29, 04103 Leipzig, Germany; ‡ Wiley-VCH, Boschstr. 12, 69451 Weinheim, Germany; § Evonik Industries AG, Goldschmidtstr. 100, 45127 Essen, Germany; ∥ Faculty of Chemistry and Mineralogy, Institute of Bioanalytical Chemistry, Leipzig University, Deutscher Platz 5, 04103 Leipzig, Germany; ⊥ Department of Chemistry, Babes-Bolyai University, 1, Kogalniceanu str., RO-400084 Cluj-Napoca, Romania

## Abstract

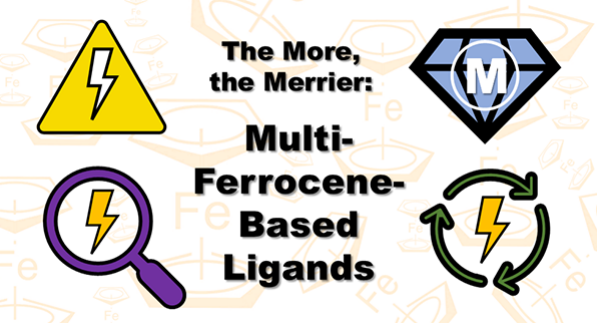

Despite the extensive literature on ferrocene chemistry, a comprehensive analysis of multiferrocene ligands is notably absent. Thus, this review presents an overview of multiferrocenyl-containing ligands, focusing on their synthesis, characterization, and applications in catalysis and sensing. These ligands offer unique properties, including redox activity and planar chirality, making them valuable in asymmetric catalysis and molecular electronics. The review covers the literature from the first synthesis of tris(ferrocenyl)phosphane in 1962 to current developments, including various ligand subsets, which contain at least two ferrocene units within their structure. Special attention is given to explaining the coordination chemistry, electrochemical behavior, and practical applications of these ligands. The aim of this undertaking is to fill gaps in knowledge and inspire further research by identifying areas for exploration. Notably, certain ligand families like TRAP (*trans*-spanning phosphane) ligands remain underexplored in terms of their electrochemical properties, highlighting opportunities for future investigation. Thus, this review provides a resource for researchers in the field, stimulating further advancements in multiferrocenyl ligand chemistry and its wide-ranging applications.

## 1 Introduction

**Figure 1:**
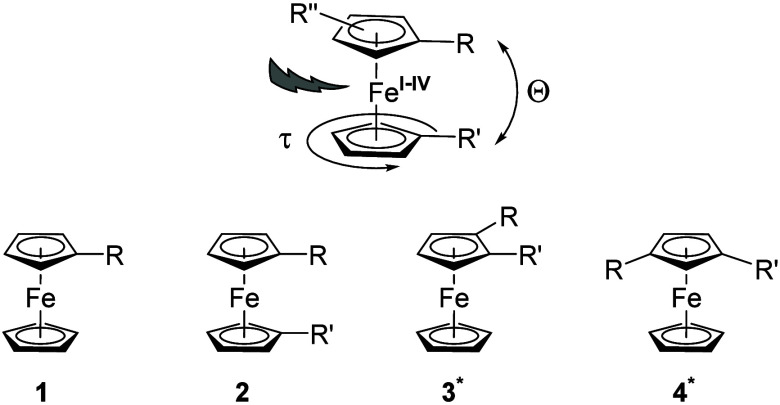
Important stereoelectronic parameters for substituted ferrocenes, possible oxidation states, and common substitution patterns **1** to **4** (the asterisk [*] denotes planar chirality if R ≠ R′). Adapted with permission from ref (3). Copyright 2012 John Wiley and Sons.

The importance of ferrocene (bis(η^5^-cyclopentadienyl)iron(II), **1**, R = H, Figure 1) for diverse fields of chemistry is undeniable. Therefore, over the past 70 years, a substantial body of literature, including numerous research articles, reviews and several books, has been amassed, underscoring ferrocene’s pivotal role in the field.^1^ Ligands incorporating multiple ferrocenyl units are of interest from different perspectives. One significant line of research concentrates on the assembly of chiral ligands, making use of ferrocene’s propensity for planar chirality.^2^

**Figure 2:**
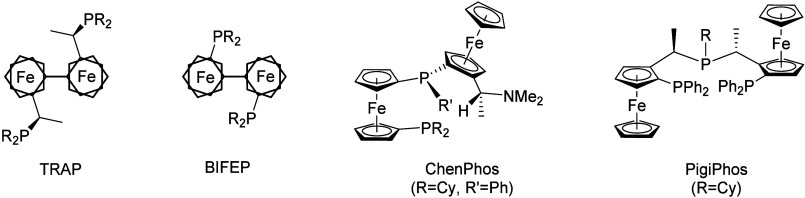
Representative selection of the most successful chiral bis(ferrocenyl)phosphane families and trivial/trademark names (R and R′ signify different residues, usually alkyl or aryl substituents; for the trademarked names, residues are specified).^4−9^

Often combined with other chiral elements, very attractive ligands for asymmetric catalysts such as the TRAP (*trans*-spanning phosphane, (*R*_C_*,R*_C_)-(*S*_Fc_*,S*_Fc_)-2,2″-bis[1-(phosphanyl)ethyl]-1,1″-biferrocene),^4^ BIFEP ((*R*_Fc_,*R*_Fc_)-2,2″-bis(phosphanyl)-1,1″-biferrocene),^5^ ChenPhos (1′-dicyclohexylphosphanyl-1-[(*S*_P_)-[(*S*_Fc_)-2-[(*R*_C_)-1-(dimethylamino)ethyl]ferrocenyl]phenylphosphanyl]ferrocene),^6^ and PigiPhos (bis{(*S*_Fc_)-1-[(*R*_c_)-2-(diphenylphosphanyl)ferrocenyl]ethyl}cyclohexylphosphane)^7−9^ ligand families have been developed (Figure 2, showing points of structural variability in the choice of residues R and R’).^10^ In the framework of this review, stereodescriptors for planar chirality of ferrocene compounds are labeled as *R*_Fc_/*S*_Fc_ to distinguish them from those of *C*-centered (*R*_c_/*S*_c_) and *P*-centered chirality (*R*_P_/*S*_P_) in contrast to the IUPAC Gold Book recommendation of using the subscript “P” as suffix for planar chirality.^11^

Another prominent feature of multiferrocenyl ligands stems from their ability to undergo redox processes, highlighting their distinctive electrochemical properties. Increasing the number of redox-active groups to *n* gives rise to *n* + 1 redox states which, given the right experimental conditions or deliberately chosen asymmetric ligand design, might even be selectively addressed. Investigations of electron transfer mechanisms in multiferrocenyl compounds have contributed to the understanding of the basic electron transfer processes and have enhanced the knowledge of charge-delocalized and mixed-valence systems.^12,13^ With respect but not limited to catalysts, such multistate redox-switchable systems provide intriguing starting points for fine-tuning and tempospatially controlling catalytic activity as, for example, demonstrated by our group^14^ as well as other teams.^15,16^ Moreover, the heightened redox response offered by multiferrocenyl compounds can be valuable in sensing applications, as recently reviewed by Hein, Beer, and Davis.^17^

In this review, we provide an account of various multiferrocenyl ligand classes including P, N, O, S, Se, and C donors. Since no previous review exists, we have covered the literature from the first preparation of tris(ferrocenyl)phosphane in 1962 until 2024. Summaries of ligand subsets, such as biferrocene-based ligands^10,18^ and ferrocene-appended porphyrins,^8^ were covered in reviews from 2009^10^ and 2013,^18^ respectively. Ferrocene-containing dendrimers have been very recently reviewed by Caminade and co-workers^19^ and by Astruc.^19,20^ Such systems will be briefly introduced in section 1.2. Selected examples of dendrimers containing donor atoms which have been used as ligands will also be presented in the respective sections, but the focus of this review remains on small molecules. Similarly, ferrocene-containing polymers have been the subject of review articles already.^21,22^ Some of such polymeric structures might be used to coordinate metals, as independently demonstrated by the groups of Mizuta^23^ and, more recently with the aim toward redox-responsive catalysis, by Barner-Kowollik and P. Roesky.^24^ Together with supramolecular assemblies that can contain large numbers of ferrocenyl or ferrocenylene groups,^25^ such systems, while intriguing, are not considered as part of this review to keep the focus on discrete molecular structures.

Details of the coordination chemistry as well as reported applications are presented alongside the electrochemical characterization. We have particularly focused on instances where the presence of more than one ferrocenyl group played a key role in the observed behavior. Assembling and commenting on existing studies, we aimed to address obvious gaps and missing links, intending to stimulate further research. Throughout the review, commentary on unavailable data on, for example, electrochemical characteristics, is not meant to downplay individual findings and achievements. Taking catalysis research as an example, knowing about the electrochemistry of a system is often not immediately relevant as long as the catalyst performs the intended reaction well and is stable under the given conditions. New and often more complex and difficult-to-prepare ligands must also compete with (industrially) established and simple ligands. Accordingly, many one-off forays into new structural motifs still lie dormant in the literature. While we tried our best to cover and introduce the most pertinent ligand classes and examples, the heterogeneous nature of the literature and missing naming conventions–or the missed opportunity to investigate and highlight the effect of the presence of two or more ferrocene units–will have necessarily meant that this account itself will have gaps and missing pieces.

To provide the reader with the necessary background information, a short overview of ferrocene and multiferrocene chemistry will set the stage for ligand classes categorized along their (most relevant) donor atoms.

### 1.1 Fundamentals of Ferrocene

Not many individual molecules can claim to have spawned an entire field of research, but ferrocene (**1**, R = H, Figure 1) is surely among them. Its serendipitous discovery in 1951, independently by Kealy and Pauson^26^ as well as by Miller and co-workers,^27^ resulted in enormous immediate interest of the chemical public.^28^ The iconic “sandwich” structure, the culinary analogy coined in 1953 by Orgel and Dunitz who also formulated a first description of the structure in terms of molecular orbital theory,^29^ was elucidated in simultaneous efforts by Wilkinson,^30^ Woodward (who also popularized the name “ferrocene” and was the first to notice the new compound’s propensity to undergo electrophilic aromatic substitution reactions),^31^ and Fischer^32^ and their respective co-workers. Honoring their contributions, Fischer and Wilkinson were awarded the Nobel Prize in Chemistry in 1973.^33^ Today, more than 70 years after its discovery, the research on ferrocene and its countless derivatives would fill entire libraries, and several books have tackled the gargantuan task to summarize the vast body of literature.^34−39^

Thus, when Astruc in his 2017 essay rhetorically asked why ferrocene was “so exceptional”,^40^ the answers were at hand: ferrocene is readily available in large quantities at a reasonable cost, stable under ambient and more extreme conditions, and susceptible to numerous derivatization strategies. Ferrocene has thus quickly become an indispensable building block of modern-day chemistry in general and in ligand design in particular,^3,41,42^ owing to its favorable stereoelectronic parameters (**1**–**4**, Figure 1).^43^ Among them, the defined bulk, the potential to serve as a node for further branching, the substitution-dependent rotatability (or its absence) around the iron-centroid(η^5^-cyclopentadienyl) axes (signified by the angle τ, Figure 1), its flexibility through a variable tilt angle Θ between the cyclopentadienyl planes (Figure 1), and possible planar chirality resulting from 1,2- (**3**) or 1,3-substitution (**4**) of the cyclopentadienyl rings (Figure 1) stand out.

Due to the nonbonding nature of the highest occupied and metal-centered molecular orbital a′_1g_,^44^ ferrocene (and its derivatives, the substitution determines the exact redox potentials) can easily be oxidized to the 17-valence electron ferrocenium cation. The highly reversible nature of this oxidation combined with the solubility of ferrocene in a wide range of organic solvents has led the International Union of Pure and Applied Chemistry (IUPAC) to declare the ferrocene/ferrocenium couple (FcH/[FcH]^+^, Fc = 1-ferrocenyl) the standard to which redox potentials in organic solutions should be referenced.^45^ Corresponding tables detailing the exact redox potentials in different supporting electrolytes (SE) have been published.^46^ More recently, derivatives with iron in the less common iron(I)^47^ and iron(IV)^48^ states have been prepared and characterized, too.

**Scheme 1:**
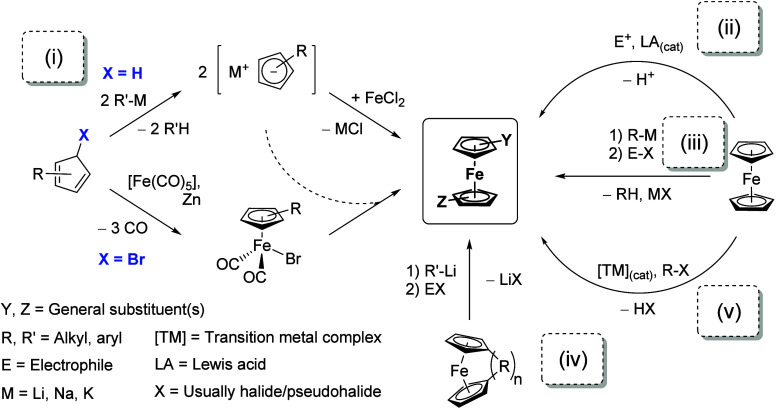
Schematic and Simplified Overview of Key Syntheses and Derivatization Strategies Towards (Multi-)substituted Ferrocenes (Y and Z at the Cyclopentadiene/Cyclopentadienide Ring Can Be R, R′, E, or a Different (Heteroatom) Substituent; Up to Five Substituents Per Ring Are Possible)

Methods to prepare and functionalize ferrocene derivatives are numerous; some key syntheses and derivatization strategies have been highlighted in Scheme 1. They can generally be grouped into the following categories (i) to (v) which, of course, can be combined and often build upon each other to construct structurally complex ferrocene derivatives:

(i) Reactions of substituted cyclopentadienes with metal salts or halide derivatives;
(ii) Electrophilic aromatic substitution reactions;
(iii) Sequential metalation-substitution protocols;
(iv) Ring-opening of (substituted) ferrocenophanes;
(v) Metal-catalyzed or -mediated derivatizations.

For this review, the preparation of selected ligand scaffolds of interest will be discussed where appropriate.

**Scheme 2:**
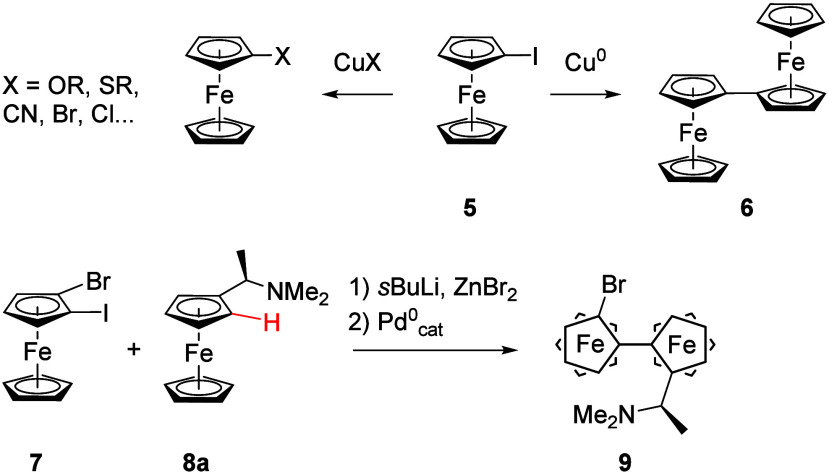
Selected Examples of Transition Metal-Mediated and -Catalyzed Transformations of Ferrocenes

The use of transition metals for functionalizing the cyclopentadienyl rings of ferrocene (strategy (v) in Scheme 1) has a long history, starting with the discovery of Rausch that iodoferrocene (**5**, Scheme 2) can undergo a copper(0)-mediated Ullmann-type coupling reaction to afford 1,1″-biferrocene (**6**, Scheme 2).^49^ In the same and in following work, copper(I) (pseudo)halides in combinations with pyridine or high-boiling solvents were furthermore found to effect (pseudo)halide substitution.^50,51^ This paved the way for the preparation of cyano- and, very importantly, aryloxy- and alkoxyferrocenes, the latter being the entry point for introducing various other oxo substituents.^49,52^ The copper salt methodology is still useful as demonstrated in 2018 by Dai and Yu in introducing a variety of sulfur-based nucleophiles in planar chiral fashion.^53^ Going from stoichiometric to catalytic use of copper salts, Plenio and co-workers prepared a number of ferrocenyl ethers from **5** and 1,1′-diiodoferrocene, respectively.^54^

In parallel, the stoichiometric use of copper(0) is also still employed for the synthesis of symmetric 1,1″-biferrocenes as demonstrated by the Weissensteiner group in 2009,^55^ mirroring the landmark syntheses of the BIFEP^5^ and TRAP^4^ ligand families (Figure 2), which relied on a nickel-mediated approach to connect the two preassembled ferrocenyl units.

Expanding on the idea and exploiting the different reactivities of the C_5_H_4_–I and C_5_H_4_–Br bonds in oxidative addition reactions with palladium, Weissensteiner, and co-workers were able to connect two differently substituted ferrocenes, planar chiral **7** and **8a** (the lithiated C–H site marked in red, Scheme 2),^18^ using the palladium-catalyzed Negishi protocol.^56^ Resulting 1,1″-biferrocene **9** was then converted to various bis-phosphanes.

Transition metal-catalyzed C–H functionalization of ferrocene often relies on (*ortho*-)directing groups for elegantly achieving enantio- and diastereoselectivity.^57,58^ A wide range of protocols is now available, leaving the choice between Suzuki-,^59,60^ Negishi-,^61^ and (dehydrogenative) Heck-like *C*,*C*-cross-coupling protocols.^62−64^ More unusual coupling reactions like cobalt(III)-catalyzed C–H amidations,^65^ rhodium(III)-catalyzed alkynylations employing hypervalent iodine reagents,^66^ or the introduction of oxa- and thiazoles using an oxidative protocol in air widen the synthetic scope.^59^

### 1.2 The More, the Merrier: Multiferrocene Systems

Against the backdrop of the extreme versatility and utility demonstrated by ferrocene in the previous section it should not come as a surprise that, since its discovery, scientists have tried to incorporate more than one ferrocene into individual molecules. The following section thus gives some selected background into key developments and concepts toward this goal.

**Figure 3:**
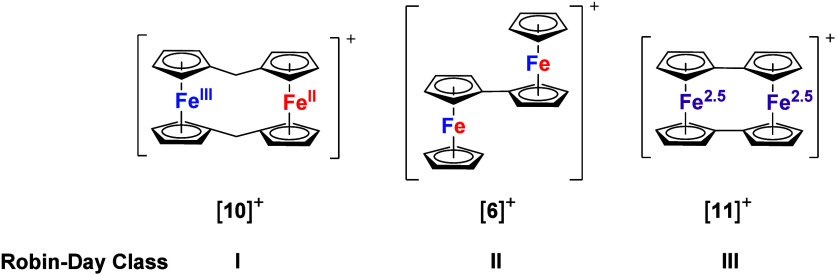
Monocations of [1.1]ferrocenophane (**10**), 1,1″-biferrocene (**6**), and [0.0]ferrocenophane (**11**) and their respective Robin–Day classifications (different colors indicate the loci of oxidation: separate for **[10]**^**+**^, partly exchanging for **[6]**^**+**^, and fully delocalized for **[11]**^**+**^).

Constructing multiferrocene systems is usually motivated by the redox properties of the individual ferrocene moieties which, when assembled, can add up to more than the mere sum of parts.^13,67^ The simplest case of what is commonly called electronic communication is already found in 1,1″-biferrocene (**6**, Scheme 2). When mono-oxidized, it first fulfils the basic requirement for a so-called mixed-valent system to consist of two, somehow linked, redox-active sites in different oxidation states.^68,69^ Mono-oxidized **[6]**^**+**^ (Figure 3) second shows a certain degree of delocalization, that is, medium-fast electron transfer (or electron hole transfer) between the formal Fe^II^ and Fe^III^ sites. The excitation of the electron transfer results in a characteristic absorption band in the near-IR (NIR) region of the UV/vis spectrum,^70^ usually called an intervalence charge transfer (IVCT) or, less commonly in this context, metal-to-metal charge transfer (MMCT). However, as evident from, for example, ^57^Fe Mössbauer spectroscopy, the two iron sites are not completely averaged in their oxidation states on that particular time scale.^71^ In contrast, the monocation of [0.0]ferrocenophane (**11**, also called biferrocenylene or bis(fulvalene)diiron(II), Figure 3) displays a ^57^Fe Mössbauer spectrum consistent with a fully averaged oxidation state of (formally) +2.5 per iron site as well as different NIR and UV/vis–spectroscopic features.^72^

When two methylene bridges connect the two ferrocenylene groups in [1.1]ferrocenophane (**10**; Figure 3), the resulting monocation, after some controversy, is now not considered to delocalize the electron hole, thus showing a clearly distinguishable Fe^II^ and Fe^III^ center, respectively.^73^ The extent of the electronic communication strongly depends on the nature of the spacer and the interatomic distances; in general, fully conjugated and electron-rich spacers allow for more efficient electronic communication, but through-space interactions have also been considered.^74,75^ A classification is given by the three classes proposed by Robin and Day which are usually assessed on the basis of a combination of UV/vis–NIR-spectroelectrochemistry and electrochemical measurements:^76^

(i) Class I compounds with fully localized valences and no measurable interaction between the individual redox sites (*e.g*., **[10]**^**+**^);
(ii) Class II compounds with measurable electron (hole) delocalization, a stabilized mixed-valent state, but the additional charge partly localized at one of the available sites (*e.g*., **[6]**^**+**^);
(iii) Class III compounds with complete electron (hole) delocalization and indistinguishable oxidation states on spectroscopic time scale (and in the solid state; *e.g*., **[11]**^**+**^).

**Figure 4:**
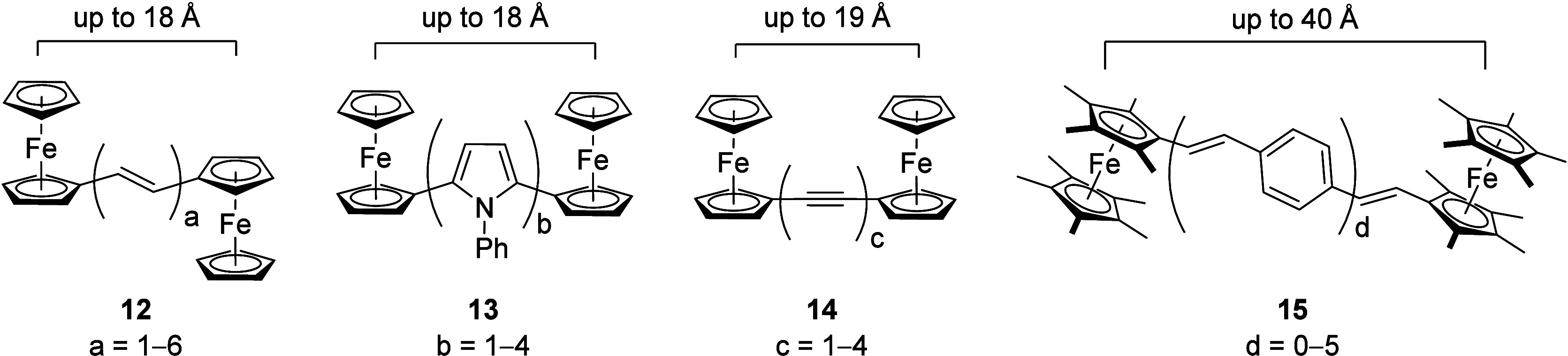
Ferrocenyl-terminated molecular wires with varying length of the fully conjugated organic spacers.

Next to the fundamental interest in studying such compounds and their electron-transfer properties, multiferrocene systems have also been extensively investigated with respect to their use in molecular electronics (*i.e.*, very roughly defined, constructing electronic devices on the molecular and nanoscale^77^).^78^ In this context of molecular electronics, linear and branched compounds containing multiple ferrocene units have been investigated as promising materials for molecular wires.^79−84^ Spangler and co-workers have shown that two ferrocenyl termini bridged by up to six repeating, all-*E* configured −HC=CH– units (**12**, Figure 4) showed a mixed-valent state upon mono-oxidation and efficient electronic communication over about 18 Å. While the relative arrangement of the ferrocenyl termini, *trans* as shown in Figure 4 or *cis*, has an influence, the study by Spangler found the effect of these differences to be outweighed by the experimental error of their determination.^85^ In contrast, the Lang group, using an (oligo)pyrrole linker (**13**), did not find electronic communication at the same metal–metal separation.^86^ Similarly, the (oligo)alkyne-bridged **14** did not show a mixed-valent mono-oxidized state.^87^ Nevertheless, the accomplishment of Bildstein, Launay, Schottenberger, and co-workers who assembled soluble molecular wires with two nonamethylferrocenyl termini separated by up to about 40 Å (**15**) remains remarkable, even though no electrochemical characterization was reported.^88^ Apart from wires, multiferrocenyl species also feature in molecular diodes^89^ and, more recently, a molecule containing four pentamethylcyclopentadienyl-iron(II/III) half-sandwich units has been proposed as a viable candidate for a quantum dot cellular automation.^90^

**Figure 5:**
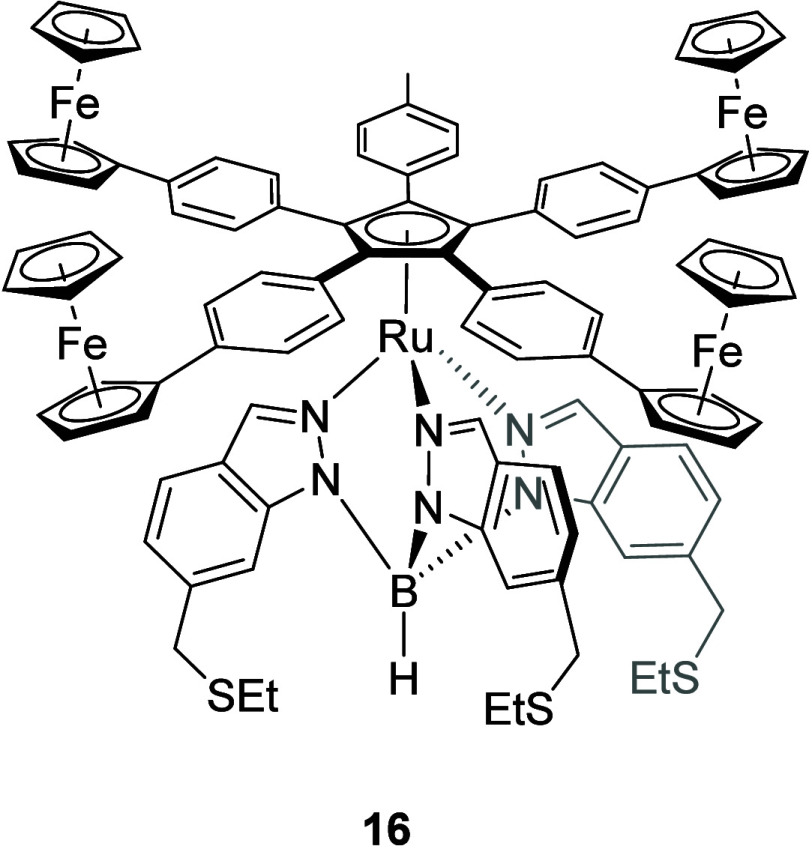
Molecular motor **16** with a tetrakis(ferrocenyl)-based rotor.^97^

Multiferrocenyl compounds also feature prominently in the realm of molecular machines.^91−93^ Next to the structural flexibility, particularly the “ball-bearing” character of ferrocene which is of great interest in constructing moving molecular assemblies responding to external stimuli,^94,95^ its special electronic configuration renders ferrocene an important building block in this area. In a prime example, Rapenne, Joachim, Hla, and co-workers have prepared a molecular motor **16** (Figure 5) capable of unidirectional motion. The key element of **16** is the tetrakis(ferrocenylphenyl)-substituted cyclopentadienide anion coordinated to a ruthenium(II) scorpionate complex. Exciting one of the arms through scanning tunneling microscopy (STM; inelastic tunneling of an electron through a molecule in the tunnel junction formed from the metal tip of the STM and the conducting surface deposits a quantum of energy into that molecule; the resulting excitation can then result in rotation if the rest of the molecule remains fixed),^96^ clockwise and anticlockwise rotation could be induced depending on which arm (ferrocenylphenyl or tolyl) was addressed.^97^

The burgeoning interest in the construction and application of multiferrocenyl systems, as demonstrated above, has resulted in a great number and structural variety of such compounds, making it difficult to establish a clear classification and ordering system. In their 2015 review, Santi and co-workers ordered compounds according to the number of ferrocenyl groups while restricting themselves to small molecules.^13^ In an earlier effort, Debroy and Roy sorted such compounds by the synthetic strategies used to access them from ferrocenes with unsaturated residues.^67^ Neither review included large supramolecular assemblies prepared via so-called coordination-driven self-assembly. These assemblies constitute a whole branch of multiferrocenyl systems with fascinating structures and potential applications in, for example, stimuli-responsive sensing of cations and anions.^98^ Selected examples of such self-assembled compounds will be discussed in the respective chapters dealing with multiferrocene ligands with specific donor atoms. For a general overview on ferrocene in the context of supramolecular systems, the reader is directed toward the 2015 review by Yang and team^98^ and to a recent review by Adhikari and co-workers.^25^

Furthermore, soon after the discovery of ferrocene, its incorporation into polymeric structures was attempted.^99^ Ferrocene can feature both in the backbone and in the side chains of polymers; in the latter case, ferrocene-containing monomers or postsynthetic modifications can be used. Accordingly, the concerned chemical space that has already been explored is large, and several review articles summarize advances.^7,9,10,21−23,99−103^ Potential applications of such systems are rich, and the resulting electrochromism of the reversible iron(II/III) redox couple can for example be exploited on the macroscopic scale in switchable smart windows.^104^

**Scheme 3:**
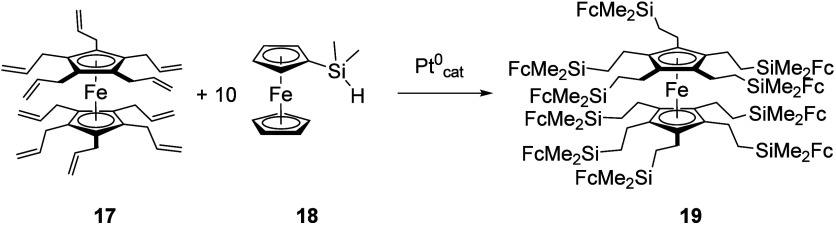
Ferrocenyl-Based Dendrimer with Central Ferrocenylene and Terminal Ferrocenyl Units

A special type of polymers, ferrocene-containing and/or -based dendrimers, also falls into this class of compounds.^105^ Defined as hyperbranched macromolecules of defined molecular architecture which are usually assembled in well-controlled and high-yielding synthetic steps, they offer exciting opportunities due to their modular nature.^106,107^ Ferrocene has been installed at the center,^108^ in the side chains,^109^ and in terminal positions of dendritic molecules.^110^ An early example is the hydrosilylation of decaallylferrocene (**17**, Scheme 3) with dimethylsilylferrocene (**18**) to yield decaferrocenylated dendrimer **19**.^111^ In similar approaches, oligosiloxane cores^112^ and carbosilane linkers^113^ have been used for highly ferrocenylated dendritic structures, highlighting the great structural diversity of such systems. These compounds, as in fact most ferrocenyl-containing dendrimers, show only one reversible oxidation for the ferrocenyl termini on the cyclic voltammetry time scale. This fact is attributed to fast tumbling of the dendrimers compared to the rate of heterogeneous electron transfer from the electrode to the molecule without significant through-space electronic communication between the individual termini.^114^ Nevertheless, the locally increased density of functional sites in close proximity as well as the high charge densities that can be created upon full oxidation make dendritic structures attractive as redox switches.

**Scheme 4:**
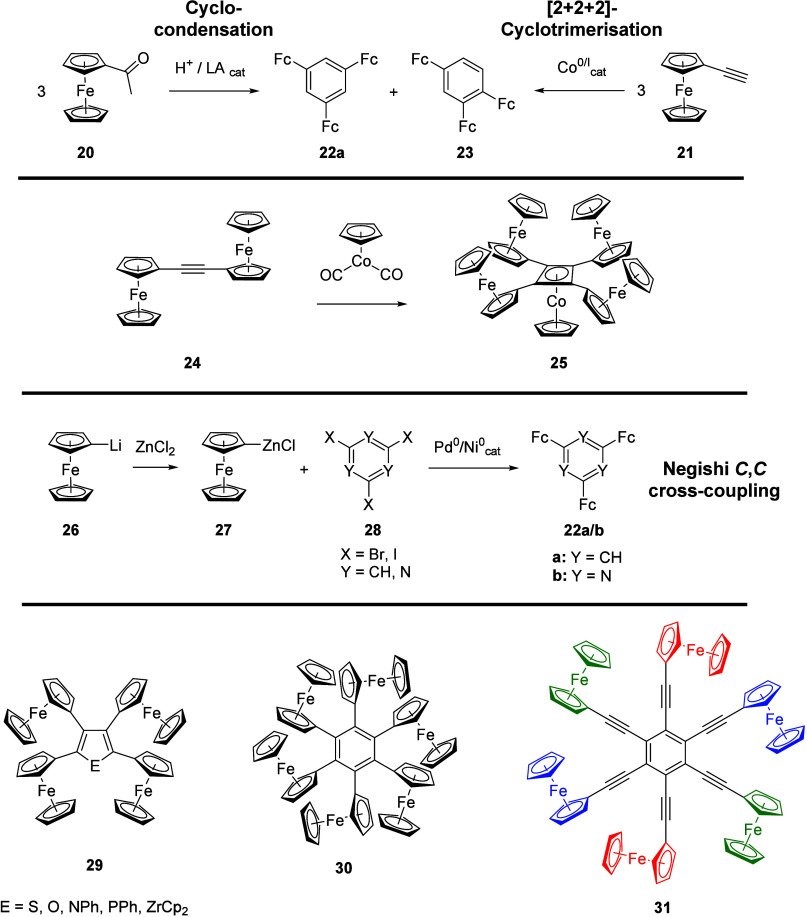
Synthetic Routes towards Ferrocenylated (Hetero)Aromatic Systems (LA = Lewis Acid) and Representative Per-Ferrocenylated Examples (Bottom: The Color-Coded Ferrocenyl Groups of **31** Are Oxidized at the Same Potentials, cf. Text)

Commonly (and usually in more straightforward and high-yielding syntheses), ferrocene-rich molecules are prepared around (hetero)aromatic cores.^115^ Of the available synthetic methods to prepare such systems, the cyclization routes shown in the top of Scheme 4 pose the inherent problem of regioselectivity as indicated in the competing formation of *C*_3*v*_-symmetric 1,3,5-tris(ferrocenyl)benzene (**22a**) vs its less symmetric 1,2,4-regioisomer **23**. To the best of our knowledge, the (Lewis) acid-catalyzed cyclocondensation of acetylferrocene (**20**) was first disclosed by Soukup and Schlögl^116^ as well as by the group of Aso in attempts to polymerize **20**.^117^ A more recent report from the McGlinchey group used SiCl_4_ in ethanol for the cyclization,^118^ and in all reports this synthetic methodology mainly yields **22a** but does not allow for great variation. Synthetic access to multiferrocenylated aromatics is also possible via cobalt-catalyzed [2+2+2]-cyclotrimerization of ferrocenylacetylene (**21**), again reported by Soukup and Schlögl using [Co_2_(CO)_8_].^119^ In this case, the formation of the 1,2,4-regioisomer **23** was favored and less **22a** was formed. Using [Co(η^5^-Cp)(CO)_2_], Rausch, Clearfield, and co-workers have synthesized tetrakis(ferrocenyl)cyclobutadiene from 1,2-bis(ferrocenyl)acetylene (**24**), which was obtained as a sandwich complex with a {CpCo} fragment (**25**, Scheme 4).^120^ Rausch et al. formulated the compound as a mixed cobalt(I) sandwich complex with a neutral tetrakis(ferrocenyl) η^4^-cyclobutadiene ligand. In light of the planarity and the virtually identical C–C distances of the C_4_ ring in the solid-state structure of a related 1,3-diphenyl-2,4-bis(ferrocenyl)-cyclobutadiene cobalt complex in the same paper, the cyclobutadiene might better be regarded as the corresponding Hückel-aromatic dianion, making the central atom formally cobalt(III).^120^ Despite its shortcomings, namely the formation of hard-to-separate mixtures, this protocol is still used, particularly in the context of [2+2]-cycloadditions of ferrocenyl-substituted butadienes.^121,122^

A much more widely embraced method was found in the transition metal-catalyzed *C*,*C*-cross-coupling reactions with emphasis on the protocols of Negishi (using zinc organyls, shown in Scheme 4), Stille (using organotin precursors), and Suzuki (using boronic acids and esters).^115^ The usefulness of these reactions was recognized by Iyoda in 1997 for the targeted and reliable synthesis of **22a** and has propelled the field forward considerably.^123^ The power of this protocol is exemplarily illustrated by the continued work of the Lang group, for example through attaching two to four ferrocenyl groups to all thinkable positions of five-membered heterocycles (only the tetrakis(ferrocenyl) derivatives **29** are shown in Scheme 4).^12,69^ Through this work, they have systematically elucidated the influence of both relative positioning and spacer influence on the degree of mixed valency, extending their studies to other spacer units such as fluorobenzenes^124^ and naphthalene.^125^ According to our survey, Lang and co-workers have also been the first to prepare 2,4,6-tris(ferrocenyl)-1,3,5-triazine (**22b**).^126^ Confirming results of Mayor and Weber^127^ as well as by Long and co-workers for related systems,^79^ the Lang group could thus show that the *meta* substitution pattern does usually lead to weaker electronic communication in the mixed-valent states of five- and six-membered heterocycles through detailed spectroelectrochemical investigations.^126,128^ In the case of **22b**, no charge delocalization was found for the mono- and dioxidized state (Robin–Day Class I). This stands in contrast to the weak Robin–Day Class II behavior observed for **22a**, suggesting the *s*-triazine to be a significantly worse charge transporter while also discounting the idea of through-space communication for this system, as the geometries of **22a** and **22b** are close to identical.^126^

Hexakis(ferrocenyl)benzene (**30**, Scheme 4), its preparation attempted unsuccessfully before by Schlögl and Soukup,^119^ provides a further demonstration of just how powerful the cross-coupling methodology has proven.^129^ In spite of only 4% isolated yield of **30** (with respect to the hexahalobenzene precursor), enough material had been obtained for electrochemical studies by Vollhardt and co-workers. Compound **30** was found to undergo three separated and reversible oxidations in a 1:2:3 ratio in a [PF_6_]^−^-based supporting electrolyte (SE), suggesting the successful formation of the hexacation. Inserting alkynyl spacers between the ferrocenyl termini and the central C_6_ core, Astruc and co-workers prepared the so-called “redox star” **31** (Scheme 4) through the Negishi protocol, using a ferrocenylalkyne-derived zinc organyl.^130^ Electrochemical and chemical oxidation of **31** was possible and reversible; under cyclic voltammetry conditions in an SE based on the [B(3,5-(CF_3_)_2_C_6_H_3_)_4_]^−^ anion, three separate two-electron oxidations could be distinguished which Astruc and his team ascribed to the pairwise oxidation of opposing termini, due to electrostatic frustration, as color-coded in Scheme 4.

## 2 Multiferrocene-Based Ligands

The continued interest in new cores and spectacular molecular architectures of multiferrocene systems notwithstanding, combining them with a second or even more metals can reinforce their inherent strengths of serving as redox-active multielectron reservoirs. Some of the multiferrocene assemblies presented in the previous sections already combined several ferrocenyl groups in one molecule, and this approach has been extended to a sizable variety of (potential) ligands. Somewhat surprisingly, no concise review of such systems has been presented so far, and given the great potential that such systems hold, their relative scarcity is unexpected. In the following, not necessarily comprehensive, overview, the (potential) ligands are sorted according to their donor atoms. To equip the readers of this review with a quick guide to which substances and substance classes have been characterized with what level of detail, the following overview figures come with a set of pictograms and a corresponding legend in each figure. Briefly, pictograms highlight available information on electrochemical characteristics, the availability of solid-state structures of the free (potential) ligand or its metal complexes, whether a compound has been tested in (redox-switchable) catalysis, and whether it has been applied as a molecular sensor. When a larger group of compounds is represented by a single set of icons, there will be some ambiguity because not every derivative will have every property available. However, we aim to showcase which compound classes are already explored in depth and might be taken forward for further exploration, and for which compounds less information is available. More detailed information is given in the text and, of course, in the original literature. This way, promising examples for next-generation applications as well as open gaps in the literature are hopefully more easily visible.

### 2.1 P Donor Ligands

Given the great success of phosphanes and other phosphorus-containing ligands in homogeneous catalysis,^131^ it does not come as a surprise that P ligands form the largest subset of multiferrocene-based ligands covered in this review. In the following, we have tried to group compounds based on their structures as far as possible.

**Figure 6:**
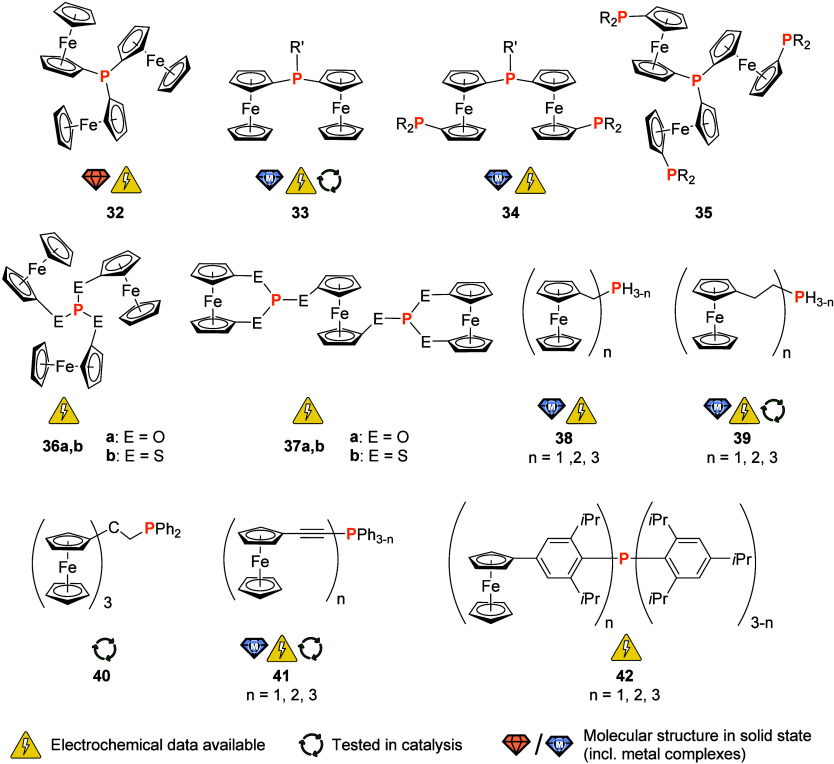
Achiral phosphorus ligands with two or more ferrocenyl or ferrocenylene groups in their backbone or as peripheral substituents; R, R′ = (cyclo-)alkyl, aryl). Compounds **38** (*n* = 1), **39** (*n* = 1), **41** (*n* = 1), and **42** (*n* = 1) are included as monoferrocenylated derivatives to underline that all three phosphanes have been prepared.

Employing a Friedel–Crafts-like protocol, Sollott and co-workers succeeded in preparing the archetypal tris(ferrocenyl)phosphane (**32**) (Figure 6) as early as 1962 by reacting ferrocene with 1.5 equiv of PCl_3_ in the presence of aluminum chloride. This likely constitutes the first-ever account of a P-bonded ferrocene.^132^ Following an initial investigation of ferrocenylphosphanes, their metal carbonyl complexes and electrochemical properties of selected systems as early as 1973,^133^ tris(ferrocenyl)phosphane (**32**) was found by Kotz and co-workers in 1975 to exhibit reversible redox behavior on the cyclic voltammetry time scale in both native and complexed form.^134^ That latter property was reinvestigated by Kirss and Geiger in 2005, using the very weakly coordinating perfluorinated tetrakis(pentafluorophenyl)borate anion (WCA = weakly coordinating anion) in the supporting electrolyte.^135^ Due to their high solubility, widely dispersed negative charge and resulting inertness toward strong electrophiles, this and other WCAs are very suitable for electrochemical studies in media of low polarity where smaller anions such as [PF_6_]^−^ and [BF_4_]^−^ are often found to form tight ion pairs and even engage in follow-up chemistry.^136−138^ Together with results from chemical oxidation experiments^139^ and in contrast to the earlier reports, Kirss, Geiger, and co-workers concluded that the oxidation of **32** and its analogues Fc*_n_*PPh_3–*n*_ (*n* = 1 (not shown), 2 (**33**, R′ = Ph; Figure 6) occurs from a largely iron-based highest occupied molecular orbital (HOMO) that, however, contains a significant contribution from phosphorus. These findings thus rationalize the often-observed, oxidation-induced, and *P*-centered reactivity of ferrocenylphosphanes, including the very commonly used 1,1′-bis(diphenylphosphanyl)ferrocene (dppf),^140^ tamable by engaging the lone pair of electrons in coordination of a Lewis acid.^141−143^ The coordination chemistry of tris(ferrocenyl)phosphane (**32**) is considerably less investigated^144,145^ than the ubiquitous all-phenyl analogue triphenylphosphane,^146,147^ even though it was found to be a better σ-donor.^134,145^ Mainly metal carbonyl complexes of group 6, iron ([Fe(CO)_4_L] with L = PFc_2_Ph, PFc_3_), and ruthenium ([Ru(CO)_4_(PFc_3_)], [Ru_3_(CO)_11_L] with L = PFc_2_Ph, PFc_3_, and [Ru_3_(CO)_10_L_2_] with L = PFc_2_Ph, PFc_3_) have been reported.^144,145^ Ogasawara et al. have also prepared a structurally characterized {AuCl} complex of **32** in their noteworthy investigation of the ferrocenyl group’s influence on steric and electronic properties of phosphanes such as **32** and **33**.^148^

Bis(ferrocenyl)(alkyl)phosphanes (**33**, R′ = Me, Et, *t*Bu) and the corresponding bis(ferrocenyl)(alkyl)phosphane oxides (Fc_2_P(O)R′) have been prepared in analogy to tris(ferrocenyl)phosphane (**32**) from the reaction of ferrocene with alkyldichlorophosphanes (R′PCl_2_, R′ = Me, Et, *t*Bu) in the presence of aluminum chloride. Cyclic voltammetry studies of **33** showed that the first oxidation is irreversible. In contrast, the corresponding oxides, more difficult to oxidize than **33**, showed two reversible oxidations separated by 140–160 mV.^149,150^ The Lang group has studied the σ-donor ability of mono*-*, bis*-*, and tris(ferrocenyl)phosphane ligands and observed that the σ-donor ability of the ligands PFc_*n*_*n*Bu_3–*n*_ (*n* = 0, 1, 2, and 3) in platinum acetylide complexes decreases as the number of ferrocenyl substituents increases.^151^ Other interesting bis(ferrocenyl)-based ligands (**33**, R′ = C_6_H_4_-2-NMe_2_ and C_6_H_4_-2-piperidyl) have also been reported by the Lang group and were shown to be highly effective in the palladium-catalyzed mono-α-arylation of acetone with various aryl halides.^152^ Derivatives of **33** that contain planar chiral 1,2-disubstituted ferrocenyl groups with further donor moieties (*e.g.*, NMe_2_ and OMe; see Figure 12 for further information) and aryl substituents R′ with electron-donating and -withdrawing substituents have also been investigated by Hayashi et al.^153^ The ligands, when tested in the Pd-catalyzed asymmetric hydrosilylation of 1,3-dienes^153^ and in the preparation of axially chiral allenylsilanes,^154^ performed with relatively high observed enantioselectivities.

The bis(ferrocenyl)phosphane motif has been taken up and expanded by the pioneering work of the Butler group. The so-called triphosfer phosphanes (**34**, Figure 6) (isopropyl and phenyl derivatives in different permutations have been reported) coordinate to ruthenium(II) with *mer* geometry,^155^ while *C*_3_-symmetric tetrakis-phosphane **35** (nicknamed manphos for its resemblance to the crest of the Isle of Man) did not yield characterizable complexes with a palladium(II) precursor compound.^156^

Tris(ferrocenyl)phosphite **36a** (E = O)^157^ and its thiophosphite analogue **36b** (E = S)^158^ have been prepared from FcOH or the disulfide FcS–SFc, respectively. While, unfortunately, no coordination studies have been reported for either **36a** or **36b**, the electrochemistry of **36b** and the corresponding chalcogen derivatives **36b(=E)** (E = O, Se) have been studied. While **36b** and **36b(=O)** were found to undergo reversible 3-fold oxidations in CH_3_CN, 0.1 M (TBA)[BF_4_] (TBA = *n*Bu_4_N), **36b(=S)** deviated from that clean-cut oxidative behavior,^159^ illustrating the often complex chemistry of ferrocenyl-containing P ligands. In this context, a very recent study on the electrochemistry of the full set of tris(ferrocenyl)pnictogens Fc_3_Pn (Pn = P, As, Sb, Bi) by Hupf, Beckmann, and co-workers is worth mentioning.^160^ In an in-depth investigation, the team compared various supporting electrolytes with different anions. Non-negligible through-space electronic interactions between the ferrocenyl groups in the respective monocations were found to be the most plausible reason for intervalence charge transfer observed by spectroelectrochemistry. In a variation on the motif of **36a,b**, Herberhold and team also reported on the related bis(thio)phosphite **37a,b**.^157,161,162^ While no complexation studies seem to have been undertaken, the bis-phosphite **37** has been investigated using cyclic voltammetry (CH_2_Cl_2_, 0.2 M (TBA)[ClO_4_], 100 mV/s) and was found to be reversibly oxidizable in two separate steps. With additional insight from differential pulse voltammetry, the authors assigned the first oxidation to the central ferrocenylene core.^161^

Primary to tertiary ferrocenylmethyl- (**38**)^163−166^ and ferrocenylethylphosphanes (**39**)^167,168^ (*n* = 1, 2, 3; Figure 6) have been prepared and their coordination to tantalum(V) ([Cp^R^TaCl_4_{PH(CH_2_Fc)_2_}] (Cp^R^ = C_5_Me_5_ or C_5_MeH_4_)),^169^ ruthenium(II) ([(*p*-cymene)RuClL_2_][PF_6_] (*p*-cymene = *p*-*^i^*PrC_6_H_4_Me, L = PH_2_CH_2_Fc and PH(CH_2_Fc)_2_), and *trans*-[RuCl_2_L_4_] (L = PH_2_CH_2_Fc, and PH(CH_2_Fc)_2_),^170^ and group 6 metal carbonyls ([MI(CO)_2_L_4_]I, M = W, Mo, L = PH_2_CH_2_Fc;^171^ [MI_2_(CO)_3–*n*_(PH_2_R)_2+*n*_], M = Mo, W, R = FcCH_2_, *n* = 0, 1;^172^ [M(CO)_5_L], M = Cr, Mo, and W, L = PH_2_(CH_2_CH_2_Fc), PH(CH_2_CH_2_Fc)_2_, and P(CH_2_CH_2_Fc)_3_^171−173^ has been studied. A tris(ferrocenyl)methyl group has also been installed in the backbone of a diphenylphosphane;^174^ Butenschön and Garabatos-Perera have successfully employed bulky **40** (Figure 6) in a Pd-catalyzed Suzuki–Miyaura coupling reaction. Compound **40** has been prepared from the corresponding carbinol which also serves as interesting entry point for follow-up chemistry.^175^

In preparing a family of (ferrocenylethynyl)phosphanes **41** (Figure 6), a ligand system with tunable steric bulk was introduced.^176^ All three phosphanes (*n* = 1, 2, 3) were used to coordinate a {PdCl_2_} fragment in L_2_PdCl_2_. Coordination of a {PtCl_2_} fragment for the bis- and tris(ferrocenylethynyl)phosphanes and the oxide for **41** (*n* = 3) were later reported by Lang and team.^177^ In CH_2_Cl_2_ (0.1 mM, (TBA)[PF_6_], 50 mV/s), **41** and their palladium(II) complexes show reversible, single-wave redox events solely ascribed to oxidation of the iron(II) centers. Because of the observed nonlinear shift in the redox potentials for the complexes, Baumgartner and co-workers proposed a *trans* geometry for [Pd(**41**)_2_Cl_2_] in the case of *n* = 2, 3. The solid-state structures of free **41** (*n* = 3), its selenide and [*cis*-Pd(**41**(*n* = 3))_2_Cl_2_] were later reported by the group of Lang.^178^ The full set of palladium(II) complexes for all three derivatives of **41** was also tested as precatalysts in Suzuki–Miyaura and Heck–Mizoroki cross-coupling reactions. The bis-ferrocenyl derivative was found to outperform the tris-ferrocenyl congener, especially in the Heck reaction, where the PdCl_2_ complex was found inactive. Compound **41** (*n* = 3) was also used to coordinate to {Fe_3_E_2_(CO)_*n*_} (E = Se, Te, *n* = 6–8) clusters in 1:1 and 1:2 cluster:ligand ratios (*n* being dependent on the coordination mode).^179^ X-ray crystallography was used to determine the structure of representative complexes, showing the feasibility of a *cis* arrangement of two such ligands in the octahedral coordination sphere of the iron center. Cyclic voltammetry (CH_3_CN, (TBA)[ClO_4_], scanning speed and SE concentration not reported) confirmed the fully reversible multielectron oxidation of the coordinated ligand.

Further increasing the steric bulk, Yoshifuji and co-workers prepared and investigated “crowded” triaryl phosphanes **42** with [4-ferrocenyl-2,6-bis(isopropyl)]phenyl and 2,4,6-tris(isopropyl)phenyl substituents (Figure 6).^180^ The lithiation–metal transfer–substitution reaction cascades with zinc and copper reagents furnished these very bulky phosphanes, of which the tris-ferrocenyl derivative **42** (*n* = 3) was characterized crystallographically, showing similar geometric properties as trimesitylphosphane. While no metal complexes of these appealing P donors have been reported, their electrochemical behavior stands out: cyclic voltammetry in 0.1 M (TBA)[ClO_4_], CH_2_Cl_2_ as supporting electrolyte (30 mV/s), in combination with square-wave voltammetry, revealed reversible oxidations at both the ferrocenyl and the phosphorus centers. EPR confirmed the formation of P-centered, unstable radicals. Although isolation of the radical species failed, **42** (*n* = 3) can still be considered a redox switch with five potential redox states.

**Figure 7:**
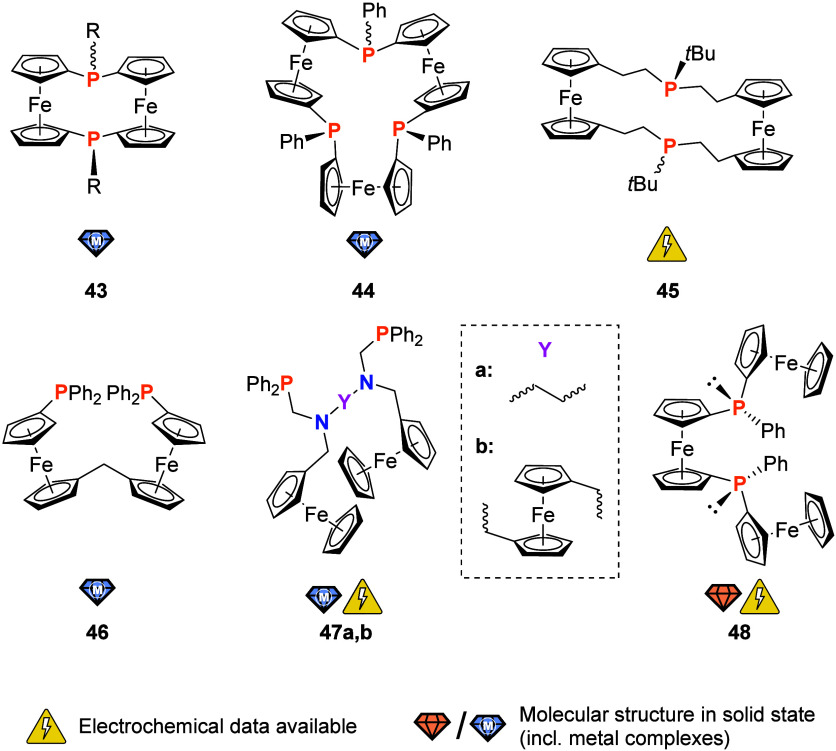
Phosphanes with ferrocenylene-based backbones (wavy bonds indicate the existence of both diastereomers; R = (cyclo)alkyl, aryl).

The combination of ferrocenophanes and phosphorus also proved fruitful in this context as demonstrated through diphospha[1.1]ferrocenophane **43**(181) and the macrocyclic triphospha[1.1.1]ferrocenophane **44** (Figure 7).^182^ In both cases, two diastereomers depending on the relative orientation of the two (**43**) or three (**44**) phenyl groups could be isolated from the photolysis of phospha[1]ferrocenophane. Naturally, the *syn* (**43**) and the all-*syn* (**44**) isomers (all lone pairs of electrons on phosphorus oriented toward the same direction) are intriguing chelate ligands. Indeed, a dichloridocobalt(II) moiety was bound by *syn*-**43**. In contrast, both diastereomers (all-*syn* and *syn*,*syn*,*anti*) of **44** were found to bind a silver(I) cation in the same *C*_3*v*_ symmetric, all-*syn* coordination geometry. Apparently, the energy gain upon coordination outweighs the energetic cost of the pyramidal inversion at phosphorus.^181,182^ The (−)-menthyl derivative of **43** had earlier been reported by Brunner and co-workers.^183^ Its preparation from reaction of 1,1′-dilithioferrocene with (−)-dichloromenthylphosphine resulted in the formation of other, larger species which made separation by medium-pressure liquid chromatography necessary. Presumably due to an isolated yield of only 2%, the team did not report any coordination studies, but already observed the *syn* configuration of the (−)-menthyl derivative of **43** by X-ray crystallography.^183^ Further derivatives of **43** with different substituents R, including a tetraphosphamacrocycle combining two [1.1]ferrocenophanes,^184^ have been prepared and studied for their coordination behavior (including coordination at Pd, Ni, Mn, Pt, Co, and Ag) by Mizuta and co-workers.^185,186^ While these are very interesting systems, their electrochemistry and potential applications remain yet to be explored. A third ferrocenophane **45** was prepared by Herber, Butenschön, and co-workers from the reaction of a ferrocenylene-bis-phosphane and a 1,1′-bis(bromoalkyl)ferrocene.^187^ Technically a bis-trialkylphosphane, the [5.5]ferrocenophane, obtained as a mixture of the *syn* and *anti* diastereomers, is moderately air stable. Cyclic voltammetry (CH_2_Cl_2_, 0.1 M (TBA)[PF_6_], 50 mV/s) indicates simultaneous and reversible oxidation of both ferrocenylene moieties. When **45** was treated with one equivalent of Ag[SbF_6_], a green material was obtained; using five equivalents of Ag[SbF_6_], a blue product was isolated. Using an array of Mössbauer spectroscopic experiments, the authors confidently assigned the product of the 1:1 reaction to be the monooxidized iron(II,III) species, whereas the 1:5 oxidation reaction product was assigned to be the dication. Despite the attractive stereo- and electrochemical features, **45** appears to not yet have been tested as an actual ligand.

Flexible coordination behavior was observed for bis-phosphane **46** (Figure 7) by Štěpnička and co-workers, which was conveniently prepared from selective monolithiation of 1-bromo-1′-diphenylphosphanylferrocene followed by reaction with diethyl carbonate and a subsequent reduction of the central C=O group.^188^ Compound **46** can act as a bidentate ligand as shown for palladium(II). Alternatively, the two phosphanyl groups of **46** can each bind one metal complex in a monodentate fashion as demonstrated for the chloridogold(I) fragment. Using the halide-free gold(I) precursor complex [Au(tht)_2_][SbF_6_] (tht = tetrahydrothiophene), a tetranuclear (with respect to gold(I)) macrocycle with 4-fold symmetry was formed. In a similar manner, tris(ferrocenyl)-bis-phosphane **47b** was used to bind gold(I) and ruthenium(II) complex fragments in a κ^1^*P*,κ^1^*P*′ coordination mode.^189^ In an earlier report, Smith and co-workers had already described a related structure **47a** with an ethylene C_2_H_4_ spacer linking the two amines instead of the ferrocenylene centerpiece.^190^ This somewhat simpler system had been successfully used as a bis-monodentate ligand for dinuclear ruthenium and gold complex fragments as well as chelating ligand for *cis* coordination of platinum(II), palladium(II), and molybdenum(0) centers. Metallamacrocycles of a 2:2 L:M stoichiometry with palladium(II) and rhodium(I) centers were additionally prepared. The free phosphane and many of its metal complexes were characterized crystallographically. In cyclic voltammetry (CH_2_Cl_2_, 0.1 M (TBA)[BF_4_], 50 mV/s), all compounds were found to be oxidized in one single step. In contrast, **47b** showed two separate oxidation events due to the presence of the central ferrocenylene spacer.^189^

The dppf derivative **48**, enantioselectively prepared as part of the continued expansion of the BIFEP family,^191^ seems to have remained a curiosity despite its promising performance in the Pd-catalyzed Tsuji–Trost allylation; the solid-state structure of the racemic bis-phosphane was reported in 2007.^192^ Much like for the BIFEP ligands, the electrochemical behavior of these intriguing and catalytically successful systems has not yet been reported.

**Figure 8:**
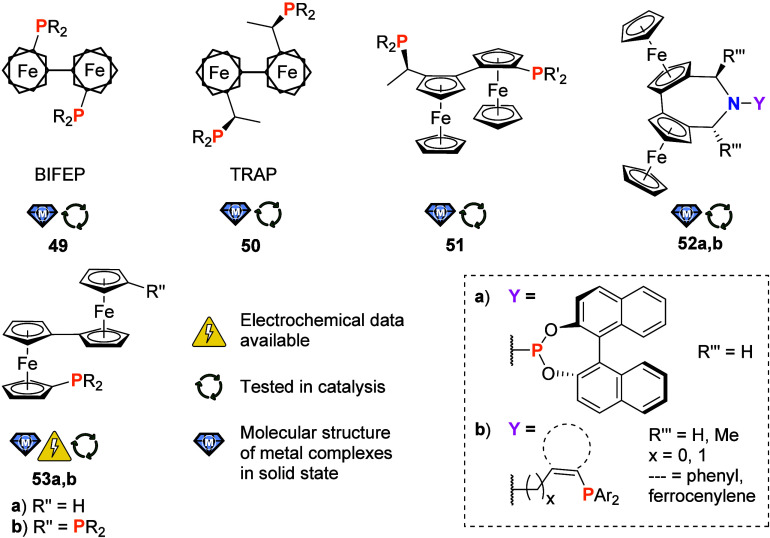
Biferrocene-based phosphane ligand families (R, R′ = (cyclo-)alkyl, aryl; Ar = substituted aryl).

1,1″-Biferrocenes (see **6**, Scheme 2) have also found their way into ligand design, as already mentioned for the BIFEP (**49**)^5^ and TRAP (**50**)^4^ ligand families (Figure 2 and Figure 8) which proved to be very valuable for asymmetric palladium-catalyzed *C*,*C*-cross-coupling reactions.^193^ Merging both ligand families has been achieved by Weissensteiner and team (**51**) in a rather elaborate synthesis.^18,194^ Crystal structures of several members of the **51** family and some of their Pd and Ru complexes have been reported,^18^ and tests for the performance of **51** in Ru-, Rh-, and Ir-catalyzed asymmetric hydrogenations have shown promising, albeit not always excellent, enantioselectivities.^195^ It is, at this point, worth pointing out that despite this big success of these ligand families for asymmetric catalysis, their electrochemistry remains woefully underexplored. The same holds, unfortunately, true for the even more complex ligand family **52** (Figure 8). By combination of the planar chirality of a biferrocene-based dihydroazepine and the axially chiral BINOL-derived phosphoramidite moiety in **52a**, van der Eycken et al. designed an intricate set of ligands (two diastereomers with differing axial chirality and a simple biphenyl derivative were prepared).^196^ For its use in asymmetric Rh-catalyzed hydrogenation reactions, only moderate enantioselectivities were observed. A whole suite of related dihydroazepine-based phosphanes **52b** (Figure 8) with variations of dihydroazepine substitution, alkyl spacer length *x*, spacer group (phenylene vs ferrocenylene) and phosphane substitution had earlier been reported by Weissensteiner, Widhalm, and team.^197^ A palladium(II) complex of a simpler derivative (nonsubstituted dihydroazepine, phenylene linker and PPh_2_ group) was analyzed crystallographically. The entire ligand set was also tested for its suitability for Pd-catalyzed allylic alkylation and amination reactions, albeit with limited success.

In contrast to the richly explored chemistry of BIFEP, TRAP, and their analogues, 1′,1′′′-disubstituted biferrocenes have been rarely studied for their use in ligand design. Mochida and co-workers have prepared complexes of **53b** (Figure 8) with ruthenium(II), iridium(I), and rhodium(I),^198^ and the Lang group has used biferrocenylated monophosphanes **53a** as ligands for a series of *trans*-coordinated palladium(II) complexes.^199^ The free phosphanes, as expected, show complex and nonreversible redox behavior in cyclic voltammetry irrespective of the chosen supporting electrolyte. However, the palladium(II) complexes *trans*-[Pd(**53a**)_2_Cl_2_], which were also employed in *C*,*C*-cross-coupling reactions, were found to display up to three reversible oxidations in supporting electrolytes with very weakly coordinating anions (CH_2_Cl_2_, 0.1 M (TBA)[B(C_6_F_5_)_4_], 100 mV/s).^199^

**Figure 9:**
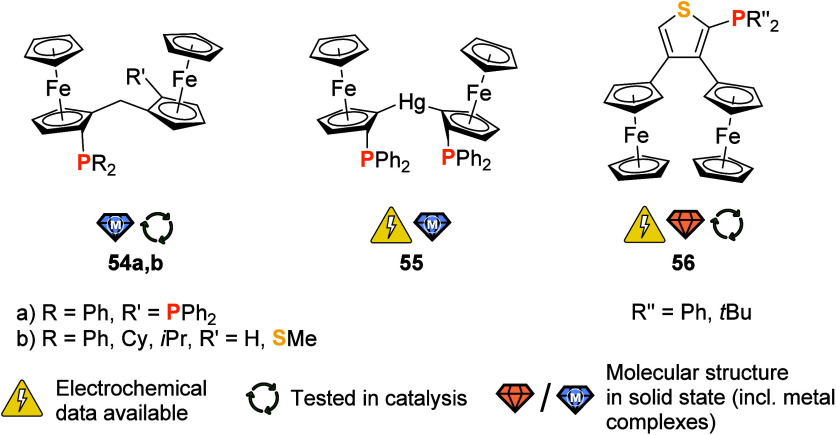
Bis(1,2-disubstituted) ferrocenylene systems **54a,b** and **55**, and (bis(ferrocenyl)thiophenyl)phosphane **55**.

Combining structural motifs of **46** (Figure 7) and **49** (Figure 8), chiral (by combined planar chirality) bis-phosphane **54a** (Figure 9) was first described by Weissensteiner, Spindler, and team in 2006.^200^ Its multistep preparation relied on Kagan’s sulfoxide methodology for preparing the planar chiral precursors which were connected in a key *C*,*C*-coupling step by reacting a lithioferrocene with a ferrocenylaldehyde. The borane adduct of **54a** and its *cis*-coordinated {PdCl_2_} complex were characterized by X-ray crystallography. Unfortunately, **54a** performed with low enantioselectivity in Rh- and Ru-catalyzed asymmetric hydrogenations. Using a different methodology to install planar chirality by way of a chiral hydrazone auxiliary at the C_1_ spacer, Enders and co-workers disclosed a related class of ligands **54b**.^201^ As a result of the synthetic strategy, R′ is a methylthio group, and different phosphanyl residues PR_2_ were introduced. Despite the attractive synthetic access, no further studies regarding the electrochemistry or coordination behavior of these compounds seem to have been reported.

In marked contrast, diferrocenylmercury-bridged bis-phosphane **55**, first reported in 2018 by Jäkle and co-workers,^202^ is well characterized. Notable for combining ambiphilicity through the central mercury link and planar chirality (again, first introduced through the Kagan sulfoxide protocol), **55** was first isolated as its {HgCl_2_} complex. Free **55**, obtained by treating [HgCl_2_(**55**)] with an excess of cyanide, exhibits two well-resolved oxidations in cyclic voltammetry in a supporting electrolyte with a weakly coordinating anion (CH_2_Cl_2_, 0.05 M (TBA)[B{3,5-(CF_3_)_2_C_6_H_3_}_4_], 100 mV/s). Compound *rac*-**55**^203^ was then used to complex two {RhCl(cod)} fragments with short Hg···Rh contacts; similarly, short distances were also observed for complexes of *rac*- and *meso*-**55** with {PdCl_2_} fragments, clearly illustrating the Z-type ligand behavior of the mercury center.^203^ A {Pd^0^(dba)} (dba = dibenzylideneacetone) complex of this ligand was demonstrated to activate the C–Cl bond of CH_2_Cl_2_ at 40 °C.^204^

(Bis-ferrocenyl-thienyl)phosphanes **56** contain two electrochemically distinct ferrocenyl groups due to unsymmetrical 3,4-disubstitution.^205^ Cyclic voltammetry (CH_2_Cl_2_, 0.1 M (TBA)[B(C_6_F_5_)_4_], 100 mV/s) confirms that the two ferrocenyl groups of **56** (R′′ = Ph) can be reversibly oxidized in two separate steps; due to electrochemical follow-up reactivity, only the first oxidation is reversible for the *tert*-butyl derivative. A follow-up spectroelectrochemical investigation of the selenized phenyl derivative **56(=Se)** with R′′ = Ph indicated very weak charge delocalization between the two ferrocenyl groups (Robin-Day class II). Together with monoferrocenylated derivatives, ligands **56** were also tested for their performance in Pd-catalyzed Suzuki–Miyaura cross-coupling reactions, showing moderate performance. Given the nonsymmetric ligand design and thus individually addressable ferrocenyl(ene) moieties, compounds such as **54** and **56** should be considered interesting candidates for multistate redox-switchable catalysis.

**Figure 10:**
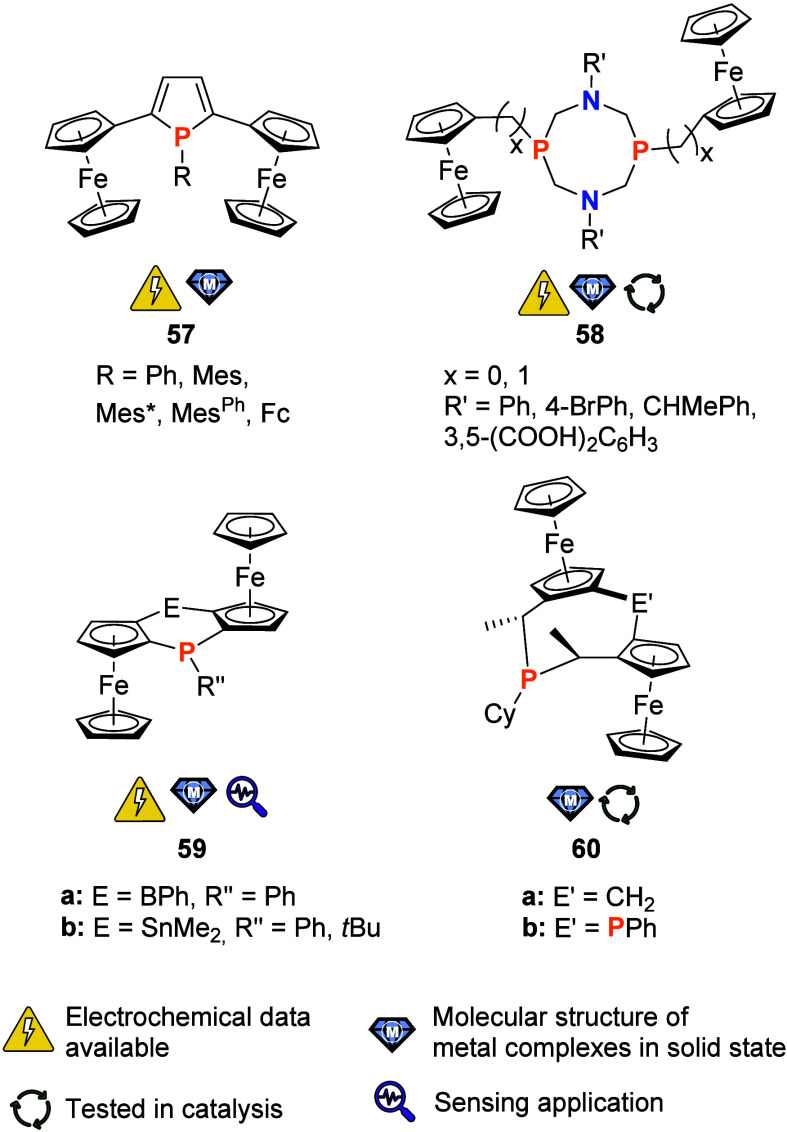
P-cyclic ligands with pendant ferrocenyl groups or ferrocenylene moieties as part of the ring system (Mes* = 2,4,6-tri(*tert*-butyl)phenyl; Mes^Ph^ = 2,4,6-tri(phenyl)phenyl).

The group of P-substituted 2,5-bis-ferrocenyl-1*H*-phospholes (**57**) serves as an entry point into the subset of P-cyclic compounds (Figure 10). The phenyl derivative of **57** was prepared in Lang’s series of multiferrocenylated five-membered heterocycles^12^ to study the heterocycles’ influence on the efficiency of electronic communication.^206^ Several gold(I) alkynyl complexes of **57** (R = Ph) were also prepared.^207^ Upon coordination, the strength of the electronic interaction as assessed by spectroelectrochemistry decreased. Still, both **57** (R = Ph) and its gold complexes could be classified as moderately coupled Robin–Day Class II systems.^206,207^ Transition metal carbonyl fragments were also coordinated to **57** (R = Ph).^208^ Intriguingly, the phosphole’s formal diene backbone was found able to coordinate an {Fe(CO)_3_} fragment when **57** (R = Ph) was treated with an excess of [Fe_2_(CO)_9_], and the degree of communication in the mixed-valent oxidized species was found to depend on whether the P lone pair was engaged in coordination to a metal (or had been sulfurized). The degree of pyramidalization of the P atom in **57**, an important parameter in determining the overall aromaticity of the phosphole, was also studied in response to the introduction of bulky substituents.^209^ By including a P-bound ferrocenyl group, the authors prepared a triferrocenyl derivative that was found to be reversibly oxidizable in three separate steps by cyclic voltammetry (CH_2_Cl_2_, 0.1 M (TBA)[B(C_6_F_5_)_4_], 100 mV/s). Spectroelectrochemical measurements, supported by density functional theory-based calculations, supported the assignment of the whole set of ligands as moderately strong coupled Robin–Day class II systems. The more flattened the phosphole (by action of a bulky substituent R), the stronger the electronic coupling was found to be.

Saturated P_2_N_2_ cycles **58** (Figure 10) were first reported in 2002 by Hey-Hawkins and co-workers.^210^ Water-soluble because of the introduction of two 3,5-bis(carboxyphenyl) residues on the N atoms, **58** (*x* = 1) was also used to prepare water-soluble palladium(II) and platinum(II) dichlorido complexes. In a follow-up study, chiral substituents were installed at the N atoms for an *x* = 0 system of **58**.^211^ In addition to the crystallographic determination of its structure, this derivative was characterized by cyclic voltammetry (DMF, 0.1 M (TBA)[BF_4_], 50 mV/s) and found to be quasi-reversibly oxidizable in a single process. Such **58**-type systems were again taken up by Song et al., who installed bromophenyl residues at the N centers of a **58** (*x* = 0) cycle.^212^ Building on earlier work by Musina and co-workers,^213^ 2:1 (**58**:M) complexes of nickel(II) were found suitable catalysts for electrochemical hydrogen generation in water splitting. While free **58**, in the hands of Song and team, showed complicated and irreversible redox behavior in cyclic voltammetry (CH_2_Cl_2_, 0.1 M (TBA)[PF_6_], 100 mV/s), the nickel(II) complexes showed nicely reversible redox activity and, when trifluoracetic acid was added, H_2_ generation under reductive conditions.^212^ The cathodic potentials necessary for H_2_ generation mean that the redox activity of the ferrocenyl termini cannot be exploited, but other applications in homogeneous catalysis may be possible for this attractive ligand system.

Similar to their Hg-based ligand **55** (Figure 9), Jäkle’s diferrocenophosphaborin **59a** (Figure 10) is an ambiphilic system combining a Lewis-acidic B with a Lewis-basic P site.^214^ Compound **59a** is able to coordinate a {RhCl(CO)} fragment in a *trans*-configured 2:1 (**59a**:M) fashion. When fluoride anions are added to quarternize the boron center, a distinct change in the CO stretching frequency of the rhodium(I) complex fragment can be measured. Furthermore, both free as well as coordinated **59a** display nicely reversible, stepwise, redox behavior as probed by cyclic voltammetry (CH_2_Cl_2_, 0.05 M (TBA)[B{3,5-(CF_3_)_2_C_6_H_3_}_4_], 100 mV/s). Taken together, this makes **59a** a multistimuli-responsive system which has yet to find its applications in, for example, catalysis (for the application of a related nondonor system as redox-switchable chiral anion, see section 3.1). A tin-containing system **59b** (R′′ = Ph) was also reported and showed similar characteristics, and a *tert*-butylphosphane derivative **59b** (R′′ = *t*Bu) is available as well.^215^ In light of the combination of responsiveness to different stimuli, planar chirality and relatively straightforward access with modular design opportunities, **59a,b** are attractive targets for further studies.

Planar and C-chiral P-cycle **60**, available as either methylene- (**60a**) or phenylphosphanyl-bridged (**60b**) derivative, was prepared starting from Ugi’s amine (*N,N-*dimethyl-1-ferrocenylethylamine).^216^ Coordination studies toward {AuCl} fragments showed that both phosphanes can form mono- (**60a**) and dinuclear (**60b**) gold(I) complexes. After halide abstraction using silver(I) salts of weakly coordinating anions, these gold complexes were found to be catalytically active in the asymmetric intramolecular cyclization of γ-allenol with moderate enantioselectivities. In an earlier publication, Hii and co-workers had also briefly evaluated **60b** (termed “Stanphos”) for use in the asymmetric Pd-catalyzed α-hydroxylation of 1,3-ketoesters, albeit with only little success.^217^

**Figure 11:**
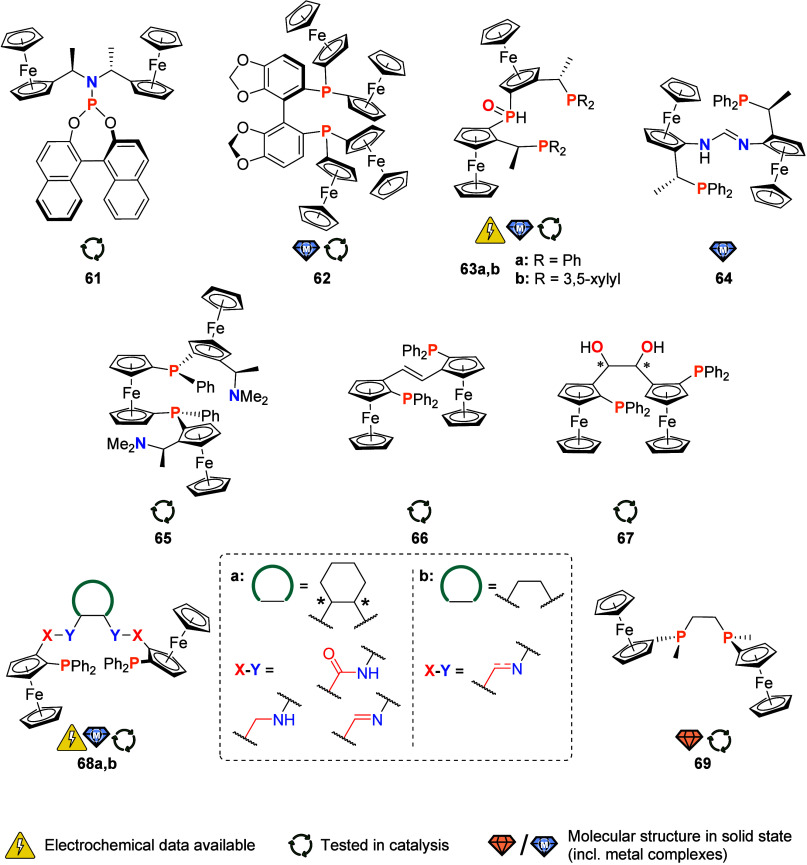
Chiral phosphorus ligands with two or more ferrocenyl or ferrocenylene groups in their backbone (an asterisk denotes C-centered stereocenters; the corresponding diastereomers have been obtained separately from each other).

Aiming for chiral ligands that would support the Cu-catalyzed asymmetric 1,4-addition of organozinc reagents to various nitroalkenes, cycloalkenes, and acyclic enones, Hu and co-workers designed ligand **61** (Figure 11), in which the two ferrocenyl groups serve as bulky substituents for the secondary amine moiety that is integral for obtaining high enantioselectivity.^218^ Indeed, the use of **61** afforded up to 99% enantiomeric excess (ee), a higher ee than obtained with other efficient monophosphoramidite ligands reported at that point.^218^

Compound **62**, a Segphos derivative that contains two bis(ferrocenyl)phosphane donor moieties, was prepared as its racemic oxide.^219^ After enantiomeric resolution using chiral HPLC and reduction with the HSiCl_3_/NEt_3_ system, the axially chiral phosphanes were obtained in enantiomerically pure form. A {PdCl_2_} complex of *rac*-**62** was structurally characterized. (*R*)-**62** was applied as a bidentate ligand in the palladium-catalyzed asymmetric synthesis of axially chiral allenes. An enantioselectivity of up to 92% ee compared favorably to the parent (*R*)-Segphos-derived palladium catalyst with an improvement of up to 18% ee.^219^

Zeng et al. investigated the Co-catalyzed enantioselective hydrogenation of diaryl ketones using ferrocene-based secondary phosphane oxide ligands **63a,b**. These ligands were successfully synthesized from commercially available Ugi’s amine in a multistep procedure.^220^ An unusual P,O bidentate coordination of the ligands with cobalt(II) dichloride was observed, yielding tetrahedral cobalt(II) complexes. When used as catalysts, these exhibited high reactivity and excellent enantioselectivity (up to 99% ee).^220^

Motivated by the establishment of ferrocene-based phosphane ligands in coordination chemistry and their proven success in asymmetric catalysis, Bertogg and Togni combined the amidinato motif with ferrocenylphosphanes in the chiral *N,N*-bis(ferrocenyl)-substituted phosphane-amidine ligand **64**. Deprotonation of **64** and reaction with [Rh_2_Cl_2_(cod)_2_] yielded the corresponding (formamidinato)rhodium(I) complex in good yield.^221^

Spurred by the success of the TRAP^4^ and JosiPhos^222^ ligand families, the continued quest for new chiral phosphanes for use in asymmetric catalysis has also led to the development of several *P*-chiral ligands with more than one ferrocenyl group. Unifying planar-, *C*-, and *P*-centered chirality with a highly modular and scalable synthesis, ChenPhos^6^ combines two ferrocene units in its backbone (Figure 2). Its *C*_2_-symmetric predecessor, TriFer (**65**, Figure 11),^223^ even contains three ferrocene moieties, yet suffers from a complicated and difficult-to-scale preparation.^224^ Both ligands were tested for their performance in asymmetric hydrogenation reactions (in conjunction with rhodium, iridium, and ruthenium), yielding very high ee values.^225^

Kagan and co-workers have reported the syntheses of bis-phosphanes **66** and **67**, featuring a C_2_ backbone. Both compounds can be prepared from a common enantiopure precursor, namely (*S*_Fc_)-2-diphenylphosphanylferrocene carboxaldehyde in a Ti-promoted McMurry coupling (**66**) and a Sm-mediated pinacol coupling (**67**), respectively.^226^ Both bis-phosphanes performed reasonably well in a rhodium-catalyzed asymmetric hydrogenation reaction. The ligand family **68a** (Figure 11), derived from chiral 1,2-diaminocyclohexane by Hou and co-workers,^227,228^ showed high enantioselectivities in palladium-catalyzed asymmetric allylation reactions. Success strongly depended on the right chirality match between ligand and prochiral substrate. An analogue **68b** with a simple C_2_H_4_ spacer replacing the cyclohexane backbone and featuring imine as well as amine linkers was reported by Zirakzadeh and team.^229^ A series of iron(II) complexes, some of which could be characterized crystallographically, was prepared and tested in asymmetric transfer hydrogenation reactions, albeit with moderate success. This work built upon an earlier report of Kagan and co-workers who had used the same *C*_2_-symmetric and planar chiral ligand to prepare copper(I) and ruthenium(II) complexes.^230^ The free ligand is only irreversibly oxidizable as determined by cyclic voltammetry (CH_2_Cl_2_, 0.1 M (TBA)[BF_4_], 100 mV/s). While the ruthenium(II) complex shows quasi-reversible redox behavior, the copper(I) complex engages in irreversible oxidations under these conditions.

In a variation of the very successful DIPAMP-type ligands, Imamoto and team prepared a *C*_2_-symmetric *P*-chiral bis-phosphine **69** employing (−)-sparteine-assisted enantioselective deprotonation of one of the two enantiotopic methyl groups of borane-protected ferrocenyldimethylphosphane.^231^ The solid-state structures of both **69** and its borane adduct were reported. The ligand performed with moderate enantioselectivity in a Rh-catalyzed asymmetric hydrogenation and with a high enantioselectivity of up to 95% in a Pd-catalyzed asymmetric allylic alkylation. While the success of this ligand seems limited, exploring effects of its yet undisclosed electrochemistry on coordination and catalytic activity could provide interesting results.

Introduced by the Togni group in 1995,^8^ the body of literature on **70** in its many variations spans over a decade^232^ in which various substituent combinations R/R′ have been explored and the ligands tested in many, especially Ni-catalyzed, reactions.^233^ It is worth pointing out that, across these studies, the electrochemistry of PigiPhos seems to not have been reported. Given the many available catalytic studies and thoroughly characterized ligands, filling this apparent gap in the literature seems a worthwhile endeavor.

**Figure 12:**
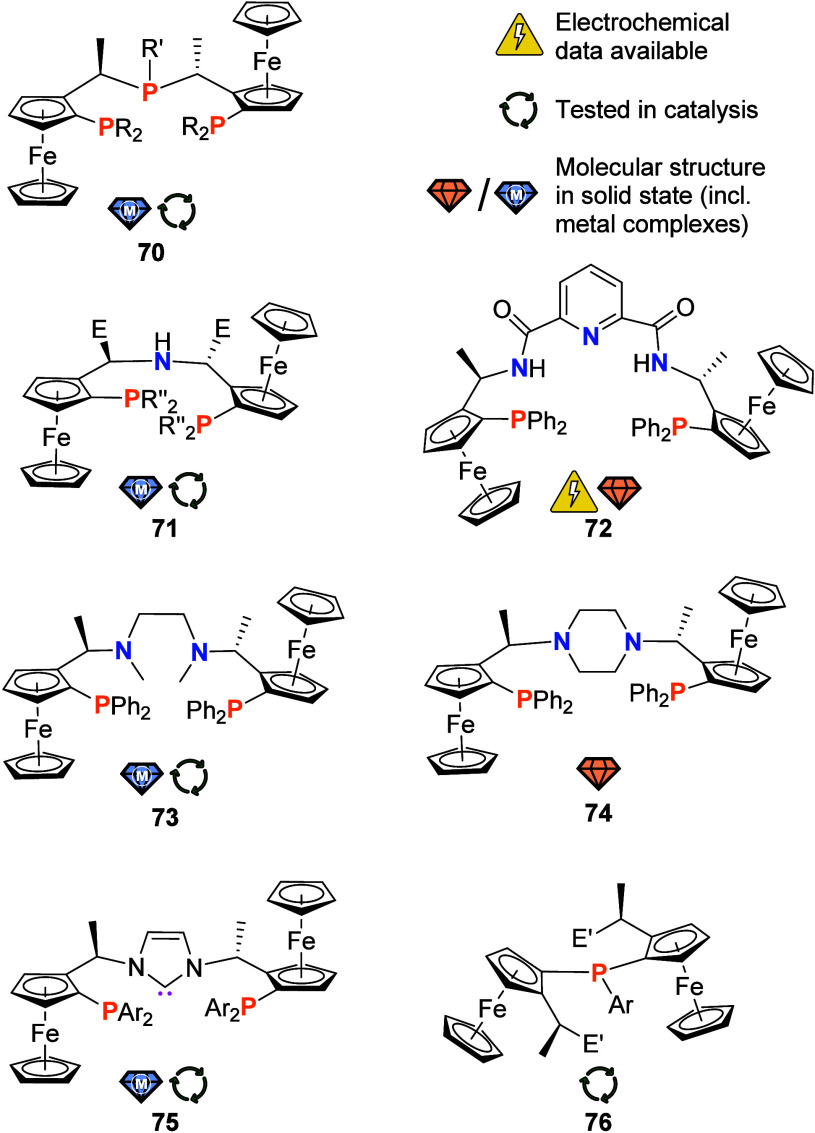
PigiPhos ligand family **70** and related planar chiral (mixed-donor) phosphane ligands (R = alkyl [typically cyclohexyl], R′ = alkyl, aryl; E = H/Me, R′′ = Ph, *i*Pr; E′ = NMe_2_, OMe).

Substituting the central phosphanyl group by alternative donors has opened a rewarding playing field (Figure 12). P,N,P donor **71**,^234^ introduced by Zirakzadeh and co-workers in different substituent combinations including an unsymmetric imine derivative (not shown), has been used to prepare a series of iron(II) complexes. While these partially fluxional complexes, which were also shown to engage in coordination equilibria with CO, have been characterized by variable-temperature NMR as well as Mössbauer spectroscopy, no electrochemical information is available. By expanding the central donor moiety with a bis-amidopyridine group, Kim et al. had attempted to prepare a redox-active ligand **72** for copper(I).^235^ The free bis-phosphane was found reversibly oxidizable during cyclic voltammetry under nonspecified conditions, but **72** underwent N-dealkylation upon addition of a Cu^I^ salt, thwarting further investigations.^235^ More successfully, the same group employed a 1,2-ethanediamine centerpiece in **73**.^236^ Isolation of a tetracoordinate dichloridoruthenium(II) complex and its characterization by single-crystal X-ray crystallography laid the groundwork for the successful application of this complex in an enantioselective cyclopropanation test reaction. The same ligand **73** was also tested for its performance in a copper(I)-catalyzed version of this very reaction. Under these conditions, the PPh_2_ groups were hypothesized to only function as bulky substituents in the key catalytic intermediate,^237^ making **73** an interesting example of a hemilabile ligand likely suited for other catalytic applications.^238^ Preparation of a piperazine-containing derivative **74** and its solid-state structure have been reported as well,^239^ but **74** has yet to find its applications.

Prepared from Ugi’s amine (*N,N-*dimethyl-1-ferrocenylethylamine) in a three-step synthesis, P,C,P donor **75** (Figure 12) by the Togni group was shown to effectively coordinate {Pd^II^Hal} and {Ru^II^Hal(CH_3_CN)_2_} fragments with all three donor atoms in the same plane.^240^ Varying the aryl substituents Ar, the team could also show the corresponding palladium(II) complexes to be efficient catalysts in a hydroamination test reaction, albeit with low enantioselectivity.^241^ Again, no electrochemical data has been made available, and **75** seems to not have been picked up again, a fate that unfortunately holds true for many examples covered in this review.

Before moving to more exotic systems, a brief remark shall be made on **76** which shares a set of similarities with the PigiPhos-type ligands **70** and the chemistry of which has been discussed in connection with the simple Fc_2_PPh parent system **33** shown in Figure 6.^153^

**Figure 13:**
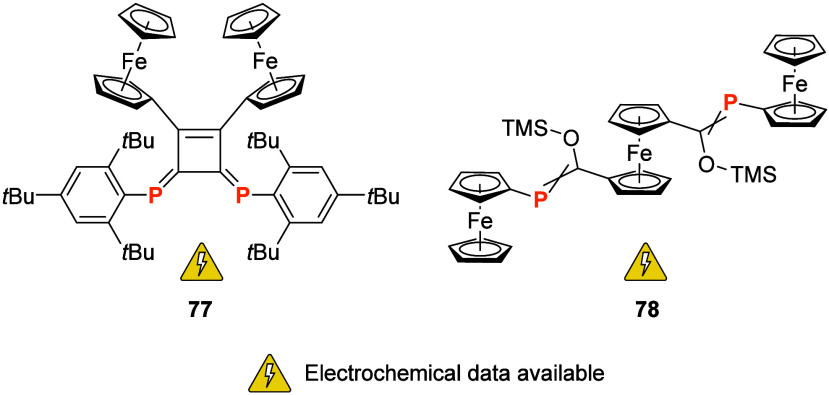
Multiferrocene phosphaalkene systems (**78** was obtained as a mixture of all three isomers, *E*,*E*, *Z*,*Z*, and *E*,*Z* [ordered along increasing stability], as depicted by the crossed double bonds).

Phosphaalkenes^242^ with multiferrocene scaffolds provide an interesting yet underexplored ligand class. Aiming to design multiredox systems, Yoshifuji and co-workers prepared 1,2-diferrocenyl-substituted 3,4-diphosphinidenecyclobutene (**77**, Figure 13) and its transition metal complexes [ML*_n_*(**77**)], ML*_n_* = {Cr(CO)_4_}, {Mo(CO)_4_}, {W(CO)_4_}, {PtCl_2_}, and {PdCl_2_}.^243^ The electrochemical properties of these compounds were studied by cyclic voltammetry (CH_2_Cl_2_, 1 mM (TBA)[ClO_4_], 30 mV/s), revealing two reversible, likely Fe-centered oxidation waves at 0.23 and 0.48 V, indicating considerable electronic interaction between the two ferrocenyl groups.^243^ Chelation of a transition metal affected the interactions between the iron centers in the oxidation process. Accordingly, the cyclic voltammogram of [Cr(CO)_4_(**77**)] (CH_2_Cl_2_, 1 mM (TBA)[ClO_4_], 30 mV/s) exhibited two reversible, Fe-centered oxidations (0.21 and 0.56 V) and one further, irreversible oxidation wave (0.96 V, Cr^0^/^1+^). The results indicate that chelation of Cr affects the second oxidation potential rather than the first. This tendency is also observed for complexes with {Mo(CO)_4_} and {W(CO)_4_} fragments, although the second, Fe-centered oxidation potential appeared to be irreversible in this case, highlighting the complex electrochemistry that may be affected by metal coordination. In complexes with {PtCl_2_} and {PdCl_2_}, both oxidation potentials are affected. These complexes exhibited two reversible oxidation waves (0.45 and 0.64 V for [PdCl_2_(**77**)]; 0.41 and 0.62 V for [PtCl_2_(**77**)]) and one irreversible reduction wave attributable to the Pd or Pt center (−1.06 and −1.47 V, respectively).^243^

A second example is provided by **78**,^244^ a symmetric trinuclear system in which two phosphaalkene units bridge three ferrocene units (Figure 13). Compound **78** was prepared from the reaction between two equivalents of disilylated monoferrocenylphosphane FcP(TMS)_2_ and 1,1′-ferrocenylene bis(carboxylic) acid chloride and was obtained as a mixture of isomers (*E*,*E*, *Z*,*Z*, *E*,*Z*). Analysis of its cyclic voltammograms (CH_3_CN, 60 mM (TBA)[BF_4_], 50 mV/s) shows small differences in oxidation potentials under these conditions. This means that, while two separate oxidations occur, strong charge delocalization in partially oxidized **78** is unlikely.

**Figure 14:**
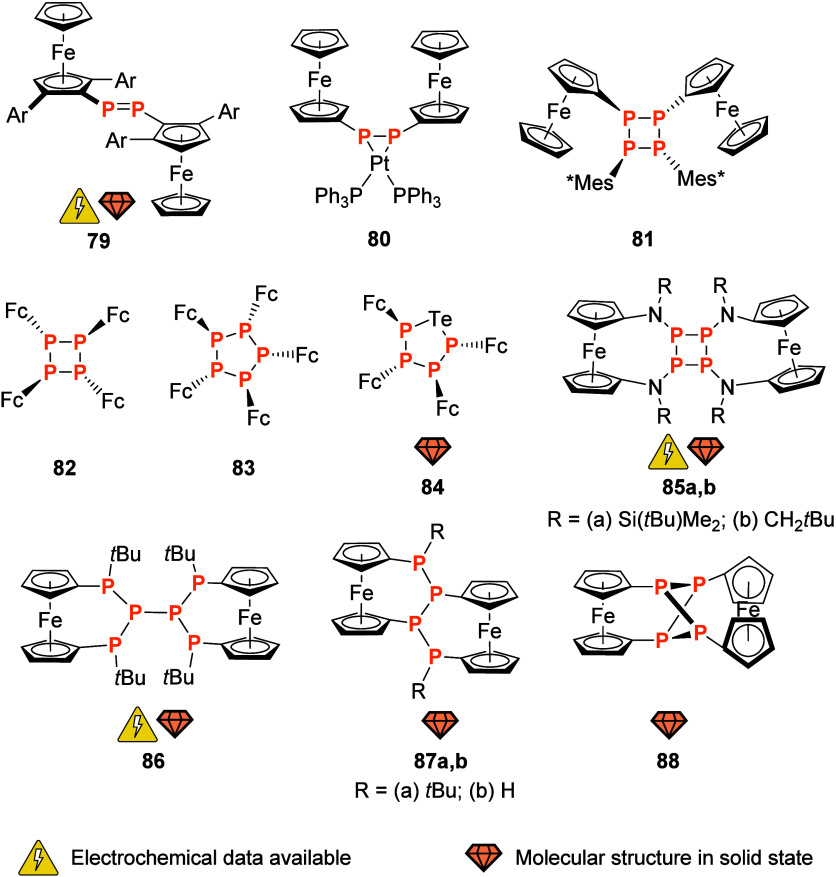
Diphosphene and diphosphane derivatives as well as cyclic and linear polyphosphanes featuring two or more ferrocenyl(ene) groups in their periphery or backbone (Mes* = 2,4,6-tris(*tert*-butyl)phenyl).

Sasamori and Tokitoh have prepared 1,2-bis(ferrocenyl)diphosphene derivative **79** with bulky aryl groups,^245,246^ sporting different substituents (Figure 14). While the coordination chemistry of these diphosphenes has not been explored, examples of related coordination compounds have been described in the literature.^247^ The electrochemistry of **79** shows solvent-dependent behavior, and only the bulkier system with 3,5-bis(*tert*-butyl)phenyl substituents can be reversibly oxidized. Heavier pnictogen congeners of **79** with antimony and bismuth have been explored, too.^248^ Group 14 analogues were reported as well,^249^ including low-coordinate species.^250,251^ None of these have yet been used as ligands.

In an attempt by H. Roesky and team to prepare unsubstituted 1,2-bis(ferrocenyl)diphosphene, only a corresponding and not fully characterized palladium complex **80** gave testimony to the transient existence of related species.^252^ A related monoferrocenylated diphosphene by Pietschnig and Niecke was found to spontaneously dimerize.^253^ P_4_ cycle **81**, containing two ferrocenyl groups, could be split into monomers by action of heat or UV light, but was only characterized by NMR spectroscopy. A fully ferrocenylated P_4_ core **82**, together with its cyclo-P_5_ analogue **83**, was prepared by Wright and co-workers by Sn-mediated dehydrocoupling of the primary phosphine.^254^ Outside the focus of the report, the phosphacycles have not been further characterized. Similarly, Woollins and co-workers reported tetraferrocenylated P_4_Te cycle **84**.^255^ As commonly the case for the compounds assembled in Figure 14, **84** was not tested for its coordinating capabilities, while such ring systems are indeed known to be able to act as ligands.^256^

A more thoroughly investigated system is the P_4_-based bis[4]ferrocenophane **85** by Gudat and Pietschnig prepared by reductive coupling of bis-dichlorophosphanes.^257^ The P_4_ ring geometry depends on the substituents and is found to be either planar (**85a**) or folded (**85b**) in the solid state as probed by X-ray crystallography. Similarly, as shown by cyclic and square-wave voltammetry, the substituents determine whether the Fe-centered oxidations are reversible. Even in the case of **85b**’s quasi-reversible oxidation (*i.e.*, showing the redox potentials and reversibility of the oxidation to depend on scan rates), slower scan rates reveal follow-up reactivity of the oxidized species (EC mechanism).

A family of bis-[3]ferrocenophanes (Figure 14 exemplarily depicts the all-P P_3_–P_3_ derivative **86**) containing several closely related compounds differing in the connecting A_2_B–BA_2_ motif was also reported.^258^ The central bond of the A_2_B–BA_2_ system can be homolytically cleaved when the compounds are heated sufficiently high. Cyclic voltammetry of **86** (THF, 0.1 M (TBA)[PF_6_], 250 mV/s) indicated irreversible oxidation behavior. Additional experimental evidence led the teams of Gudat and Pietschnig to propose the oxidation to proceed via the same short-lived P-centered radicals obtained by thermal bond cleavage.

We finish this short overview about P–P-bonded systems with the bicyclic tetraphosphane **87a** by Pietschnig and Kelemen, obtained by thermal ring-opening of a [2]ferrocenophane.^259^ Very recently, this finding was complemented by a more rational synthesis by reductive cleavage of doubly ferrocenylene-bridged tetracyclic tetraphosphane **88**.^260^ In general, while linear and cyclic oligophosphanes are not commonly used as ligands (selected examples exist),^261^ they can be used as precursors for the use as oligophosphanide ligands.^262^

**Figure 15:**
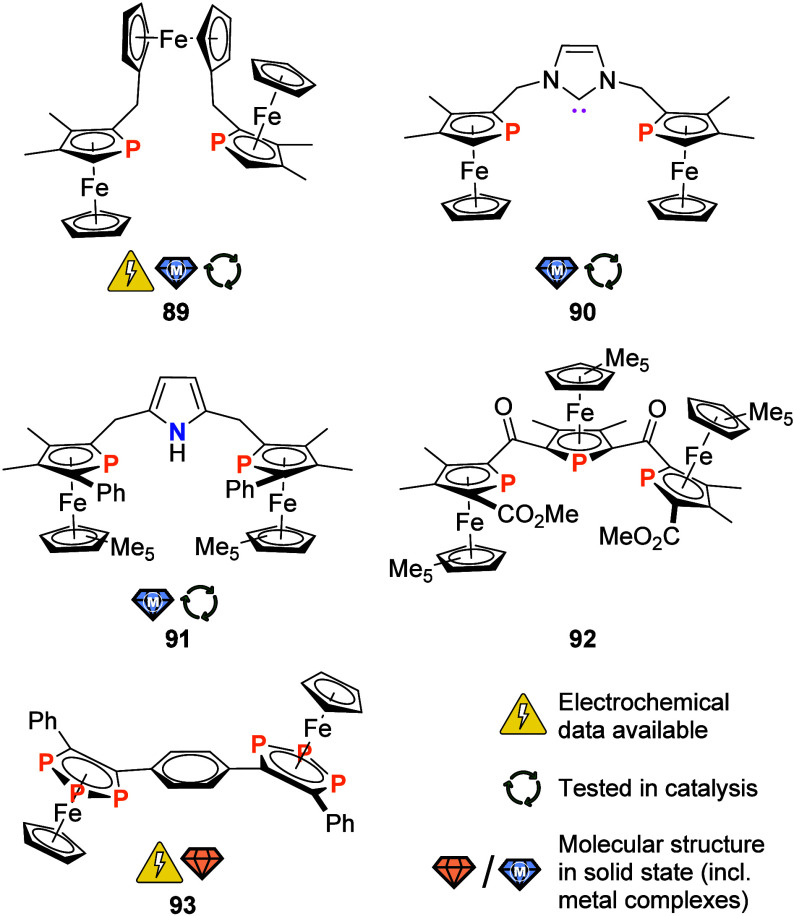
1- and 1,2,3-Multiphosphaferrocenes.

An intriguing way of combining ferrocene and phosphorus lies within the family of phosphaferrocenes.^263^ One can argue whether the phosphaferrocenes assembled in Figure 15 are, in the strict sense, multi-“ferrocene”-containing ligands like the systems discussed so far. Still, they are included nonetheless as they are not only aesthetically pleasing but might also stimulate the reader to think beyond established systems. Bis(phosphaferrocene) **89** was reported by Ganter and co-workers in 1999 and prepared as both a 1:1 mixture of the *rac* and *meso* forms and the enantiomerically pure (*S*,*S*) isomer.^264^ The potential of **89** to act as a chelating agent was demonstrated by preparation of a crystallographically characterized complex featuring a *cis*-coordinated {Mo(CO)_4_} fragment. Cyclic voltammetry (CH_2_Cl_2_, (TBA)[PF_6_] (unspecified concentration), 100 mV/s) shows the central ferrocenylene core to be oxidized first, followed by the two phosphaferrocenyl groups. All oxidations appear reversible under these conditions, and coordination of the molybdenum(0) complex fragment induces the oxidations of the phosphaferrocenyl groups to become distinguishable. When used as a ligand in a Pd-catalyzed asymmetric allylic substitution test reaction, good enantioselectivity of about 80% ee was achieved. Despite all these attractive properties, **89** appears to not yet have been picked up again. A related system in which the ferrocenylene spacer was replaced by an NHC core was reported by the same group.^265^ Together with its monophosphaferrocenylated congener, **90** (and variants with different alkylene spacer lengths) was shown to act as a hybrid P,C ligand for {Mo(CO)*_n_*} and {RuCl(Cp*)} fragments. In the absence of electrochemical characterization, preliminary catalytic studies in a Suzuki–Miyaura *C,C*-coupling reaction hinted at **90** and related ligands to be useful systems prime for further exploration. Similarly, pyrrole-based pincer-type ligand **91** reported by the Mathey group was found able to P,N,P-coordinate a {Rh(CO)} unit (both ligand and complex were obtained as a 1:1 mixture of the *rac* and *meso* forms).^266^ In a hydroformylation test reaction, the **91**/rhodium(I) system outperformed a chemically related 2,4,6-triarylphosphinine but did not reach the same yields as state-of-the-art bulky phosphite rhodium systems. The more complex tris-phosphaferrocene **92** by Carmichael and co-workers, while characterized by X-ray crystallography, remains an interesting curiosity.^267^ Bis(1,2,3-triphosphaferrocene) **93** by Zagidullin and team was characterized by single-crystal X-ray crystallography and solid-state electrochemistry (using modified graphite/phosphonium salt electrodes with about 5% of analyte; the carbon-paste electrodes where then scanned in cyclic voltammetry experiments [CH_3_CN, 100 mV/s]) to prevent solution-induced decomposition of the phosphaferrocenium species.^268^ The authors claim the first oxidation of **93** to be reversible, while the second oxidation leads to follow-up chemistry.

**Figure 16:**
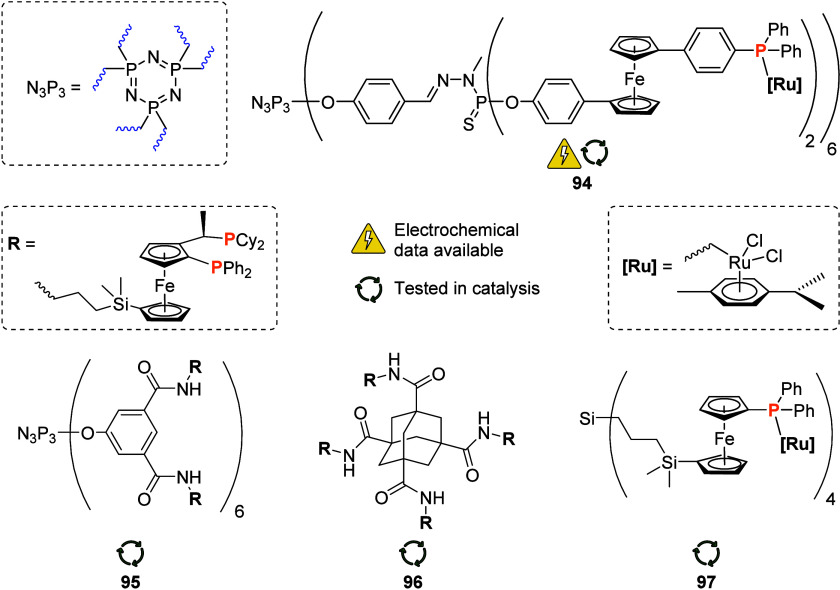
Exemplary structures of dendrimers with terminal ferrocenylene phosphane moieties based on different cores and linkers.^269^

Last, but not least, ferrocenyl-containing dendrimers with accessible phosphane groups such as **94** (Figure 16)^110,269,270^ are multiferrocene phosphane ligands, too. These are potentially interesting for applications in catalysis, since the close proximity and high local concentration of catalytically active sites can be advantageous for some (not all) catalytic reactions when the dendrimer is compared to monomeric species at the same metal loading (positive dendritic effect).^107^ Furthermore, dendrimers can be thought of as bridging the gap between the homogeneous and heterogeneous regimes of catalysis, that is, a means to heterogenize homogeneous catalysts. Accordingly, sufficiently large dendrimers behave as nano-objects which can be retrieved by centrifugation or nanofiltration, allowing for the recovery (and potential reuse) of the catalyst. To that end, the groups of Caminade and Hey-Hawkins have jointly devised a phosphazene-derived ferrocenylene-terminated dendrimer (**94**). In its native form, **94** is an effective catalyst for the redox-isomerization of allylic alcohols to the corresponding ketone. When oxidized, the catalytic activity is strongly attenuated. As this effect is reversible, **94** was recognized as the first redox-switchable dendritic catalyst. For larger dendrimer generations with 24 or 48 ferrocenylene termini (branching units derived from *para*-hydroxybenzaldehyde and P(S)Cl_2_-substituted methylhydrazine as shown in Figure 16), the highly charged oxidized forms even precipitate from the reaction mixture.^99,175^

Similar design approaches had also been used earlier, albeit not for redox-switchable systems. Phosphazene cores have been used to construct dendrimers terminated by Josiphos-type ferrocenylene phosphanes such as system **95** (Figure 16; a larger N_4_P_4_ center allowed the isolation of a dendrimer with 16 termini) by Togni and team.^271^ The Josiphos-type building block **R** was also connected to other cores such as the 4-fold substituted adamantane **96**.^272,273^ In homogeneous asymmetric transition metal catalysis, the Josiphos-type termini allowed for highly enantioselective reaction outcomes, typically with slightly lower ee values than obtained for the mononuclear ligand analogues. The electrochemical behavior of these systems as well as that of a conceptually related carbosilane-based dendrimer **97** by Lang and co-workers^274^ remains to be explored. Connected to the same ruthenium fragment featured in **94**, **97**, and related congeners were tested for their use in β-oxopropyl ester synthesis and found to be suitable as catalysts when solubility of all reaction components could be ensured. Positive dendritic effects were found for polyamide-based systems (not shown) when used in palladium-catalyzed test reactions.^275,276^ Given the high symmetry of the systems covered here so far, truly stepwise control of oxidation states is unlikely. Still, we hope that the potential of ferrocenyl(ene)-rich dendritic systems for redox-switchable applications is clear, even from this brief and necessarily incomplete overview.

### 2.2 N Donor Ligands

**Figure 17:**
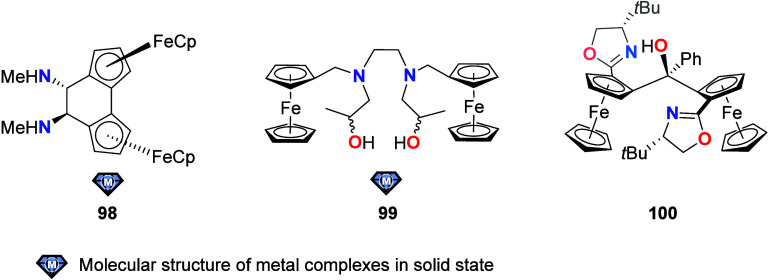
(Potentially) chiral N donors with two ferrocenyl(ene) groups.

**Figure 18:**
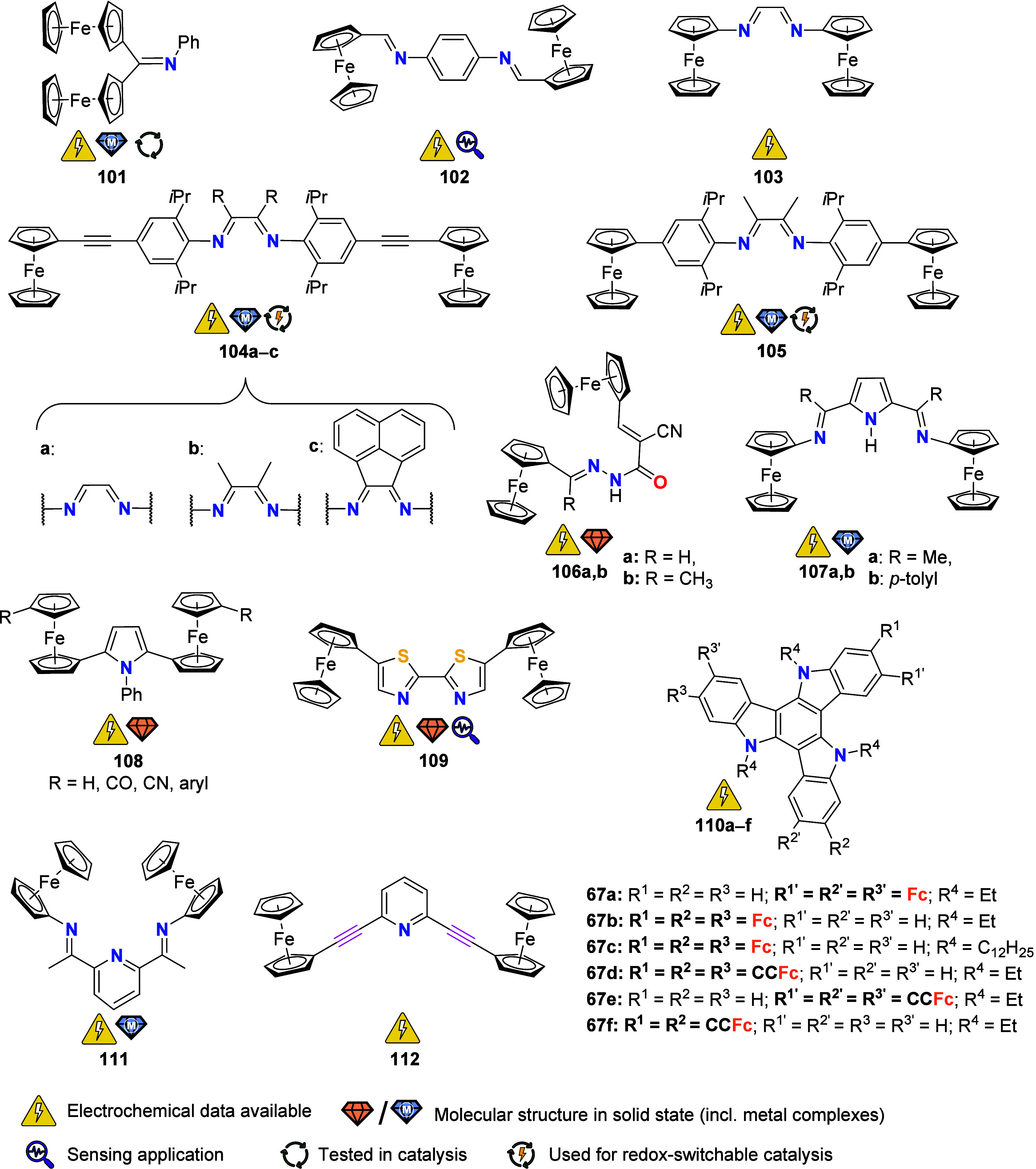
Nitrogen-based ligands with two or more ferrocenyl or ferrocenylene groups in their backbone or as peripheral substituents (part I).

**Figure 19:**
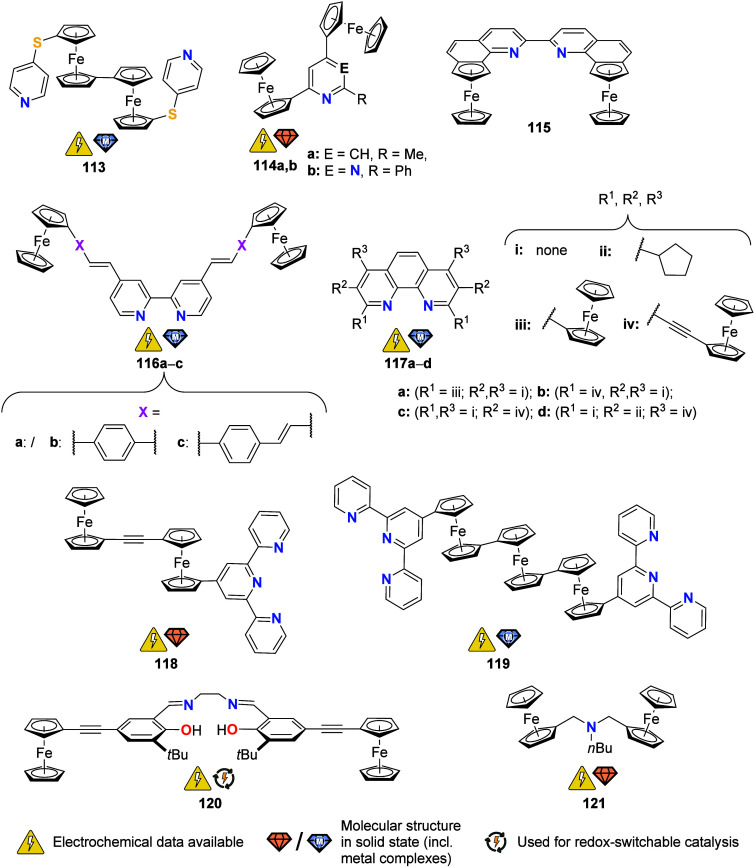
Nitrogen-based ligands with two or more ferrocenyl or ferrocenylene groups in their backbone or as peripheral substituents (part II).

In contrast to the P donor ligands presented in the previous section, examples of structurally characterized metal complexes with N donors are rarer. Many applications of relevant compounds fall, broadly speaking, within the areas of electrochemical and/or UV/vis sensing and frequently only hypothesize or computationally study the underlying ligand–metal interactions. Still, an appreciable number of well-characterized systems exist, and we have tried to cover potential ligands as broadly as possible, aiming to provide the reader with a guide to decide which of the reported compounds might be worth further exploration. Among such (potential) ligands are the (potentially) chiral diamine ligands **98**, **99**, as well as bis(oxazoline) **100** (Figure 17), while the compounds shown in Figure 18 and Figure 19 cover a range of classical N donors ranging from imines over pyridines to triazatruxenes.

Aiming for *C*_2_-symmetric bis(ferrocenyl) ligands, Larsen et al. prepared **98** (Figure 17). They used a Cu^II^-mediated coupling reaction of the lithiated derivative of Kagan’s ferrocenyl acetal, followed by deprotection to yield the biferrocenyl dialdehyde. This intermediate was followingly converted to the bis(methylimine) derivative by reaction with anhydrous methylamine and MgSO_4_. In the final step, the bis(methylimine) was converted to the corresponding bis(methylamine) **98** by a SmI_2_-mediated aza-pinacol coupling reaction in THF.^277^ Compound **98** was used to coordinate {Ti^IV^Cl_2_(OAr)_2_} (OAr = 4-vinyl-2,6-dimethylphenyl), and its doubly deprotonated diol analogue was found to coordinate {Pt^II^(dppe)} (dppe = 1,2-bis(diphenylphosphanyl)ethane). Both complexes were characterized by single-crystal X-ray crystallography. The *C*_2_-symmetric tetradentate N_2_O_2_ ligand *rac*-**99**, reported by Knoesen et al., was prepared from ferrocenylcarboxaldehyde and ethylenediamine followed by reduction of the Schiff base with LiAlH_4_ and subsequent *N*-alkylation with 1,2-propylene oxide. In the corresponding cationic octahedral rhenium(V) oxo triphenylphosphane complex, the tetradentate doubly deprotonated N_2_O_2_ ligand is nonsymmetric, occupying axial and equatorial positions. The structures of free **99** and its rhenium(V) complex were characterized by single-crystal X-ray diffraction.^278^ Carretero and co-workers also reported the synthesis of **100**, a planar and C-chiral bis(oxazoline) (BOX) ligand.^279^ No further studies on **100** appear to have been published, but given that the class of BOX ligands are considered privileged structures for use in asymmetric catalysis,^280^ use of **100** could provide interesting results, specifically in the context of its yet unexplored redox chemistry.

The *N*-(bis(ferrocenyl)methylene)aniline Schiff base (**101**) (Figure 18) was prepared by Suo and co-workers by reaction of bis(ferrocenyl)methanone with aniline and the coordination ability and electrochemical properties were studied. The electrochemical investigation of **101** and its cyclopalladated complex [PdCl(PPh_3_)({(1,2-C_5_H_3_)FeCp}FcC = NPh-κ*N,C*}] revealed that the communication between the two ferrocenyl units was stronger in the palladium(II) complex than in **101**, due to the electron-withdrawing effect of the Pd center. Furthermore, the complex showed excellent performance in Suzuki C–C coupling reactions.^281^ The bis-imine **102** and similar derivatives with different spacers (not shown) were reported by Uahengo et al.; they were obtained by condensation reaction of primary diamines H_2_N-X-NH_2_ (H_2_N-X-NH_2_ = benzenediamine, benzidine, and disulfanediyldiamine) with ferrocene carboxaldehyde. These ferrocene-based Schiff bases were used as chemosensors for the detection of Cu^II^ and Hg^II^ species in aqueous environments.^282^

Diferrocenylated 1,4-diazabutadiene derivative **103** was prepared in 2000 by the Bildstein group during their quest to prepare ferrocenyl-containing *N*-heterocyclic carbenes and was used for complexation of group 6 metal carbonyls.^283^ Although the resulting complexes, not structurally characterized, displayed a very rich electrochemistry, no further investigations into **103** and its coordination behavior were reported. The α-diimines **104a**–**c**^284^ and **105**(15) as well as bis-salicylaldimine **120**(285) (Figure 19) and their corresponding metal complexes (Pd, Ni, and Ti) have proven to be very interesting systems for redox-switchable polymerization catalysis and will be discussed in section 3.2.

Cyanovinylhydrazone-bridged bis(ferrocenyl) compounds **106a,b** as donor–acceptor type derivatives were prepared in good yields by Barik et al. using a solvent-free synthetic method at room temperature with rice husk ash (RHA) as a solid support. The two-step synthesis started with the reaction of ferrocenylcarboxaldehyde (for **106a**) or acetylferrocene (for **106b**) with equivalent amounts of cyanoacetylhydrazide in the presence of RHA. In the next step, the corresponding intermediates were coupled with another equivalent of ferrocenylcarboxaldehyde to give the bis(ferrocenyl) derivatives in good yields. Cyclic voltammetry and differential pulse voltammetry (DPV) in acetonitrile (0.1 M (TBA)[ClO_4_] as supporting electrolyte) showed stepwise oxidation of the two terminal ferrocenyl groups. UV/vis spectroscopy identified compounds **106a,b** to have suitable donor–acceptor properties and, thus, **106b** was used for the fabrication of a solar cell module.^286^

Marks and co-workers prepared a highly conjugated bis(ferrocenyl) pyrrole diimine ligand (**107a**) in order to investigate the influence of the radius of rare-earth ions on metal–metal charge transfer in trinuclear mixed-valent complexes.^287^ A set of 3d-block metal complexes with MCl_2_ centers (M = Co, Fe, Zn) was prepared and structurally authenticated by X-ray crystallography. Cyclic voltammetry of free **107a** (CH_2_Cl_2_, 0.1 M (TBA)[B(3,5-(CF_3_)_2_C_6_H_3_)_4_], 100 mV/s) showed a concerted and reversible two-electron oxidation process, while coordination led to a split into two observable oxidation steps under the same conditions. Spectroelectrochemical measurements on the {FeCl_2_} complex confirmed IVCT to take place in the monocation. In a rare example, the team were able to isolate and structurally characterize a monooxidized cobalt and a fully oxidized iron complex through chemical oxidation with Ag[PF_6_] (M = Co) and [acetylferrocenium][B(3,5-(CF_3_)_2_C_6_H_3_)_4_] (M = Fe). Trinuclear complexes of the deprotonated ligand derivative **107b** with rare-earth metal ions (M = Sc, Y, Lu, and La) featuring two tris(bis-trimethylsilyl)amido ligands were also prepared.^288^ Under these circumstances, the usual rotational freedom of the two ferrocene moieties in mixed-valent complexes, one of which was again structurally characterized, becomes blocked due to ligand–ligand repulsions between the ferrocenium moiety and the N(SiMe_3_)_2_ ligands of the rare earth metal ion. Consequently, the change in rare-earth metal ionic radius became the decisive influence on the strength of the electronic coupling between the ferrocenyl groups upon oxidation.^288^

Hu et al. studied the influence of the heterocyclic spacer in various 2,5-bis(ferrocenyl)-substituted five-membered heterocycles, namely 1-phenyl-pyrrole (**108**), furan, thiophene, imidazole, oxazole, thiazole, 1,3,4-triazole, 1,3,4-oxadiazole, and 1,3,4-thiadiazole, on the intramolecular electronic interaction between two terminal ferrocenyl moieties.^289^ The bis-ferrocene derivatives were prepared by cyclization reactions. Cyclic voltammetry in combination with density functional theory (DFT) calculations indicated that the electronic interaction decreases with the increase of heteroatoms in central heterocycle spacer. Consequently, the efficiency of the electron transfer could be regulated by varying the central heterocyclic spacer.^289^

Caballero et al. reported the synthesis of electroactive thiazole derivatives containing ferrocenyl units (**109**). The general procedure toward 2,5-disubstituted thiazole compounds is based on a two step-synthesis. Acylation of a suitable α-aminocarbonyl compound with the corresponding acyl chloride in the presence of triethylamine yields the corresponding β-ketoamide intermediate. In the following step, the β-ketoamide undergoes a heterocyclization reaction, promoted by the action of Lawesson’s reagent ([4-MeOC_6_H_4_P(S)S]_2_), to give the disubstituted thiazole ring. Structurally characterized 5,5′-bis(ferrocenyl)-2,2′-bis(thiazole) (**109**) was synthesized in 30% yield starting from α-aminoacetylferrocene and oxalyl chloride as acylating agent, and its optical and electrochemical properties were studied. Compound **109** was shown to be a chromogenic sensor for Zn^2+^, Cd^2+^, Hg^2+^, Ni^2+^, and Pb^2+^ ions.^290^

A range of different ferrocenyl (**110a**–**c**) and ferrocenylethynyl-based *N,N′,N”*-triethyltriazatruxene (TAT) (**110d**–**f**) derivatives were investigated by Vogelsang and co-workers. C–C cross-coupling reactions between the appropriate ferrocenylating agent and the corresponding TAT building block,^291^ which itself had already been identified as a three-level redox switch on Ag(111) surfaces, afforded the products.^292^ Suzuki- or Negishi-type cross-coupling reactions yielded the best results for preparing the Fc-TATs with direct linkers. The redox properties and the optical UV/vis- and NIR-excitations in different redox states of compounds **110a**–**f** were investigated. Cyclic voltammetry of **110a**–**f** in CH_2_Cl_2_ in a supporting electrolyte (0.04 M) containing the very weakly coordinating [B{3,5-(CF_3_)_2_C_6_H_3_}_4_]^−^ anion indicated that compounds undergo one reversible one-electron oxidation per ferrocenyl residue, which is followed by one additional oxidation of the TAT core. In-depth UV/vis/NIR spectroelectrochemical studies in conjunction with DFT calculations have been employed to study the stepwise oxidation process, complicated by the extended π system of the core. In the well-studied monooxidized state, intervalence charge transfer seems to play a small role. Compounds **110d** and **110f** were furthermore deposited on a Ag(111) surface and their use as molecular switching units was studied. Interestingly, the ferrocenyl-substituted TATs retained the propensity of the parent *N*,*N*′,*N*″-triethyltriazatruxene (^Et^TAT) for on-surface switching on Ag(111), as probed by scanning tunneling microscope (STM) methods. This renders TATs with appended magnetically anisotropic ferrocenyl substituents viable candidates for magnetically and electrically addressable units in high-density on-surface information storage.^291^

Pyridine is another N-based heterocyclic moiety that has been used for connecting two terminal ferrocene moieties, *e.g.*, in compounds **111** and **112**, or as pendant group in the biferrocene derivative **113** (Figure 19). Diiminopyridine **111**, reported by Magdzinski et al., is a reversibly redox-active ligand due to the pendant ferrocene moieties. It was prepared by a condensation reaction between 1-aminoferrocene and 2,6-pyridinedialdehyde in refluxing ethanol.^293^ Compound **111** shows a reversible two-electron redox process in CH_2_Cl_2_/0.1 M (TBA)[PF_6_] due to the consecutive oxidation of the two ferrocenyl moieties at similar potentials.^293,294^ Complexes of **111** with P^+^, S^2+^, Se^2+^, and Te^2+^^293^ as well as with {GeCl}^+^ and {SnCl}^+^ fragments^294^ were prepared and characterized by X-ray crystallography. Cyclic voltammetry (CH_2_Cl_2_, 0.1 M (TBA)[PF_6_], 500 mV/s) was conducted, showing free **111** to be reversibly oxidizable, while its chalcogen complexes were found to display more complex behavior. Compound **112**, reported by Robinson and co-workers, was prepared in good yield from 2,6-dibromopyridine and ferrocenylacetylene with bis(triphenylphosphane)palladium(II) dichloride and copper(I) iodide (Sonogashira coupling conditions) in diisopropylamine. A single reversible two-electron redox process was observed in the cyclic and square wave voltammogram for **112** in CH_2_Cl_2_/0.1 M (TBA)[PF_6_]. The reaction with [Co_2_(CO)_8_] resulted in the formation of a tetranuclear complex in which one {Co_2_(CO)_6_} fragment is coordinated by one alkyne moiety each, making **112** a C donor in this case.^295^

Biferrocene-based bis-pyridine **113** (Figure 19) has been used to prepare dinuclear (with respect to the coordinated metal) complexes of rhodium and iridium, namely [(*p*-cymene)RuCl(μ-Cl)}_2_(μ-**113**)] and [{Cp*MCl(μ-Cl)}_2_(μ-**113**)] (M = Ru, Ir) some of which have been structurally characterized. These complexes show quasi-reversible redox processes of the bridging biferrocene moiety as well as irreversible redox processes of the terminal metal ions in CH_2_Cl_2_/0.1 M (TBA)[ClO_4_].^198^ Bis(ferrocenyl)pyridine **114a** was prepared in a modular stepwise fashion by Sarkar and co-workers. A ferrocenecarboxamide and acetylacetonate were reacted, installing the first ferrocenyl substituent in 2-position of the pyridine core. Through a nonafluorobutanesulfonyl group in 4-position, further derivatizations were enabled, including the introduction of the second ferrocenyl group through a Negishi coupling.^296^ The synthesis of **114a** demonstrates that the construction of multiferrocenylated (hetero)cycles can also proceed without completely relying on metal-catalyzed cross-coupling chemistry (as shown in Scheme 4). A compound with a donor–acceptor–donor (D-A-D) architecture, 4,6-bis(ferrocenyl)-2-phenyl)pyrimidine (**114b**), reported by Mondal et al., was prepared in good yield in a dehydrogenative coupling reaction from benzamidine hydrochloride, α-methylferrocenemethanol and ferrocenemethanol. The reaction was catalyzed by a ruthenium(II) hydrido chloride complex bearing a bidentate diarylphosphane/phenanthridine ligand which, in conjunction with a Bro̷nsted base, can catalyze multicomponent reactions of alcohols. The lack of near IR (NIR) transitions upon single-electron oxidation implies that the pyrimidinyl unit is less effective at mediating electronic communication compared with pyridine or pyrrole cores.^297^

No coordination chemistry was reported for the structurally appealing ferrocene-annulated bis-quinoline **115** (Figure 19).^298^ Conceptually related 2,2′-bipyridine ligands **116a**–**c** with conjugated spacers of different lengths have, however, been used to form octahedral complexes with zinc(II) and cadmium(II), in which three bipyridine ligands chelate the metal(II) cation (counteranions = [BPh_4_]^−^). The zinc(II) complexes of **116a** and **116c** have been structurally characterized.^299^ In a [PF_6_]^−^-based SE, all complexes display one reversible oxidation for the Fe^II^/Fe^III^ couple and could potentially be used as redox-switchable NLO materials, since they show strong nonlinear optical (NLO) effects^300,301^ in their native states.

1,10-Phenanthroline, which is more rigid than 2,2′-bipyridine, was also employed as a spacer between two terminal ferrocene moieties by Metallinos and Du.^302^ Compound **117a** (Figure 19) was synthesized in good yield by nucleophilic addition of ferrocenyl-lithium (LiFc) to 1,10-phenanthroline.^302^ For the syntheses of **117b**–**d**, a Pd-catalyzed Sonogashira coupling reaction of halogenated-phenanthroline with the corresponding ferrocenylacetylene species was employed.^303−306^ Schmittel and Kishore used to prepare a heteroleptic copper(I) complex^304^ as well as supramolecular fullerene-porphyrin-{Cu(phen)_2_}-ferrocene architectures.^305^ Furthermore, **117c** has also been used by Yuan and co-workers to prepare rare-earth metal complexes [Ln(tta)_3_(**117c**)] (Ln = La, Nd, Eu, Yb; tta = thenoyltrifluoroacetone) from 2-thenoyltrifluoroacetone lanthanides. The presence of the ferrocene moieties in the eight-coordinated, distorted dodecahedrally arranged complex shifts the ligand absorption bands of the rare-earth complexes to longer wavelengths. As a result, the complexes can be excited not only by UV radiation but also by visible light of wavelengths up to 420 nm. Red photoluminescence is observed for the europium(III) complexes and near-infrared photoluminescence for the neodymium(III) and ytterbium(III) complexes.^303^ Compound **117d** was reported by Siemeling and Bausch and was used to prepare a homoleptic zinc(II) complex [Zn(**117d**)_2_][PF_6_]_2_, whose cyclic voltammogram in CH_2_Cl_2_/0.1 M (TBA)[PF_6_] shows noninteracting ferrocenyl units. A four-electron oxidation thus affords the hexacationic [Zn(**117d**)_2_]^6+^.^306^

Terpyridine moieties have also been combined with ferrocene, for example in the bis(ferrocenylalkyne)terpyridine (**118**)^307^ and the triferrocene-derived bis(terpyridine) **119** (Figure 19) (mono- and biferrocene derivatives have also been prepared).^81,308^ 4′-Diferrocenylalkyne-2,2’:6′,2″-terpyridine (**118**) has been used to as tridentate ligand to form octahedral ruthenium(II) complexes. A dramatic decrease of luminescence yields and the triplet lifetimes observed for the ruthenium(II) complex has been attributed to the presence of additional ferrocene moieties, which in this case act as efficient quenchers.^307^ A multinuclear supramolecular assembly from 1,1′-bis(terpyridyl)triferrocene (**119**) and [RuCl_3_(tpy)] (tpy = terpyridine), which form ruthenium(II) tpy terminal groups, was prepared and its electrochemical properties were studied (CH_2_Cl_2_/CH_3_CN (1:1), 0.1 M (TBA)[PF_6_]). Appreciable variations detected in the Fe^2+^/Fe^3+^ oxidation potentials indicate that there is an interaction between the spacer and the terminal Ru^2+^ metal centers, which modulate the electrochemical and spectral characteristics of the triferrocene spacer.^81^ Salen-type ligand **120**, an N,O donor, was prepared by Gibson, Long, and co-workers.^285^ Free **120** did not even need to be isolated; the reaction of an ethynylferrocenyl-substituted salicylaldimine with ethylenediamine was immediately followed by treatment with [Ti(OiPr)_4_] to afford [Ti(**120**-2H)(OiPr)_2_]. Electrochemical data of **120** is thus lacking, but [Ti(**120**-2H)(OiPr)_2_] was characterized by cyclic voltammetry (CH_2_Cl_2_, 0.2 M (TBA)[PF_6_], 50–200 mV/s) and also chemically oxidized using AgOTf. Salen **120** is one of the few examples of multiferrocene-containing ligands used in redox-switchable catalysis (see section 3.2).

The simple bis((ferrocenyl)methyl-*N*-butylamine **121**, prepared from formylferrocene in two steps, should also be mentioned here.^309^ Its molecular structure in the solid state as well as its electrochemistry have been reported. Cyclic voltammetry (CH_2_Cl_2_, 0.2 M (TBA)[PF_6_], 100 mV/s) measurements confirmed the reversible oxidation of both ferrocenyl groups at the same potential, while the amine moiety displays irreversible oxidation chemistry only at higher potentials. Compound **121** could thus be an interesting redox-switchable base for, for example, biphasic catalysis applications, and it might be interesting to study how the presence of the ferrocenium cations would influence the N atom’s basicity.

**Figure 20:**
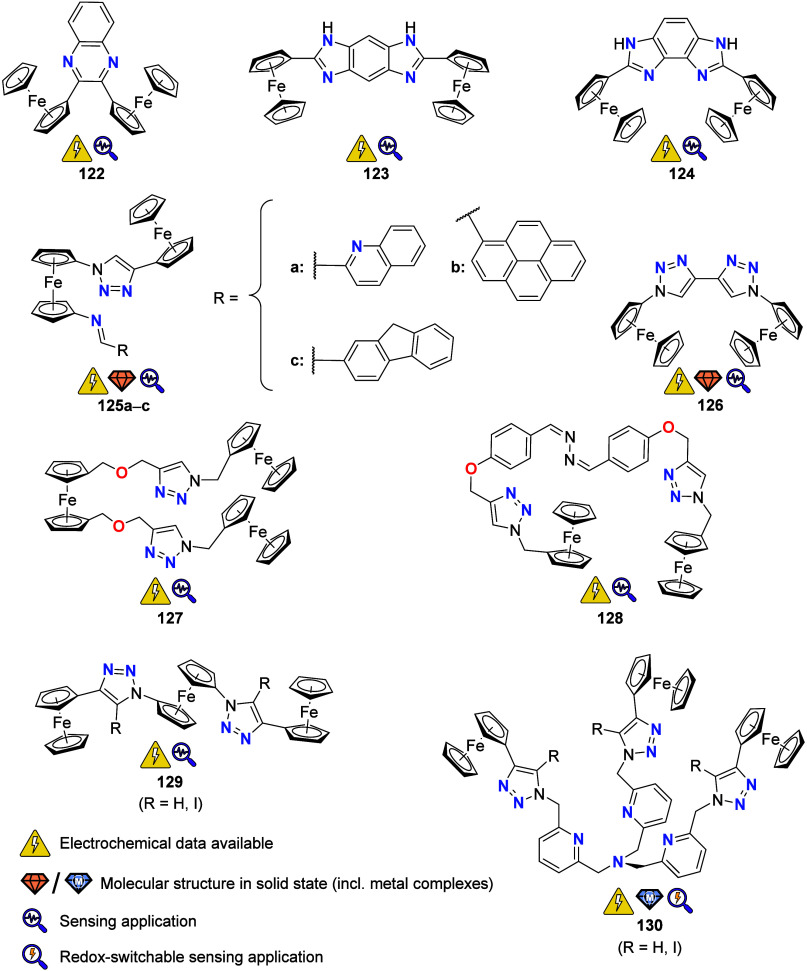
N-Heterocyclic ligands including quinoxaline-, benzobisimidazole-, and triazole-based ferrocene derivatives.

The selective redox and optical properties of ferrocene-containing compounds were also employed for the development of several nitrogen-based chemosensors. The quinoxaline-based derivative **122** (Figure 20), reported by Zapata et al., was synthesized in good yields from condensation of diferrocenylethane-1,2-dione with 1,2-diaminobenzene. It was shown to be a highly selective probe for colorimetric and redox sensing of toxic mercury(II) ions in acetonitrile solutions, as an anodic shift of the ferrocene/ferrocenium oxidation peaks was observed on mercury(II) binding.^310^

The groups of Molina and Tárraga have reported the synthesis, electrochemical, optical, and anion sensing properties of several bis(ferrocene)-benzobisimidazole derivatives.^311−314^ For this, compound **123** (Figure 20) was synthesized in good yield from the reaction of 1,2,4,5-benzenetetramine tetrahydrochloride with ferrocenecarboxaldehyde in nitrobenzene at 80 °C in the presence of triethylamine.^311^ Benzobisimidazole **123** behaved as a highly selective redox, chromogenic, and fluorescent chemosensor molecule for Pb^2+^ and Zn^2+^ cations^311^ as well as for AcO^–^ anions.^312^ The isomeric compound **124** (Figure 20) was prepared starting with the synthesis of a diamine derivative, which was formed through thiadiazole ring opening of 7-ferrocenyl-imidazo[4,5-*e*]-2,1,3-benzothiadiazole by treatment with NaBH_4_ in the presence of CoCl_2_. In the following step, the obtained diamine was coupled with ferrocenecarboxaldehyde to provide **124** in good yield. An easily detectable change in both the redox potential of the ferrocene/ferrocenium redox couple and in the emission band in ethanol solution which is red-shifted (Δλ = 10–13 nm) and enhanced in intensity (chelation enhanced fluorescence, CHEF = 486–225) occurs upon complexation of HSO_4_^–^ and Hg^2+^ ions. Compound **124** thus behaves as a selective redox and fluorescent chemosensor for these analytes.^313,314^

Other important compounds from the class of nitrogen-based oligoferrocene derivatives are the triazole derivatives (**125** and **126**) (Figure 20). These triazoles can be obtained by copper-catalyzed click reaction, in some cases followed by further derivatizations, and were mainly for selective ion sensing. Unsymmetrically 1,1′-disubstituted ferrocenes linked to a 4-ferrocenyl-1,2,3-triazol-1-yl unit and to a functionalized imine group (**125a**–**c**) were synthesized using a tandem click reaction/Staudinger-aza Wittig protocol (to introduce the imino substituent) in good yields. They were shown to have potential as chemosensors for different metal cations, namely Ni^2+^, Cd^2+^, Zn^2+^, and Pb^2+^, through electrochemical and spectroscopic measurements.^315^ Compound **126** and other monosubstituted and disubstituted ferrocene-based triazoles have been reported by the groups of Molina and Tárraga. Like **125a**–**c**, bis-triazole **126** can also be employed as a chemosensor, however, in this case for anions: a cathodic shift of the oxidation peak of the ferrocene/ferrocenium redox couple was observed in the presence of F^–^, AcO^–^, H_2_PO_4_^–^, and HP_2_O_7_^3–^ anions.^316^ Similar to compounds **122** and **123**, the triazole-tethered tris-ferrocene derivative **127**, reported by Ghosh and co-workers, acts as a selective chemosensor for mercury(II), but in aqueous environments.^317^ The 1,1′-disubstituted ferrocene derivative **127** was synthesized in good yields from (azidomethyl)ferrocene and the corresponding dialkynyl ferrocene derivative with CuI/DBU (DBU = 1,8-diazabicyclo[5.4.0]undec-7-ene) as catalyst (click chemistry).^317^ A similar synthetic approach, namely a click reaction of 4-(prop-2-yn-1-yloxy)benzaldehyde with monoazido methyl ferrocene, yields the monoferrocenyl-triazine benzaldehyde intermediate. On further reaction with hydrazine hydrate in ethanol, this intermediate provided the desired acyclic *C*_2_-symmetric azine-bridged bis(ferrocenyl) compound **128** in good yields. Furthermore, Bhatta et al. showed that **128** can also be employed as a chemosensor for Hg^2+^.^318^ Such triazole systems, in general known to show rich coordination chemistry for (transition) metal cations,^319^ can also be used for selective binding to anions such as in the case of **129** (Figure 20).^320^ Developed by Molina and co-workers, **129** was shown to bind oxoanions through noncovalent halogen-bonding interactions, in the case of R = I or hydrogen-bonding interactions for R = H. Changes in the redox potentials as measured by Osteryoung square-wave voltammetry served as the readout, showing differences between various anions relating to their likely binding modes. This concept has been taken up and developed further, for example by Beer and co-workers. Their tripodal receptor **130**, after being preorganized through binding zinc(II) in its N_4_ pocket, can bind anions such as bromide (for R = H) or H_2_PO_4_^–^ (R = I). Its preferentiality for the type of anion is driven by halogen- vs hydrogen-bonding interactions.^321^ Again, making use of the pendant ferrocenyl termini, the interaction was also monitored by differential pulse voltammetry. However, depending on the receptor–anion combinations, electrochemical follow-up reactivity was also observed. The ferrocenyl-iodo-triazole motif has furthermore found application in a polymer recently reported by Su and co-workers (not shown).^322^ Oxidizing the ferrocenyl termini of the polymer led to a marked increase in the strength of the iodo–anion interaction. As demonstrated by the team, this reversible switch in interaction strength was leveraged for selective anion recognition and sorption/release cycles.

Related triazoles (not shown) to those depicted in Figure 20 with two or three pendant ferrocenyl groups have been developed by Kowalski and co-workers.^323^ These triazoles have been positively tested for their antiproliferative activity against lung cancer cells, most likely through the generation of reactive oxygen species which is a well-known mechanism of ferrocene-containing drug-like molecules.^324^

**Figure 21:**
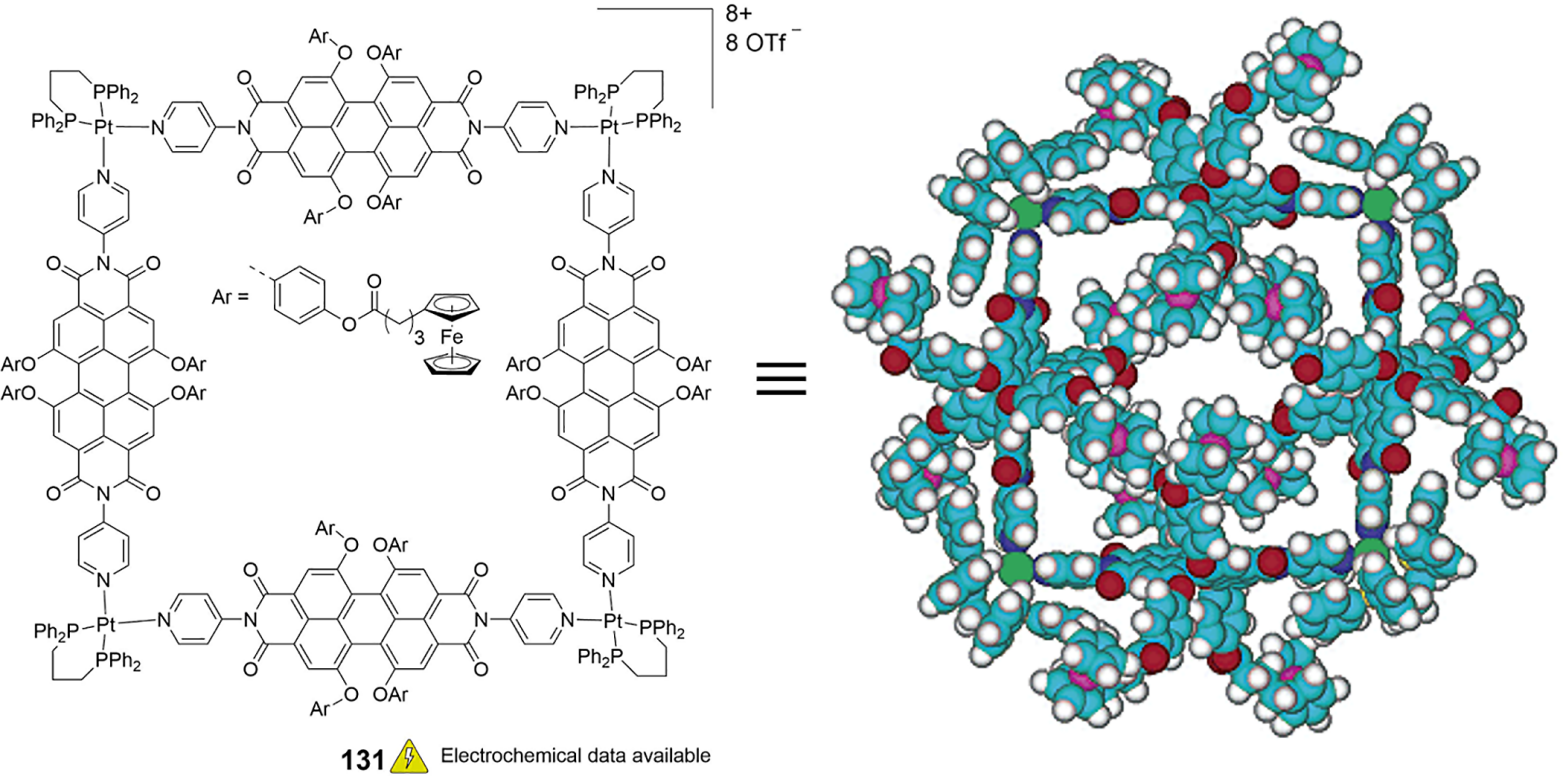
Molecular square **131** based on platinum(II) and tetra-ferrocenylated *N*,*N*′-di(4-pyridyl)perylene bisimide as linker (left, detailed chemical structure; right, space-filling model obtained from a molecular dynamics simulation; color code: C, turquoise; H, white; O, red; N, blue; Fe, lilac; Pt, green; anions have been omitted). Figure adapted with permission from ref (325). Copyright 2003 American Chemical Society.

The tetra-ferrocenylated *N*,*N*′-di(4-pyridyl)perylene bisimide ligand used in the preparation of molecular square **131** (Figure 21) is the final example of this collection of N-heterocyclic moieties. The ligand was synthesized by the coupling reaction of hydroxyphenoxy-perylene bisimides with ferrocenyl carboxylic acids. It exhibits a single, reversible redox wave in cyclic voltammetry (CH_2_Cl_2_, 0.1 M (TBA)[PF_6_]), but when the ligand is included in the molecular square **131**, the redox wave splits up. According to molecular dynamics simulations, eight of the 16 ferrocenyl groups should be located within the cavity of the square, while the other eight ferrocenyl groups are on the outside. Thus, different chemical environments are created, resulting in slightly different electrochemical responses.^325^

**Figure 22:**
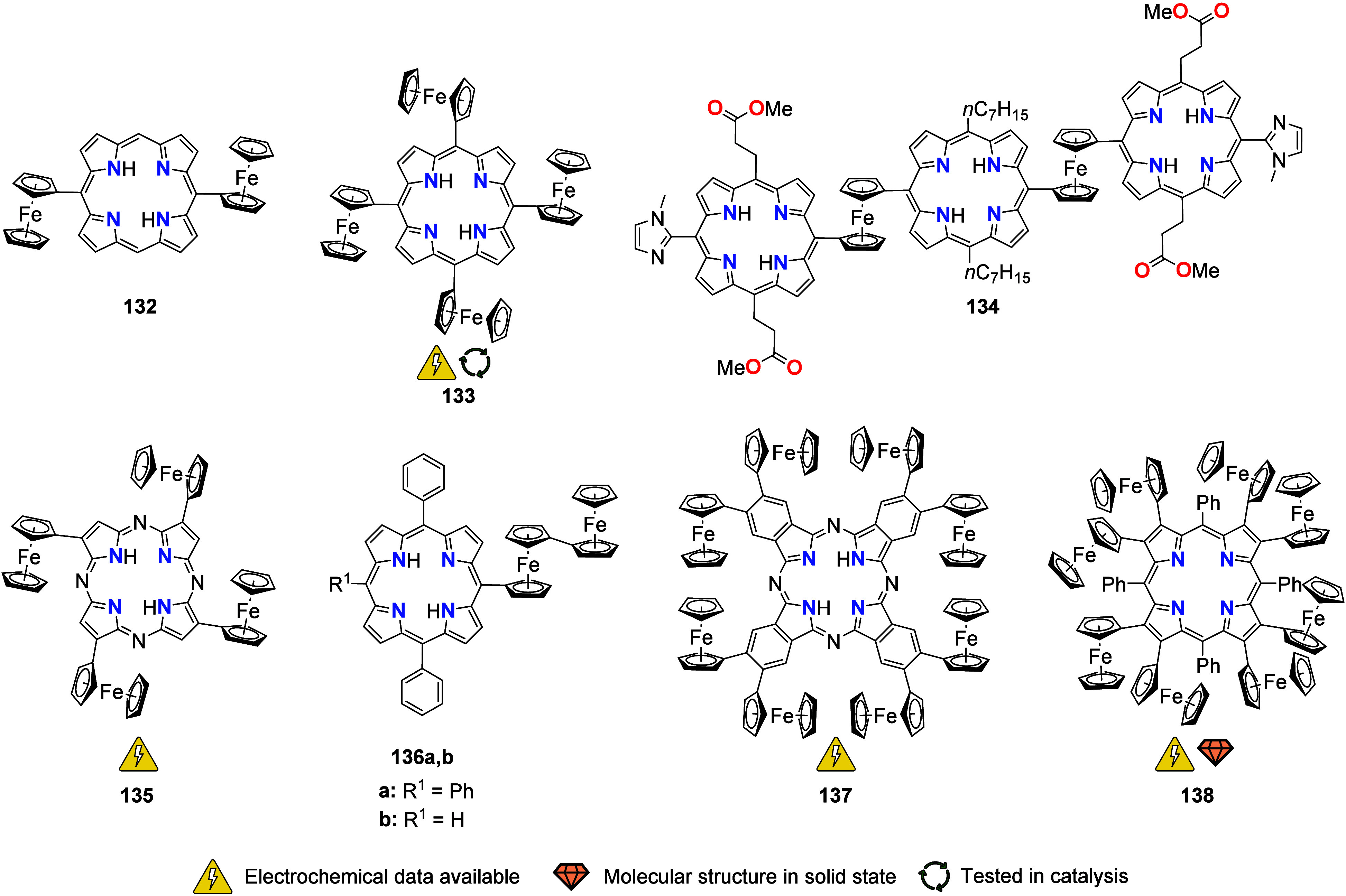
Multiferrocenylated tetrapyrroles.

A special subset of nitrogen ligands is constituted by multiferrocenylated porphyrins and their derivatives (Figure 22), a particularly fruitful area of research reviewed by Bucher and co-workers^326^ as well as, in the broader context of the interplay between organometallics and porphyrins, by Sujikerbujik and Klein Gebbink.^327^ Porphyrins and analogous tetrapyrrole systems such as corroles or phthalocyanines themselves can act as redox-noninnocent systems. Accordingly, studying the electron transfer of mixed-valent porphyrin systems has been of prime interest from the start. This is evidenced by the first example in Figure 22, namely 5,15-bis(ferrocenyl)porphyrin (**132**) and its zinc(II) complex, reported by Zhu et al.^328^ However, *meso*-tetrakis(ferrocenyl)porphyrin (H_2_TFcP, **133**) and the copper(II) complex [Cu(TFcP)] were prepared as early as 1977 by Wollmann and Hendrickson and investigated in that regard. Oxidation of **133** and [Cu(TFcP)] with I_2_ yielded [H_2_TFcP](I_3_)_3_ and [Cu(TFcP)](I_3_)_2_. EPR spectra of these oxidized compounds indicated them to be localized mixed-valence compounds on the EPR time scale. The rate of electron exchange between these pairs of paramagnetic centers is small, and there was no evidence of further interaction between ferrocenium centers from the nearby methine positions, or of interactions between the Cu^II^ ion and a ferrocenium center.^329^ The cobalt(II) complex of **133** was reported in 2014 by Kadish and co-workers. [Co(TFcP)] was found to exhibit six resolved reduction and oxidation events located at cobalt, the porphyrin, and the ferrocenyl groups, and was used for an effective electrocatalytic dioxygen reduction in 1.0 M HClO_4 (aq.)_.^330^

Porphyrins and tetrapyrroles in general can be derivatized before or during the assembly by choosing suitable building blocks. This strategy was used by Wollmann and Hendrickson^329^ and by Kobuke and co-workers^331^ in the preparation of tris-porphyrin **134** (Figure 22). The two 1,1′-ferrocenylene connectors in **134** allowed for the free hinge-like motion around the Cp^R^–Fe–Cp^R^ axes used in constructing even larger, supramolecular assemblies.^331^ The preassembly of suitable building blocks also enabled the preparation of tetraazaporphyrin **135** whose magnesium complex showed interesting UV/vis-spectral properties and was characterized electrochemically.^332^ Alternatively, the macrocycles can be derivatized post-assembly, for example by cross-coupling protocols. Using the Suzuki coupling protocol, the Senge group was able to attach a biferrocenyl moiety to porphyrins (compounds **136a,b** in Figure 22) using iodobiferrocene, paving the way for constructing larger assemblies with intriguing mixed-valent states.^333^

Highly functionalized porphyrins with eight ferrocenyl groups on their rim have also been prepared using both methodologies. Octakis-ferrocenyl-phthalocyanine **137** was synthesized from ferrocenylated building blocks. Its zinc and ruthenium complexes were then assembled into spectacular supramolecular cartwheel structures on a perylene diimide axle.^334^ In contrast, the unusual octakis-ferrocenyl-porphyrin **138** was prepared via an 8-fold Negishi coupling from the corresponding octabrominated porphyrin precursor, a process during which the oxidation to an unusual (since *per se*, non- or even antiaromatic with 4n π electrons) 16π porphyrin was formed. Cyclic voltammetry and square wave voltammetry measurements showed that the consecutive oxidation of **138** to [**138**]^8+^ in seven reversible oxidation steps is possible by using (TBA)[B(C_6_F_5_)_4_] as the weakly coordinating electrolyte, while reduction to [**138**]^4–^ occurs in two irreversible 2-electron steps.^335^

**Figure 23:**
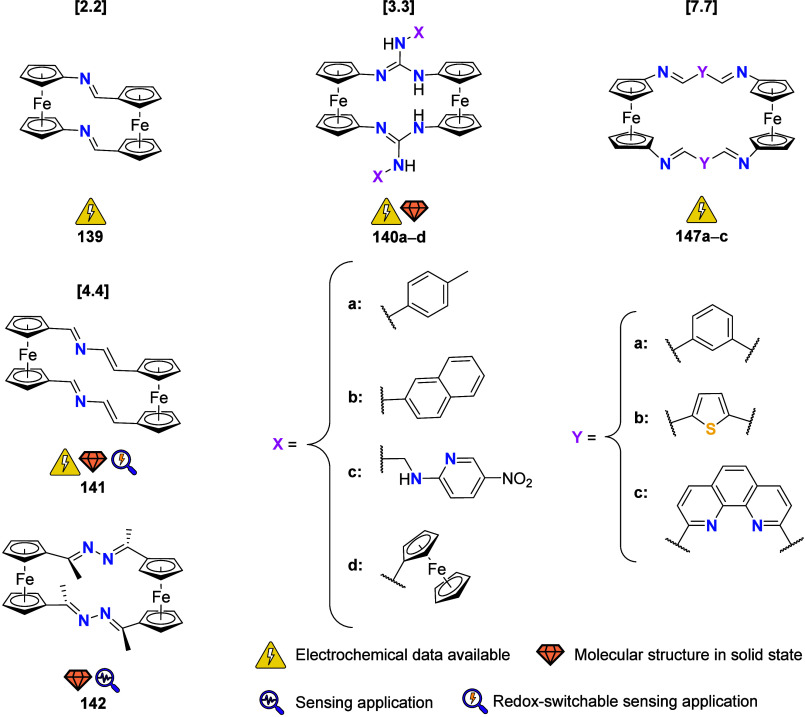
Multinuclear nitrogen-rich [m.m]ferrocenophanes.

Although ferrocenophanes exhibit a diverse chemistry, the exploration of [m.m]ferrocenophanes connected by nitrogen-containing spacers has been limited.^336^ Only a few studies have focused on the synthesis and characteristics of certain multinuclear nitrogen-rich [2.2]- (**139**),^337,338^ [3.3]- (**140a**–**d**),^339^ [4.4]- (**141**),^340^ and (**142**),^341^ as well as [7.7]ferrocenophanes (**143a**–**c**)^342^ (Figure 23).

Compounds **139** and **140a**–**d** were reported by Otón et al. [2.2]Ferrocenophane **139** was synthesized in two steps starting from the bis(iminophosphorane) 1,1′-bis(*N*-triphenylphosphoranylidenamino)ferrocene, prepared via Staudinger reaction between triphenylphosphane and 1,1′-bis(azido)ferrocene. The resulting bis(iminophosphorane) was then reacted with equimolar amounts of 1,1′-bis(formyl)ferrocene via an aza-Wittig mechanism to yield the desired product **139** in good yield. A novel conformationally modulated Intramolecular Electron Transfer (IET) phenomenon has been observed owing to the cyclic structure of the diaza[2.2]ferrocenophane **139**.^337^ Compounds **140a**–**d** with bridging guanidine units were prepared from the bis-carbodiimide 1,3,10,12-tetraaza[3.3]ferrocenophane and the corresponding primary amine NH_2_X (for X, see Figure 23) in good yields. These compounds show remarkable ion-sensing properties due to the combined presence of the redox-active ferrocenylene and an amphoteric binding site (guanidine). The guanidine bridges act as multipoint binding sites for anions, cations, and amino acids. Recognition of anions was confirmed by redox-ratiometric measurements (F^–^, Cl^–^, AcO^–^, NO_3_^–^, HSO_4_^–^, H_2_PO_4_^–^, and HP_2_O_7_^3–^) and colorimetric changes (F^–^, AcO^–^, H_2_PO_4_^–^, and HP_2_O_7_^3–^), whereas the recognition of metal cations (Zn^2+^, Ni^2+^, and Cd^2+^) was studied by electrochemical or fluorescence measurements.^339^ 2,17-Diaza[4,4]ferrocenophane (**141**) was prepared from diethyl aminomethylphosphonate and 1,1′-diformylferrocene. Compound **141** was shown to be a reversibly redox-switchable ion carrier. Mg^2+^ ions are selectively recognized through complexation and released again by an external electrochemical stimulus due to a decreased ion-binding ability of the dication [**141**]^2+^ with respect to neutral **141**. This makes **141** an intriguing system for redox-switchable sensing applications or for enabling metal ion transport across charged/neutral membranes.^340^ Compound **142** was reported by Liu et al., and its binding ability in CH_3_OH was tested for various cations (Fe^3+^, Co^2+^, Cu^2+^, Zn^2+^, Cd^2+^, Hg^2+^, Pb^2+^, and Ba^2+^ [in water]). Receptor **142** demonstrated a strong fluorescence emission at 450 nm when excited at 385 nm. On addition of Fe^3+^, Co^2+^, Zn^2+^, Cd^2+^, Pb^2+^, or Ba^2+^ (dissolved in water) **142** showed no change in the fluorescence intensity at 450 nm. However, there was a notable change when Hg^2+^ (ca. 420 nm) and Cu^2+^ (ca. 490 nm) were added. A reason for these fluorescence changes of **142** could lie in the occurrence of either an electron transfer or an electronic energy transfer involving the transition metal and the excited fluorophore as observed in the other Hg^2+^ and Cu^2+^ recognition sensors. Taken together, these results indicate that **142** could be applied in multianalyte detection.^341^ The last ferrocenophanes of this group are [7.7]ferrocenophanes **143a**–**c**, reported by Sola et al., which were obtained in a similar manner as **139** by aza-Wittig reaction of bisiminophosphoranes derived from 1,1′-bis(azido)ferrocene, PBu_3_, and the corresponding dialdehyde. Like the other ferrocenophane receptors discussed above, these compounds behave as efficient electrochemical and chromogenic chemosensors, here for Zn^2+^, Pb^2+^, and Hg^2+^ metal cations.^342^ More recently, Long and co-workers have also reported on diethynylarene-bridged ferrocenylene- and biferrocenylene-containing macrocycles featuring pyridinylene spacers. While these expanded ferrocenes (not shown) have been studied electrochemically, their coordination chemistry remains to be explored.^343^

**Figure 24:**
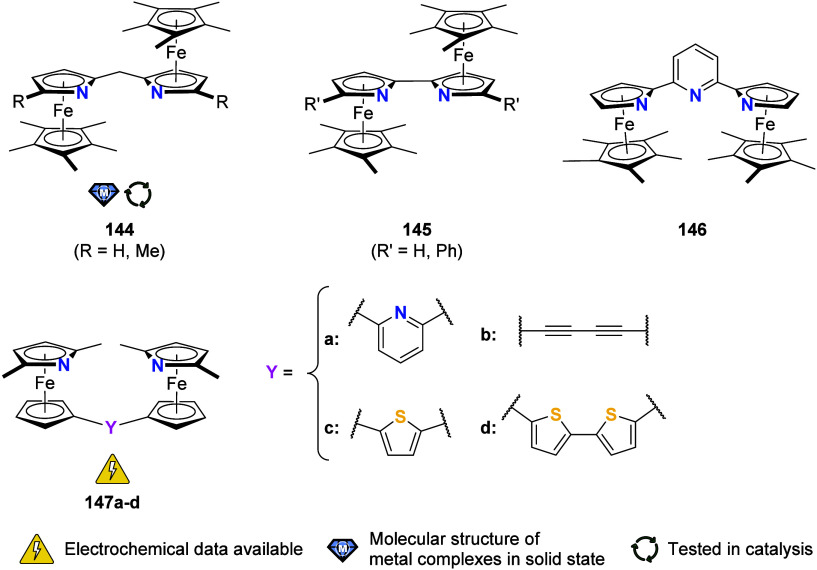
Bis-azaferrocene ligands.

Similar to phosphaferrocenes (s. Figure 15), the small class of azaferrocenes (Figure 24) deserves a special mention. A 2010 review by Kowalski^344^ gives an excellent overview about the synthesis and properties of this substance class. Here, we will only focus on the few selected examples of the multiazaferrocene subset which, to the best of our knowledge, has not been expanded further since. First disclosed in 1998 by Fu and co-workers, **144** (R = H) was prepared as an enantiopure *C*_2_-symmetric ligand exploiting the planar chirality of the ready-made dilithio salt of di(2-pyrrolyl)methane.^345^ A copper(I) complex of **144** (R = H) was crystallographically characterized. Such copper complexes were found efficient and highly enantioselective catalysts for the asymmetric cyclopropanation of substituted olefins as well as for the asymmetric synthesis of β-lactams from alkynes (R = H, Me).^346^ More transition metal complexes featuring nickel(II) and palladium(II) fragments were reported by Salo and Guan.^347^ Their work also included unsymmetric derivatives made up of differently substituted azaferrocene moieties (*e.g.*, with a pentaphenylcyclopentadienide ligand). All systems were then investigated for their use in ethylene oligomerization, in which they performed worse than related diimines. An alternative synthetic route to **144**, **145** (R = H, Ph), and **146** was later developed by Anderson and co-workers.^348^ Using (−)-sparteine and *s*-BuLi, enantioselective deprotonation in the 2-position of a monoazaferrocene becomes possible, allowing for Grignard-type follow-up chemistry and efficient *C*,*C*-coupling reactions. Despite the similarity of **145** and **146** to successful N donor classes, these attractive potential ligands seem to not yet have been investigated for their coordination behavior or application in catalysis.

A series of bis(2,5-dimethylazaferrocenyl)-substituted compounds **147** with various spacers (Figure 24) has been prepared by Kowalski and Winter.^349,350^ The electrochemical behavior of these systems was investigated while changing the spacer Y from pyridylene (**a**) over buta-diyne (**b**) to thienylene (**c**, **d**). While the oxidation of azaferrocenes is, in general, much less reversible than for the corresponding ferrocenes,^344^ 2,5-dimethylazaferrocenes show increased stability for their oxidized forms. Solvent and electrolyte choice as well as measurement temperatures and scan rates have a significant influence: Kowalski found that a 0.2 M (TBA)[PF_6_]/CH_2_Cl_2_ supporting electrolyte yields significantly more reversibly appearing cyclic voltammograms for **147** (**Y** = **a**, **c**, **d**) than the corresponding [ClO_4_]^−^-based supporting electrolyte.^350^ Accordingly, reversible double oxidation of these systems becomes feasible on the CV time scale and the monocations show signs of increased stability due to electronic communication. These complications might prohibit the immediate application of multiazaferrocene-based systems in redox-switchable applications, but the redox behavior of more sterically congested systems and the influence of engaging the N atoms in coordinative bonds might open new avenues for further exploration.

### 2.3 O, S, Se, and Other Chalcogen Donor Ligands

While phosphorus and nitrogen ligands with two or more ferrocenyl groups are comparatively well represented in the literature, examples of oxygen and sulfur donors are scarce, and those with selenium and tellurium as donor atoms are even rarer.

**Figure 25:**
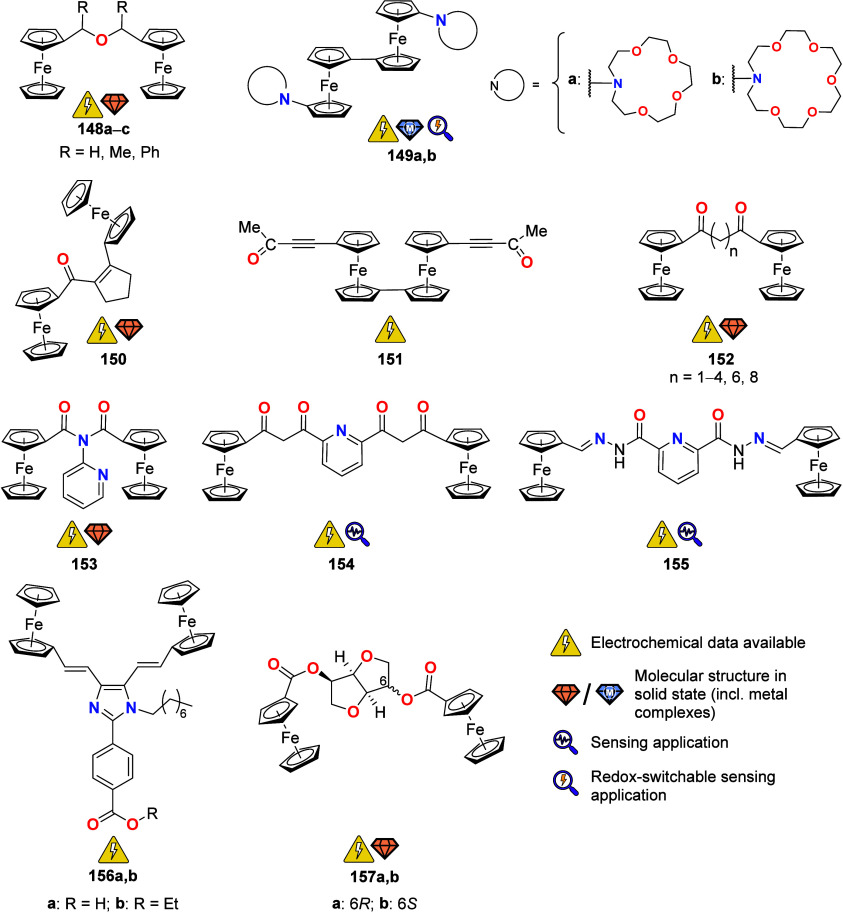
Oxygen-based ligands (ethers, carboxylic acids, and esters, ketones, and diketones) with two ferrocenyl or ferrocenylene groups in their backbone or as peripheral substituents.

The group of oxygen-based oligoferrocenyl derivatives comprises ethers, crown ethers, ketones, diketones, carboxylic acids and esters (Figure 25). Within this set, bis(ferrocenyl) ethers are an especially important class of compounds. Prabhakaran and co-workers reported the synthesis, characterization and electrochemical properties of compounds **148a**–**c**. The three symmetrical bis(ferrocenyl)ethers were prepared by the *in situ* reaction of formyl-, acetyl-, or benzoyl-ferrocene with tris(2-aminoethyl)amine in methanol, using sodium borohydride as reducing reagent. The ferrocenyl ethers **148a**–**c** exhibited diverse redox behaviors, showcasing both reversible and irreversible oxidation and reduction responses at different potentials. These findings highlight the potential use of ferrocenyl ethers in various electrochemical applications.^351^ Next to biferrocene-decorated cyclodextrins by the Astruc group (not shown),^352^ the other examples of ethers with at least two ferrocenyl groups are constituted by the biferrocene-derived tetra- and pentaoxo azacrown ethers **149a,b** (Figure 25).^353^ The Dong group demonstrated the use of **149a,b** as cation sensors; their redox potentials shifted appreciably upon the addition of the alkaline or earth alkaline metal cations.^353^ Furthermore, the unusual increase in affinity toward Ca^2+^ upon mono-oxidation of **149b** (which, naively, would be expected to decrease due to electrostatic repulsion) makes this system a particularly remarkable one, opening possibilities for redox-switchable sensing applications. The solid-state structures of sodium and potassium complexes of **149a** were also reported. Both azacrown ethers do also bind silver(I), calcium, and barium cations.^353^

Several bis-ferrocene-containing ketones were reported. The reaction of ferrocene with adipoyl chloride under Friedel–Crafts conditions with aluminum chloride as catalyst, followed by internal aldol condensation and elimination of water to form the double bond in the cyclopentene moiety, gave 1-ferrocenylcarbonyl-2-ferrocenylcyclopentene **150** in good yields. Electrochemical studies of **150** in acetonitrile with (TBA)[ClO_4_] as supporting electrolyte suggested that the ferrocene units were not in direct electronic communication. Compounds like **150** were used as starting materials for the synthesis of ferrocene-containing polymers; no coordination studies were performed.^354^ Another type of ketone featuring a conjugated 1,1″-biferrocene-1′,1‴-dialkyne derivative (**151**, Figure 25) was obtained by Bennett and Long in the attempted synthesis of a biferrocene disubstituted with alkynyl(phenyl)thioacetates, as molecules containing terminal thioacetates are often used for binding to metallic electrodes. In addition, thioacetates can also act as O donors (see **164** below in Figure 27).^355^ The reaction of diethynyl biferrocene with 4-iodophenylthioacetate under Sonogashira cross-coupling reaction conditions (CuI, [PdCl_2_(PPh_3_)_2_], diisopropylamine (DIPA)) led to the formation of a biferrocene diynone (**151**).^356^ The target compound ferrocene-(ethynylphenyl)thioacetate could, however, be obtained using a different strategy, namely reacting 1,1‴-diiodobiferrocene under optimized Sonogashira cross-coupling conditions with 4-(cyanoethylthio)ethynylbenzene. This synthetic strategy was adopted for the synthesis of other multiferrocene alkyne compounds; an example is compound class **164** shown in Figure 27.^356^

Ferrocenyl-containing diketones are also an important class of oxygen-containing molecules with potential applications as ligands. The group of Fourie has reported the synthesis, characterization and redox properties of β-diketone **152** (*n* = 1), which was obtained from acetyl ferrocene and methyl ferrocenoate in good yield in the presence of lithium diisopropylamide (LDA).^357^ Furthermore, Bulut and co-workers reported the synthesis of **152** (*n* = 2, 3, 4, 6, and 8) via the reaction of ferrocene with the corresponding acid chlorides using EtAlCl_2_ as catalyst, showing that alkyl Lewis acids can be efficient catalysts for the diacylation of ferrocene.^358^ Ferrocenyl-containing diketones featuring additional donor groups, *e.g.* pyridine (**154**) and amino (**153**) or hydrazone (**155**) moieties, offer even more possibilities for the coordination of metal atoms than just β-diketones. Calhorda and co-workers have reported the synthesis of *N,N*-bis(ferrocenecarbonyl)-2-aminopyridine (**153**) from ferrocenyl acyl chloride and 2-aminopyridine in the presence of triethyl amine. The corresponding Mo^II^ complex [MoBr(η^3^-C_3_H_5_)(CO)_2_(**153**)] was prepared; based on the spectroscopic data, complemented by DFT calculations (a solid-state structure of free **153** was reported), a bidentate binding mode of molybdenum(II) of **153** via the pyridine nitrogen atom and one keto group was suggested. Electrochemical studies in CH_2_Cl_2_ with 0.2 M (TBA)[PF_6_] as supporting electrolyte showed that **153** undergoes a single two-electron oxidation generating the partially stable dication [**153**]^2+^. In contrast, the corresponding complex [MoBr(η^3^-C_3_H_5_)(CO)_2_(**153**)] undergoes a multielectron oxidation, which, by exhaustive oxidation, releases the Mo complex fragment generating the partially stable dication [**153**]^2+^.^359^ Two other ferrocenyl diketone derivatives containing pyridyl moieties (**154** and **155**) have been reported by Tian et al.^360^ 1,1′-(2,6-Bispyridyl)bis-3-ferrocenyl-1,3-propanedione (**154**) was synthesized via condensation of two equivalents of acetylferrocene with dimethyl-2,6-pyridine-dicarboxylate in ethanol, while ferrocenecarboxaldehyde-2,6-dipicolinoyhydrazone (**155**) was prepared by reaction of 2,6-dipicolinoyhydrazine with ferrocenecarboxaldehyde in refluxing ethanol. The electrochemical properties and ion sensing abilities of **154** and **155** were investigated by cyclic voltammetry in ethanol. Among the tested set of metal cations (Cd^2+^, Co^2+^, Cu^2+^, Hg^2+^, Mn^2+^, Ni^2+^, Zn^2+^), **154** was found responsive to Cu^2+^ and Mn^2+^, and **155** to Hg^2+^ and Mn^2+^. These results indicate their potential for applications in electrochemical sensor technology.^360^

The last group of ligands to be mentioned here are carboxylic acids and esters. The conjugated bis(ferrocenyl) Y-shaped acid (**156a**) and ester (**156b**) were reported by Prabu et al.^361^ Their redox potentials were examined with cyclic voltammetry in CH_2_Cl_2_ with 0.1 M (TBA)[ClO_4_] as supporting electrolyte. The oxidation potentials of compounds **156a** and **156b** were observed in the range of 0.66 to 0.73 V, which indicates a one-electron charge transfer from ferrocene to ferrocenium ion. These potentials were employed to calculate the related energy gap. The ester (**156b**) was synthesized via condensation reaction between (1*E*,5*E*)-1,6-bis(ferrocenyl)-hexa-1,5-dione, ethyl 4-formylbenzoate and *n*-octylamine, in the presence of ammonium acetate and acetic acid. The hydrolysis of **156b** with 1 N NaOH solution in methanol yielded the carboxylic acid **156a** in good yield. These “push–pull” compounds have been studied for applications in second-order nonlinear optics (**156b**) and dye-sensitized solar cell (**156a**).^361^

The chiral isomannide- and isosorbide ferrocenyl diesters (**157a** and **157b**) were prepared via double esterification of the free hydroxy groups with ferrocenecarboxylic acid in the presence of *N*-ethyl-*N*′-(3-(dimethylamino)propyl)carbodiimide (EDC) and *N*,*N*-4-(dimethylamino)pyridine (DMAP). The structures of the products were confirmed by X-ray diffraction analysis and their electrochemical properties have been studied. The crystal structures of both bis(ferrocenyl) diester complexes showed that the chirality of the bridge results in an open or tight helical crystal packing. With isomannide as central scaffold (**157a**), the ferrocenyl moieties tended to be parallel, forming a chiral pocket, while with isosorbide as chiral scaffold (**157b**), an open structure was observed. Two separate and reversible oxidations were found for both diesters using cyclic voltammetry (CH_2_Cl_2_, 0.04 M (TBA)[B(3,5-(CF_3_)_2_C_6_H_3_)_4_]); spectroelectrochemical measurements indicated that the degree of electronic communication between the ferrocenyl groups was very low in the mixed-valent state. Coordination studies with these esters have not been reported.^362^

**Figure 26:**
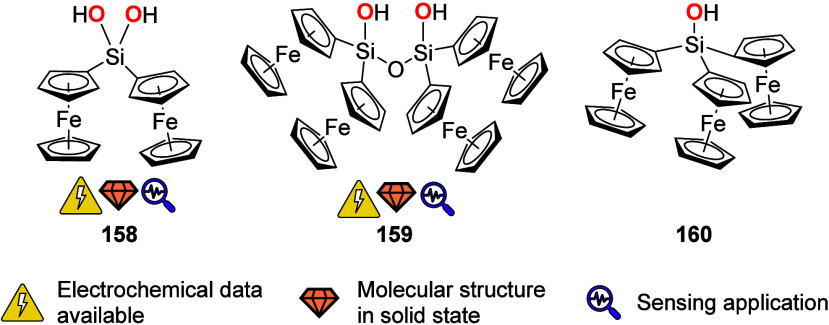
Multiferrocenyl silanols.

Next to carbon-based oxygen donors, multiferrocenyl silanols have also found their way into the literature. First rationally prepared by Manners and co-workers in 1998,^363^ silanediol **158** and disiloxanediol **159** (Figure 26) were characterized in their a solid-state structures and were later found to condense into a hexaferrocenyl cyclotrisiloxane (*i.e*., a Si_3_O_3_ core).^364^ This derivative was electrochemically investigated by differential pulse voltammetry, leading to the conclusion that all six ferrocenyl termini were oxidized in two distinct steps. In a follow-up study, the Cuadrado group, who also prepared tris-ferrocenyl silanol **160**, explored the redox chemistry of **158** and **159** in greater detail.^365^ In cyclic voltammetry experiments, **158** was found to undergo two well-separated and reversible redox processes (CH_2_Cl_2_/CH_3_CN, 0.1 M (TBA)[PF_6_], 100 mV/s), while **159** displayed irreversible oxidations under the same conditions. Changing the supporting electrolyte system to CH_2_Cl_2_/(TBA)[B(C_6_F_5_)_4_], *i.e*., introducing a more weakly coordinating anion, **159** was found reversibly oxidizable in four well-resolved oxidation events. Both **158** and **159** were found to coordinate anions, a process which the team monitored with NMR spectroscopy and square-wave voltammetry. Solid-state structures of anion adducts further supported the Si–O(H)···anion interactions. While silanolates have also been shown to make excellent ligands,^366^**158**–**160** seem to have not yet been tested for their complexation behavior toward (transition) metals.

**Figure 27:**
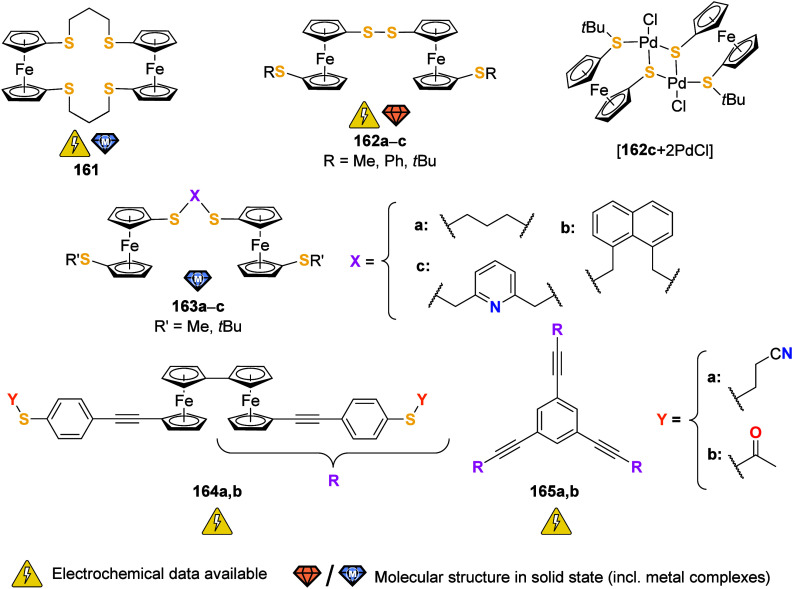
Thioethers with two or more ferrocenyl or ferrocenylene groups in their backbone or periphery. [**162c**+2PdCl] refers to the reaction product of **162c** and [Pd(PhCN)_2_Cl_2_].^367^

**Figure 28:**
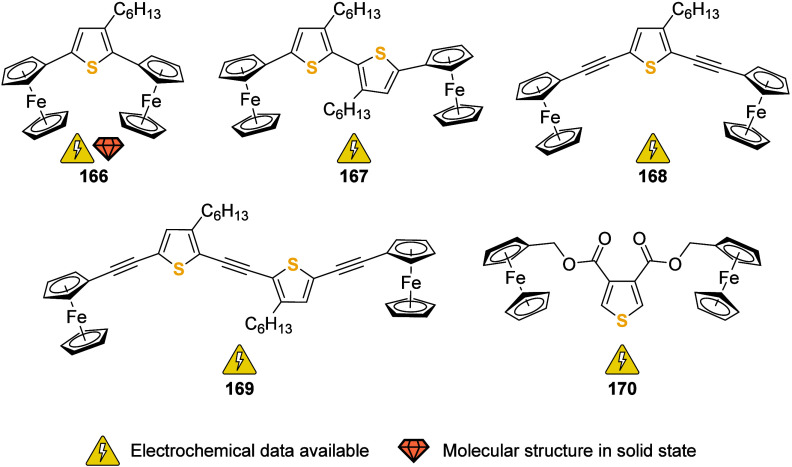
Thienylene-based ligands with ferrocenyl termini.

Moving on to sulfur-based systems, an overview of thienyl derivatives, thioethers, disulfanes, tetra-sulfur ligands, and thiophene dicarboxylato ligands is given in Figure 27 and Figure 28.

The tetrathiamacrocycle **161** (Figure 27), a first representative for the thioether compounds, with two flanking 1,1′-ferrocenylene groups (which could also be considered a [5.5]ferrocenophane) was found to coordinate copper(I) and silver(I) as well as platinum(II) and palladium(II) in a tetradentate mode as evidenced by X-ray crystallography. While the two coinage metal complexes could be oxidized reversibly, the platinum group metal complexes did only display irreversible oxidation events.^368^ Bis(1′-organylthiolatoferrocenyl)-disulfanes **162a**–**c** have been prepared from the reaction of 1,2,3-trithia-[3]-ferrocenophane with the corresponding organolithium reagent (RLi, R = Me, Ph, and *t*Bu). Compounds **162a**–**c** were expected to have the potential to bind to metal centers in a variety of ways. Unexpectedly, the reaction of **162c** with [PdCl_2_(PhCN)_2_] gave a small amount of a palladium(II) complex ([**162c**+2PdCl] in Figure 27) that was formed by an unusual insertion reaction into the disulfide bridge. The complex is a dimer with a planar central Pd_2_S_2_ ring where the monomeric moiety consists of a PdCl fragment chelated by the S*t*Bu and the sulfido group of one ferrocenylene group. Bis(1′-organyl-thiolatoferrocenyl)-disulfanes **162a,c** exhibited two separated one-electron oxidations in CH_2_Cl_2_ solution with 0.2 M (TBA)[PF_6_] as supporting electrolyte. The monocation [**162c**]^+^ was quite stable, whereas the dication [**162c**]^2+^ slowly decomposed.^367^ This work was followed up by the same group, and the initially obtained bis(1′-organylthiolatoferrocenyl)-disulfanes **162a,c** were converted to open-chain sterically hindered tetra-sulfur ligands **163a**–**c** by successfully cleaving the disulfanes with lithium triethylborohydride followed by reaction with dibromo-organyl species (Br–X–Br, for X see Figure 28). Here, both types of ligands, the disulfanes **162a,c** and the tetra-sulfur ligands **163a**–**c**, were reacted with [Cu(MeCN)_4_][PF_6_] and [PdCl_2_(cod)]. The resulting dinuclear (with respect to the coordinated metals) complexes of copper(I) and palladium(II) have been fully characterized. The structurally characterized copper(I) complexes of **162c** (R = *t*Bu) and **163b** (R = *t*Bu), as well as the PdCl_2_ complex of **163b** (R = *t*Bu) showed a bis-bidentate chelating mode by the terminal SR group as well as the bridging sulfur atoms of the disulfane of the dithioethers (*i*.*e*., the two sulfur atoms on each ferrocenylene unit coordinating one metal), thus holding the metals in close proximity by the symmetric double bidentate binding mode.^369^ Unfortunately, no electrochemistry for the **163** family seems to have been reported.

Bennett and Long, as previously mentioned in the discussion of **151**, have also prepared potential S donors.^356^ Both biferrocene derivatives **164a,b** and *C*_3_-symmetric 1,3,5-trisubstituted **165a,b** come as 2-cyanoethyl thioethers (**164a** and **165a**) and as thioacetates (**164b** and **165b**). Their respective, highly modular, preparations involve Sonogashira-type *C*,*C* coupling reactions of iodinated (bi)ferrocenes to install the (cyanoethylthio)ethynylphenylene spacers as well as to couple the resulting 1,1′-unsymmetric ferrocenes to the desired scaffold (*e.g.*, 1,3,5-triethynylbenzene for **165a**). Treatment of the thioethers, potential S donors in their own right, with strong base (NaOMe in MeOH) followed by reaction with acetic anhydride affords the thioacetates. No coordination studies have been undertaken, but thioacetates can be used to bind gold surfaces,^370^ to act as O donors for harder metals,^355^ or as S donors toward softer metals.^371^ For all four derivatives **164a,b** and **165a,b**, data from cyclic voltammetry (CH_2_Cl_2_, 0.2 M (TBA)[PF_6_], 50–600 mV/s) is available, showcasing highly reversible redox-behavior. Biferrocene derivatives **164a,b** show two separate oxidations, while all three ferrocenylene groups of **165a,b** are oxidized at the same potential under these conditions.^356^

Patra and Roy have reported the synthesis of wire-like molecules with ferrocenyl moieties, conjugated with one or two thienylene (**166** and **167**) and thienylene-ethynyl (**168** and **169**) groups (Figure 28). Compounds **166** and **167** were synthesized through Negishi cross-coupling reaction from the dibromo derivatives of the respective thienyl oligomers (2,5-dibromo-3-hexylthiophene for **166**, and 5,5′-dibromo-3,3′-dihexyl-2,2′-bithiophene for **167**) and ferrocenylzinc chloride in the presence of [Pd(PPh_3_)_4_] in good yields. For the preparation of compounds **168** or **169**, a Sonogashira cross-coupling reaction (Pd^II^/Cu^I^ catalyst system, Et_3_N) between ethynylferrocene and the diiodo precursors of the respective thienylene-ethynyl oligomer was employed. The electrochemical studies of **168** and **169** in CH_2_Cl_2_ solution with 0.2 M (TBA)[PF_6_] as supporting electrolyte showed that the two Fe^II^ centers of the ferrocene units were oxidized simultaneously, suggesting the absence of electronic communication between the ferrocenyl moieties. However, for the derivatives with thienylene spacers (**166** and **167**), the cyclic voltammograms showed two well-shaped oxidation waves under the same conditions, suggesting electronic communication between the two ferrocene moieties. Potential applications include the use as organometallic molecular wires, with various π-conjugated spacers and redox-active metal termini to access efficient NIR-absorbing materials.^372^

Diferrocenyl-3,4-thiophene dicarboxylate (**170**) was prepared by Ghazzy et al. from 3,4-dicarboxylic acid dichloride thiophene and two equivalents of lithium ferrocenylmethoxide (FcCH_2_OLi). The cyclic voltammogram of **170** exhibits a reversible ferrocene-related redox couple using (TBA)[B(C_6_F_5_)_4_] as the supporting electrolyte.^373^ As mentioned earlier, multiply ferrocenylated thiophenes were also prepared and extensively studied for their electrochemical behavior by Lang and co-workers.^128^

**Figure 29:**
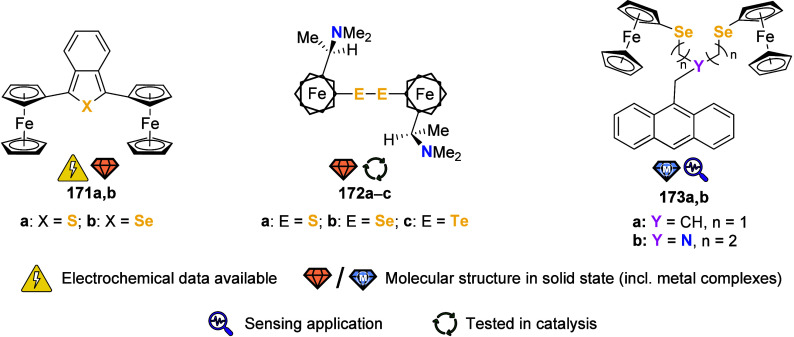
Sulfur- and a few selected selenium- and tellurium-based ligands with two peripheral ferrocenyl groups in their backbone or as substituents.

Benzothiophene and its selenium analogue were employed as spacers between two ferrocenyl groups (Figure 29). 1,3-Bis(ferrocenyl)benzothiophene (**171a**) and 1,3-bis(ferrocenyl)benzoselenophene (**171b**) have been prepared following a multistep synthesis. Starting from phthaloyl dichloride, 1,2-di[*S*-(2-pyrimidyl)]benzenedithioate was prepared, which was then reacted with ferrocenyllithium to synthesize the *ortho*-bis(ferrocenyl)benzene core. Ring closure with Lawesson’s reagent (for **171a**) or bis(dimethylaluminum)selenide as selenating reagent (for **171b**) yielded the desired compound in good yields. The electrochemical studies of the neutral organic–organometallic hybrid molecules **171a,b** in THF or dichloromethane with 0.1 M (TBA)[PF_6_] as supporting electrolyte showed reversible multielectron transfer assigned to the chalcogenophene (organic) and ferrocene (organometallic) fragments owing to good stability of the negatively charged reduction products (radical anions) and positively charged oxidation products (mono- and bis-ferrocenium cations).^374,375^

Aiming for new chiral ligands for transition metal-catalyzed asymmetric hydrosilylation, Nishibayashi et al. have reported the synthesis of a wide number of chiral diferrocenyl dichalcogenide derivatives (three of them are shown in Figure 29, **172a**–**c**). The ligands are planar chiral due to the 1,2-unsymmetrically disubstituted ferrocene moiety and additionally feature *C*-chiral 2-(dimethylamino)ethyl substituents. Their use as ligands in Rh^I^-, Ir^I^-, and Ru^II^-catalyzed asymmetric hydrosilylation afforded up to 88% enantiomeric excess (ee).^376,377^ Their inclusion in this section is debatable, as the chalcogenides are expected to merely act as linkers while the metal will most likely be coordinated by the NMe_2_ groups, but given the scarcity of such systems, we decided to include them in this section.

Liu et al. have reported the synthesis and characterization of anthracene-based ferrocenylselenoethers, namely 1,3-bis(ferrocenylseleno)-2-(anthracen-9′-ylmethyl)propane (**173a**, Y = CH_2_, *n* = 1) and *N,N*-bis[2-(ferrocenylselena)ethyl]-*N*-(anthracen-9′-ylmethyl)amine (**173b**, Y = N, *n* = 2) for multichannel ion sensing. Compounds **173a,b** were prepared by reducing diferrocenyl diselenide (Fc_2_Se_2_) and reacting the selenide with the corresponding dibrominated anthracene derivatives. Due to the joint presence of a redox moiety (ferrocenyl) and a fluorescent chromogenic group (anthracenyl) **173a,b** react as “switch-on” fluorophores, depending on the to-be-detected cation. While **173a** responded to the presence of Cu^2+^ and Hg^2+^, **173b** was selective for Cu^2+^, Zn^2+^, and Hg^2+^. The authors speculated Cu^2+^ to induce the oxidation of the ferrocene unit in both **173a,b**, but only the N mixed-donor sensor **173b** showed a concomitant fluorescence enhancement. The coordination of Zn^2+^ with the N donor in **173b** led to fluorescence enhancement, while the heavy metal effect of Hg^2+^ caused fluorescence quenching of **173a,b**. The voltammograms of **173a,b** in dry CH_3_CN/CH_2_Cl_2_ (1:1, v/v) solution containing 0.1 M (TBA)[PF_6_] as supporting electrolyte showed a reversible one-electron redox couple due to the ferrocene/ferrocenium oxidation process and an irreversible oxidation peak for the anthracene unit. Addition of M^2+^ (M = Cu, Zn, Hg) led to observable positive shifts of the redox potentials of the ferrocenyl and anthracenyl groups. A copper(I) complex [Cu_4_I_4_(**173b**)_2_]_∞_ has also been prepared and structurally characterized, showing a distorted cubane-like Cu_4_I_4_ core which is double-bridging and linked by two ligands via the two Se atoms to produce a 1D loop chain.^378^

**Scheme 5:**
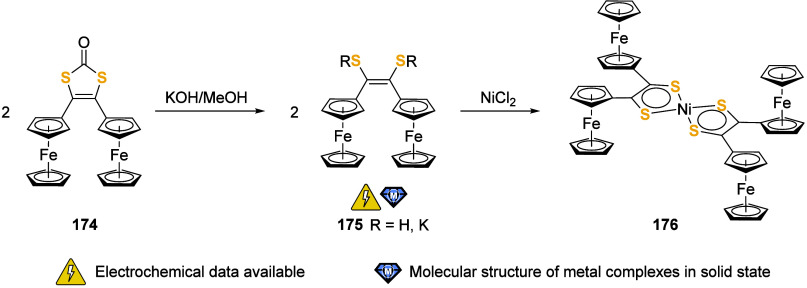
General Scheme for the Formation of the Nickel(II) Bis(dithiolene) Complexes Exemplified for **176**

Very important derivatives in the group of sulfur-based ferrocenyl ligands are the dithiolene derivatives (**175**) and the metal complexes of the corresponding substituted ethylenedithiolate dianions. The square-planar transition metal dithiolene complexes, especially those of Ni, are of interest as near-IR dyes. Even the unsubstituted parent Ni dithiolene complexes show a transition at the near-IR edge of the visible spectrum and the dithiolene absorption can be shifted by proper substitution. The simplest derivative, bis(ferrocenyl)dithiolene **175** (R = H), was synthesized from 3,4-diferrocenyl-1,3-dithiol-2-one (**174**) and KOH in methanol and can act as a bidentate ligand after deprotonation (Scheme 5). The ethylenedithiolate dianion was reacted with nickel dichloride in diluted HCl to give the corresponding nickel bis(dithiolene) complex **176** in good yield after air oxidation.^379,380^ Complex **176** shows an interesting electronic structure.^380^ It is diamagnetic and can be oxidized (ferrocenyl groups) and reduced (dithiolene ligands); dithiolene ligands are known to be redox-noninnocent, thus complicating the assignment of an oxidation number for nickel. Dithiolene complex **176** has been used to study the effects of solvents and supporting electrolytes (SEs) on the electrochemical behavior of multiredox active complexes by Geiger, Barrière and co-workers.^381^ Displaying four separate ferrocenyl-located oxidations and two dithiolene-associated reductions, it proved to be an ideal model system. The authors systematically varied both solvents and SEs, changing the latter from smaller anions (Cl^–^, [PF_6_]^−^) to larger weakly coordination anions (WCAs; [B(C_6_F_5_)_4_]^−^, [B(3,5-(CF_3_)_2_C_6_H_3_)_4_]^−^), showing that the difference between the individual redox events strongly depends on the choice of medium. This in turn allowed to discriminate between purely electrostatic factors (*i*.*e*., the oxidation becoming more difficult because of Coulombic repulsion) and stabilizing factors due to electronic interaction. These systematic studies thus allowed to experimentally determine the corresponding contributions^69^ and revealed the different extent to which Robin–Day Class II and III systems are influenced by medium effects: while the strongly electronically coupled Class III systems are hardly influenced by changes in the medium,^382^ such alterations strongly act on lesser interacting Class II systems as corroborated by UV/vis–NIR spectroscopy and electrochemistry (and, of course, spectroelectrochemistry).^383,384^

**Figure 30:**
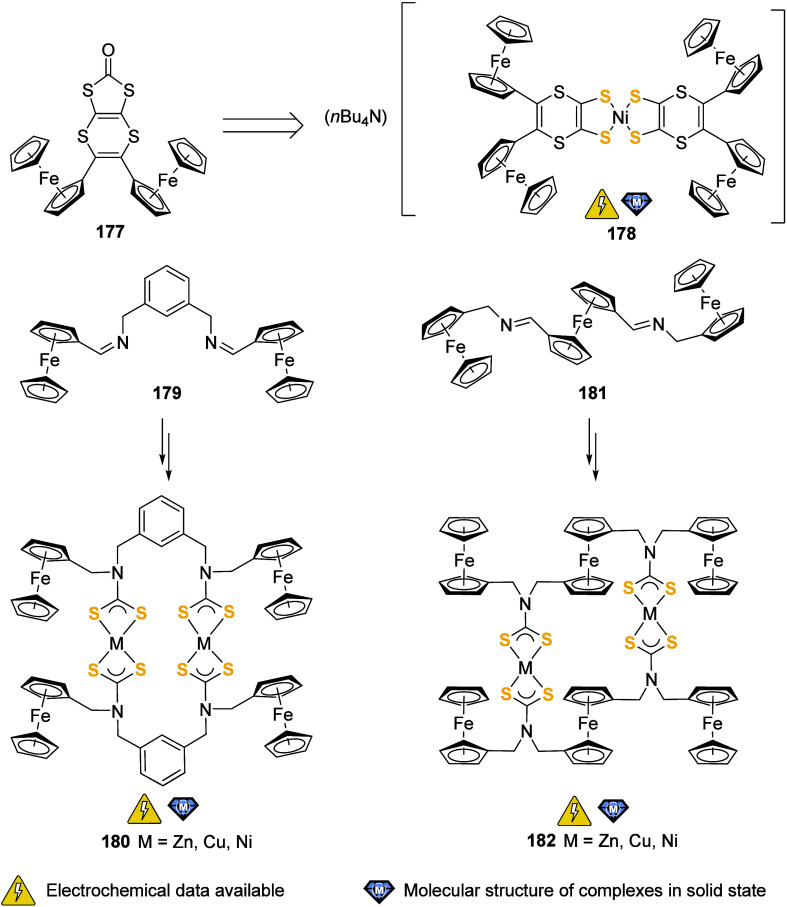
Dithiolene ferrocenyl complexes.

Following almost the same protocol as presented in Scheme 5, Noh and Lee have reported the synthesis and properties of the tetrakis(ferrocenyl) nickel-bis(1,4-dithiin-5,6-dithiolate) complex (**178**, Figure 30).^385^ For this, 1,2-bis(ferrocenyl)ethylene was synthesized by McMurry coupling using ferrocenecarboxaldehyde and mixture of titanium tetrachloride and zinc as catalyst. In the next step, a Diels–Alder type [2+4] cycloaddition reaction of oligomeric 1,3-dithiol-2,4,5-trithione with 1,2-diferrocenylethylene gave an intermediate that was successively treated with DDQ (2,3-dichloro-5,6-dicyanobenzo-1,4-quinone) to yield the ferrocenyldithiolene ligand precursor. Then, the oxo derivative (ligand **177**, Figure 30) was prepared by treatment with Hg(OAc)_2_, and the reaction of **177** with KOH in ethanol generated the dithiolate ligand. The addition of NiCl_2_ and (TBA)Br in ethanol and concomitant exposure of the reaction mixture to air yielded the desired complex nickel-bis(2,3-diferrocenyl-1,4-dithiin-5,6-dithiolate) as tetra-*n*-butylammonium salt (**178**) in good yield. The molecular structure of the nickelate complex anion showed a boat-like conformation with the four ferrocenyl groups folding up. The cyclic voltammogram of **178** in THF with (TBA)[BF_4_] as the supporting electrolyte (0.1 M) showed two reversible redox peaks associated with the nickel(II) dithiolate complex and one quasi-reversible peak associated with the ferrocenyl moieties. The complex exhibits a strong near-IR absorption, suggesting its possible use as a near-IR dye for absorbing lower energy.^385^

Dithiocarbamate-containing ligands are accessible from primary amines and CS_2_.^386^ Bis-imines **179** and **181** (Figure 30) were first prepared from condensation of ferrocenecarboxaldehydes with the corresponding amine. Reduction with sodium borohydride yielded secondary ferrocenyl amines which were further reacted with carbon disulfide, potassium hydroxide and transition metal (zinc, copper, and nickel) acetate to yield the corresponding zinc(II), copper(II) and nickel(II) complexes (**180** and **182**) in good yields. The bis-zinc xylyl-bridged dithiocarbamate dimer (**180**, M = Zn), containing four redox-active ferrocenyl groups on the macrocycle periphery, exhibited a single reversible oxidation wave in CH_2_Cl_2_/CH_3_CN (4:1) solution with 0.2 M (TBA)[BF_4_] as supporting electrolyte, which suggests that all four ferrocene moieties are oxidized in a single step and are electrochemically independent of one another. The bis-copper(II) analogue (**180**, M = Cu) displayed a broad oxidation redox wave, suggesting that the copper(II)/copper(III) dithiocarbamate redox couple overlaps with the respective ferrocene oxidation couple. For the bis-nickel(II) macrocycle (**180**, M = Ni), two oxidation couples are observed, a reversible wave which corresponds to the ferrocene redox couple, and an irreversible oxidation at assignable to the known Ni^II^/Ni^IV^ dithiocarbamate oxidation process. The zinc(II) macrocycle (**182**, M = Zn) containing six ferrocenyl groups displayed a more complicated electrochemical behavior, where two oxidation waves, but only one reduction wave was observed.^387^

### 2.4 C Donor Ligands

**Figure 31:**
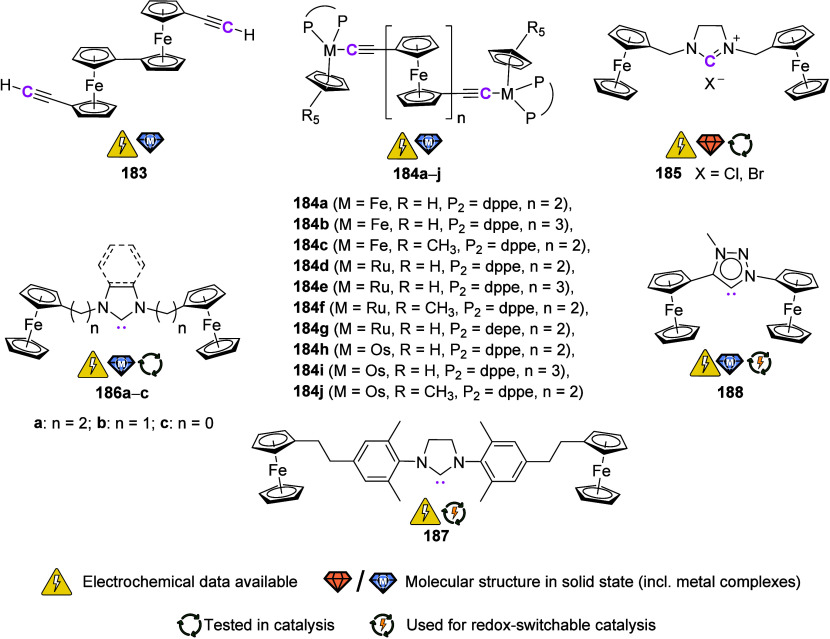
Carbon-based ligands (and ligand precursors in case of **183**) with two ferrocenyl or ferrocenylene groups in their backbone or as peripheral substituents.

Oligoferrocenyl-substituted carbon-based ligands mainly comprise acetylides and carbenes. Maybe not the first example to come to mind, ethynyl-substituted biferrocene **183** (Figure 31), first prepared by Long and co-workers in 1995, has found many applications as a bis-acetylide ligand after deprotonation.^388^ Initially designed aiming at applications in NLO, the Long group first prepared symmetric ruthenium(II) ({RuCl(dppm)}, dppm = 1,2-bis(diphenylphosphanyl)methane) and manganese(I) ({Mn(CO)_3_(dppm)}) complexes of deprotonated **183** (see Figure 31 for details) which showed promising electronic communication between the biferrocene unit and the terminal metal complex fragments upon oxidation. Reacting **183** with suitable platinum(II) complex precursors, *e.g.*, *cis*-[PtCl_2_(PR_3_)_2_] (R = *n*Bu and Me) yielded a reversibly oxidizable dinuclear metallamacrocycle.^389^ In joint efforts, the groups of Lang and Lapinte have used the deprotonated **183** to symmetrically bridge metal complex fragments of ruthenium(II) ({Ru(η^5^-C_5_H_5_)(PPh_3_)_2_} and {Ru(η^5^-C_5_H_5_)(dppf)}, dppf = 1,1′-bis(diphenylphosphanyl)ferrocene),^388^ osmium(II) ({Os(η^5^-C_5_H_5_)(PPh_3_)_2_}), gold(I) ({Au(PPh_3_)}), and iron(II)^390,391^ (Figure 31) which, particularly in the case of the tetranuclear iron complexes [1′,1‴-(Cp*)(η^2^-dppe)Fe–C≡C)_2_bfc] (bfc = biferrocenediyl), allowed for a stepwise 4-fold oxidation (in dichloromethane with 0.1 M (TBA)[PF_6_] as supporting electrolyte) including the isolation of the mixed-valent species. The combination of acetylide and ferrocenyl termini has also found other forms, for example a 1,3,5-trisubstituted benzene with two ferrocenyl termini used for the coordination of a ruthenium-containing fragment (not shown).^392^ Even though not structurally characterized, the bis-ethynyl motif has also been extended to terferrocenes by the group of Dong (**184**, Figure 31).^84,393^ Aiming to micromodulate the electronic coupling between the two terminal metal centers, the end-capped metal centers and the connecting spacers were varied. The magnitude of the electronic coupling between the two terminal metal centers in this series of complexes was derived electrochemically. These complexes underwent sequential reversible oxidation events in anhydrous CH_2_Cl_2_ solution with 0.1 M (TBA)[PF_6_] as supporting electrolyte; the low-potential waves have been assigned to the two end-capped metal centers.

Given the great success of *N*-heterocyclic (and, more recently, also acyclic) carbenes in coordination chemistry and catalysis, it is hardly surprising that ferrocene would appear on the scene sooner or later. Reviews by Bildstein,^394^ Siemeling,^395^ and Yoshida^102^ may serve as entry points to the fruitful combination of carbene chemistry and ferrocenes up until 2018. As for multiferrocenyl derivatives, only a relatively small number has been reported. *N,N′*-Bis(ferrocenylmethyl) imidazolium salts (**185**) have been reported by Özbek et al.; their synthesis involved the reaction between *N,N′-*bis(ferrocenylmethyl)ethylenediamine with the corresponding NH_4_X (X = Cl or Br) salts. Cyclic voltammetry of **185** in dichloromethane with 0.1 M (TBA)[PF_6_] as supporting electrolyte showed only one reversible ferrocenyl-centered wave for both compounds, regardless of the halide ion.^396^ The *in situ* prepared mixture Pd(OAc)_2_/1,3-bis(ferrocenylmethyl)imidazolium chloride (**185**, X = Cl) has shown very good catalytic properties for Suzuki cross-coupling reactions of several aryl bromides with phenylboronic acid under optimal conditions.^397^

From our survey, Bildstein, and co-workers were the first to decorate an Arduengo-type carbene with pendant ferrocenyl substituents (**186a** (only benzannulated, dashed lines) and **186b** (with and without benzannulation)), and in turn employed them in the preparation of a range of redox-active complexes with tungsten(0) ([W(CO)_5_], **186a,b**), palladium(II) (PdI_2_, 2 × **186b**), and mercury(II) (HgI_2_ (2 × **186b**) or HgBr_2_ (2 × **186a**)).^398^ The direct attachment of the ferrocenyl group at the nitrogen atoms (**134c**, *n* = 0) was achieved a little later in 1999, but the free carbenes (**186c**) could not be synthesized from the *N*-heterocyclic nucleophilic carbene precursors. However, the corresponding silver(I) complex [Ag(**186c**)_2_][B(C_6_H_5_)_4_] could be obtained from the reaction of the corresponding of *N*,*N*′-diferrocenylimidazolium salt with Ag_2_O and fully characterized.^399^ Derivatives of **186c** have since been used for the coordination of palladium(II) ({PdI_2_L}, L = py, PPh_3_)),^400^ iridium(I) ({Ir(cod)Cl}, {Ir(CO)_2_Cl}), and group 6 metals ({M(CO)_5_}, M = Cr, Mo, W).^401^ Rhodium(I) ({Rh(cod)Cl}) as well as iridium(I) ({Ir(cod)Cl}) complexes of *C*-chiral analogues of **186b** with an CHMe bridge instead of CH_2_ have been tested in catalytic asymmetric transfer hydrogenation, however only little enantiomeric excess was observed.^402^

Süssner and Plenio reported carbene **187**, which was used as a ligand for ruthenium(II) in a Grubbs–Hoveyda olefin metathesis (pre)catalyst, to effect precipitation of the catalyst upon oxidation after the catalytic conversion had been finished.^403^ By this, they provided one of the first examples for the now burgeoning field of redox-switchable catalysis (RSC, see section 3.2, Figure 33). In a different approach of RSC, the Sarkar group employed mesoionic (sometimes also termed “abnormal”) carbene **188** (and other monoferrocenyl derivatives), first prepared by Molina and co-workers from a copper-catalyzed Huisgen cycloaddition of ferrocenylacetylene and ferrocenyl azide.^316^ By double oxidation, the electron density on a coordinated chloridogold(I) fragment was modulated, in turn altering the catalytic activity.^404^ Naturally, electrochemical data for the free carbenes is not available, and it is usually the respective metal complexes that are characterized by electrochemical methods regarding their redox profiles. In some cases (*e.g.*, for **188**), the respective carbene precursor salts have been electrochemically profiled.^316^

## 3 On, Off, and Everything in Between: Ferrocene Ligands for Redox-Switchable Catalysis

As the last two examples have already demonstrated, the integration of ferrocenyl groups into existing or novel ligand frameworks can equip those systems with added, exploitable redox functionality. While a few instances of redox-switchable applications have already been briefly mentioned in passing, the following subsection is exclusively concerned with how ferrocene has (mostly) successfully been used to change chosen properties on demand and presents selected examples. Redox-switchable catalyst systems will comprise the second half of the subsection and will be preceded by examples of redox switches in other contexts. A third, related possibility, namely using ferrocenyl groups as a read-out for sensing applications (usually through measurable alterations in their redox potentials), is only mentioned here, and will not be described in more detail.^405^ However, some examples have been included in the previous chapters.

### 3.1 Multiferrocene-Derived Redox Switches

The most obvious change that results from oxidizing ferrocene is the creation of a positively charged moiety. Thus, when an already charged molecule is subjected to a ferrocene-centered oxidation, the overall charge can be canceled or increased.

**Figure 32:**
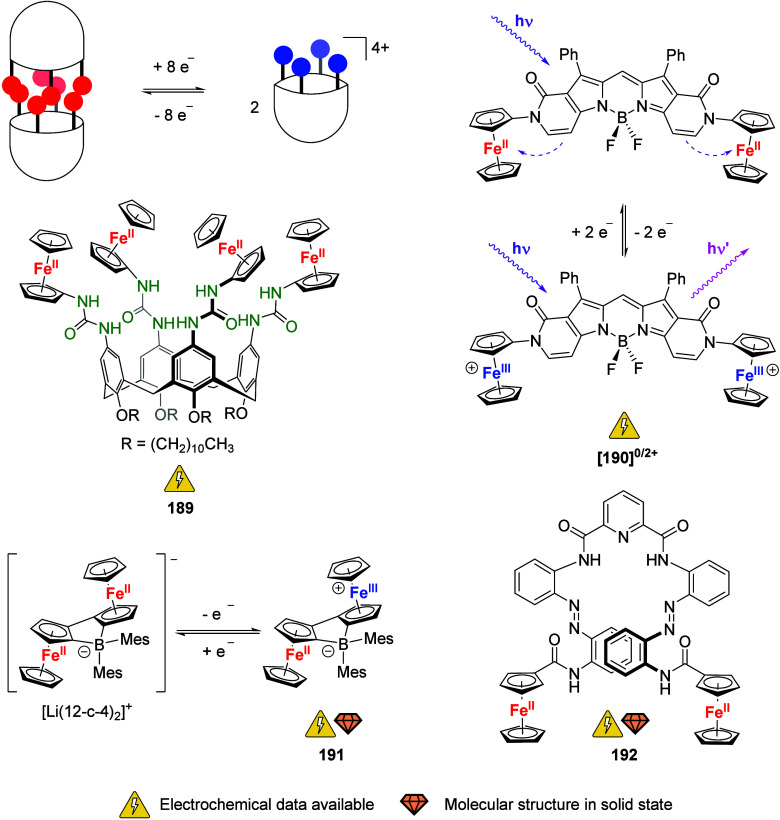
Ferrocene-based redox switches (dashed arrows represent the quenching of excited states, wavy arrows signify light; 12-c-4 = 12-crown-4).

Creating several cationic ferrocenium sites at once can furthermore induce structural changes due to Coulombic repulsion. Moon and Kaifer applied this concept to tetra-ferrocenylated calix[4]arene **189** (for calix[*n*]arenes, the *n* in brackets denotes the number of repeating phenol units). In its native uncharged state, **189** forms a dimeric nanocapsule through hydrogen bonding between the four urea units (green in Figure 32) as evident from NMR-spectroscopic investigations.^406^ Upon (reversible) full oxidation, the hydrogen bonds are broken and the capsule dissociates into its two monomeric constituents, signified by a 2-fold increase in the dissociation constant measured by NMR spectroscopy. The accumulation of positive charge has furthermore been transferred to ferrocene-containing polymers (not shown) in a demonstration by Gallei and co-workers.^407^ A methacrylate polymer featuring ferrocenyl-substituted side chains, grafted onto a silicon surface, could be oxidized and, in its oxidized form, proved more wettable as signified through a strongly decreased contact angle between a drop of water and the functionalized surface.

Next to the mere creation and/or extinction of charge, ferrocene and ferrocenium also differ in their behavior as quenchers for luminescence. While ferrocenyl groups are well-known to, in general, quench excited states by either energy or even electron transfer, this ability is generally significantly attenuated for ferrocenium species.^408^ Appending potential dyes with switchable luminescence quenchers is thus an interesting strategy to control emission properties at will, for example in the oxidizing environments of certain cell compartments.^409^ Doubly ferrocenylated BODIPY (boron-substituted dipyrromethene) dye **190**, while not yet fit for biological applications, is a good example in this context.^410^ While not the first or only BODIPY derivative with ferrocenyl groups,^411,412^**190** is nonemissive in its native state since ferrocene quenches the corresponding excited states (signified by dashed lilac arrows in Figure 32). During electrochemical oxidation, the intense fluorescence typical for BODIPY derivatives^413^ is restored, making **190** an effective redox-switchable multiferrocenyl fluorophore. Similar, porphyrin-based redox switches have also been reported.^414^

Employing the charge generation upon oxidation of a ferrocenyl(ene) group, Jäkle and team introduced zwitterionic **191** as a redox-switchable chiral anion through the reduced iron(II,II) form of the bridged biferrocene backbone.^415^ The reversible redox shuttling between neutral and anionic form could be of interest in the context of asymmetric catalysis and for the stereoresolution of racemic mixtures. Using the same synthetic methodology based on Kagan’s sulfoxide, the authors also reported a tin analogue of **191** that becomes cationic upon oxidation. Ferrocenyl groups have also been used to create redox-active foldamers, that is, molecules adopting helical conformations reminiscent of naturally occurring, self-folding systems. Complementing earlier work by the groups of Heinze^416,417^ and Santi,^418^ who had already investigated the use of redox-active ferrocenyl termini and ferrocenylene linkers toward redox-switchable foldamers, Pike and co-workers designed the multistimuli-responsive system **192** (Figure 32).^419,420^ Foldamer **192** combines photoswitchable azobenzene groups (reversibly enabling the switch between stable folded and random-coil conformations) with ferrocenylamide termini. As both ferrocenyl groups can be oxidized at the same potential, as determined by cyclic voltammetry (0.1 M (TBA)[PF_6_] in CH_2_Cl_2_, 100 mV/s), the conformation can be controlled by either light or redox agents. While **192** has not yet been explored in coordination of metals, the option to use this responsive system as a smart pincer-type ligand could prove fruitful also in the context of redox-switchable catalysis.

**Scheme 6:**
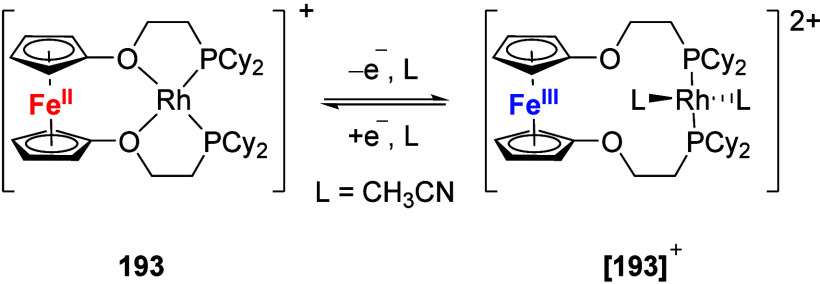
Redox-Hemilabile Ferrocene-Derived Ligand by Mirkin and Co-workers

Building on the earlier work by Jeffrey and Rauchfuss who coined the original hemilability concept for ligands combining both a strong, tightly bound and a weaker, more loosely bound donor moiety,^421^ the Mirkin group exemplarily used rhodium complex **193** (Scheme 6) to demonstrate this effect. Through a combination of cyclic voltammetry and NMR spectroscopic studies they were able to show a change of coordination mode of a phosphane ether ligand which they mainly attributed to an increased electrostatic repulsion between the two cationic metal centers. A closely related ligand, namely a dithiolene-based ferrocene ligand (not shown), was used to demonstrate the effect of redox-induced hemilability on catalytic efficiency.^379^ The field of redox-switchable catalysis (RSC) was born.^422^

### 3.2 Redox-Switchable Catalysis

**Figure 33:**
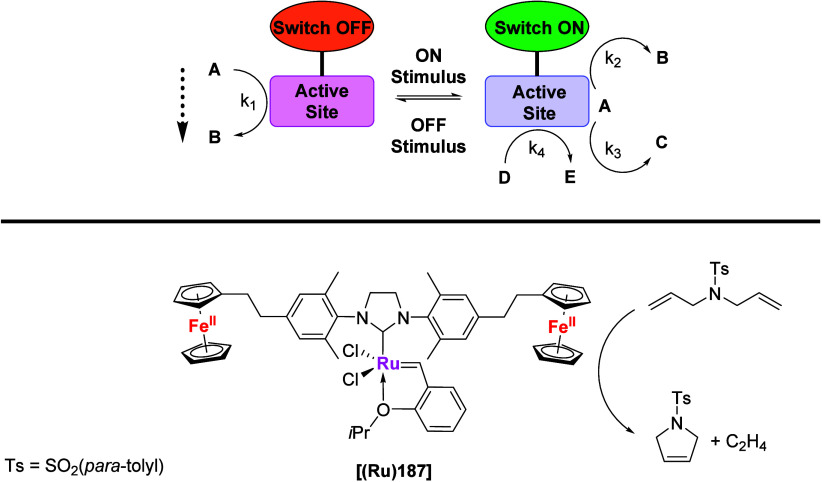
General scheme of stimuli-responsive catalysis (top) and a prominent early example of redox-switchable catalysis (bottom).^403^

Redox-switchable catalysis, concisely defined by Bielawski as using “redox active ligands to influence the catalytic activities displayed by coordinated metals”,^423^ falls in the greater research area of stimuli-responsive or artificially switchable catalysis.^424,425^ Inspired by the endlessly complex and highly regulated enzymatic transformations employed by nature,^426^ chemists seek to follow suit and use external stimuli to temporally and sometimes even spatially control the reactivity of selected catalytic reactions. A general scheme for this is depicted in Figure 33 (top).

In its most basic iteration, the native form of a catalyst (Switch OFF) might already be active in a selected conversion of A to B with a rate constant *k*_1_ (or even catalytically silent). After applying the selected stimulus and a corresponding change in the switchable moiety of the system (Switch ON), the active site is modified in a way so that a change in activity (*k*_2_ ≠ *k*_1_) ensues (for example, inducing a reaction to take place or stopping the originally catalyzed reaction altogether).

This change can either be an altered electronic configuration–and indeed, a metal center itself can be the subject of, for example, a change in oxidation state^427^ making it switch and active site in one, or a change in accessibility of the active site. Not only can the rate of the original transformation of A to B now be changed (both positively and negatively), the catalyst could also start to convert substrate A to a different product C (with a rate constant *k*_3_ > *k*_2_), a feature highly sought after in asymmetric catalysis where B and C might represent enantiomers.^428^ Finally, the switched-on system might display an altered substrate preference of D over A (*k*_4_ > *k*_2_, *k*_1_), generally termed orthogonal reactivity.

In a distinction between stimuli-responsive and stimuli-switchable, a truly switchable system must be able to return to its native state by an appropriate OFF stimulus, and, ideally, that process should be repeatable for many cycles. Signified by the dashed arrow in Figure 33 (top), the catalyst system might also change its solubility with respect to its current state. As far as appropriate stimuli are concerned, the integration of photo-,^429−431^ pH-,^432^ iono-,^433^ and even mechano-responsive^434^ and mostly -switchable units into catalysts has been successfully accomplished (the exception for reversibility being the mechanically activated system). However, from the start of this direction of research, redox-responsive and -switchable systems have so far proven the most versatile and fruitful candidates. From an industrial perspective, the possibility to immobilize corresponding catalysts on electrodes and/or use electrodes for a traceless redox process (in contrast to employing stoichiometric amounts of oxidants and reductants) is particularly promising.^435^

The application of a cobaltocene-derived ligand by Wrighton and co-workers, in the form of its rhodium complex [Co(η^5^-C_5_H_4_PPh_2_)_2_Rh(acetone)_n_][PF_6_], is generally claimed to be the first reported example of a redox-switched catalytic transformation.^436^ In its reduced form (Co^II^), the complex proved to be an efficient catalyst for the hydrogenation of, for example, cyclohexene, while its oxidized form (Co^III^) was a more efficient catalyst for the hydrosilylation of acetone, already demonstrating orthogonal reactivity. Switching between the states could easily be accomplished by the *in situ* addition of suitable oxidants (decamethylferrocenium hexafluorophosphate) and reductant (cobaltocene). From our survey, the first use of ferrocenyl groups for a similar purpose was reported ten years later by Süssner and Plenio with the ferrocenylated Grubbs–Hoveyda-type olefin metathesis catalyst **[(Ru)187]** (Figure 33; for the free ligand, see Figure 31).^403^ Next to the successful and repeatable OFF-switching of **[(Ru)135]** by *in situ* oxidation of the ferrocenyl groups with acetylferrocenium triflate, oxidizing the catalyst did also precipitate it, allowing for an easy recovery and thus recyclability. Simple reduction with octamethylferrocene regenerated and resolubilized the native species which could then be used for a follow-up run in the ring-closing metathesis of *N*-tosyldiallylamine. A more detailed study into the molecular level of switching, as well as extending the use of this kind of ligands to bind iridium, was subsequently conducted by Bielawski and co-workers.^423^ For a ruthenium complex of a monoferrocenyl carbene (not shown) with a less remotely placed ferrocenyl substituent, they were able to show that the oxidation of the ferrocenyl substituent did induce a reduction in electron density by combining (spectro)electrochemical measurements, ^57^Fe Mössbauer and EPR spectroscopy. In turn, this reduced electron density led to diminished catalytic activity. Placing a single 1,2-ferrocenylene group into the backbone of the Schrock carbene ligand was found to be a less generalizable strategy, yet did allow for an oxidation-induced ON (rather than OFF) switch, demonstrating that the directionality of the switch could be altered by careful ligand design, too.^437^

**Figure 34:**
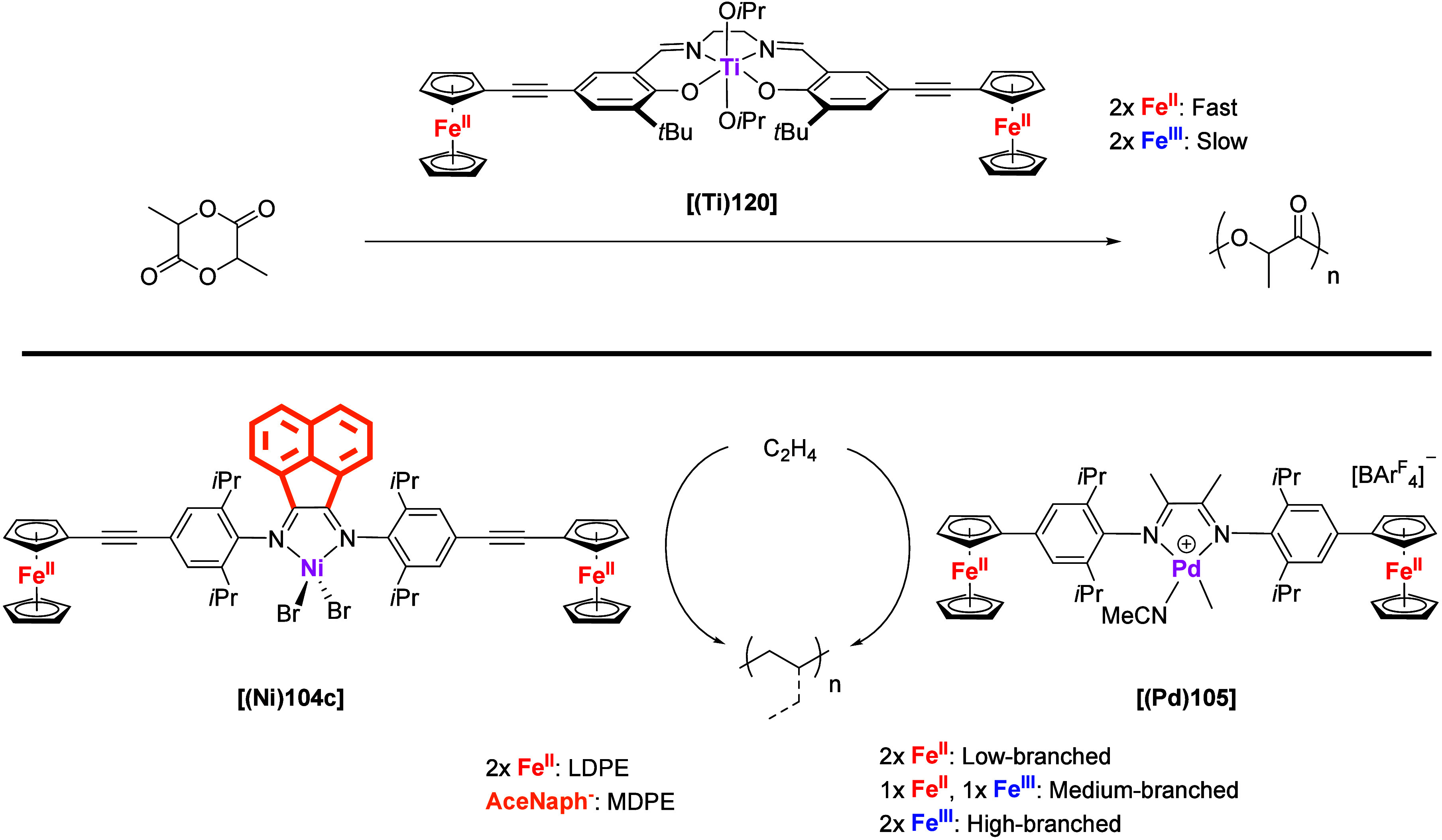
Redox-switchable catalyst systems used in the polymerization of *rac*-lactide (top) or for the polymerization of ethylene (bottom; AceNaph = acenaphthenylene; BAr^F^_4_ = [B(3,5-(CF_3_)_2_C_6_H_3_)_4_]^−^; LDPE = low-density polyethylene; MDPE = medium-density polyethylene).

Next to olefin metathesis, polymerization reactions have proven to be a suitable playground for RSC.^438,439^ After the breakthrough of Plenio, the group of Gibson, Long, and co-workers reported the use of salen-type ligand **120** (see also Figure 19) with appended ferrocenyl groups in the titanium(IV)-catalyzed ring-opening polymerization (ROP) of *rac*-lactide.^285^ Reversible *in situ* switching could be carried out with silver(I) triflate as the oxidant and decamethylferrocene as the reductant, and, similar to **[(Ru)187]**, **[(Ti)120]** (Figure 34, top), was more active in its reduced than in its fully oxidized form even though the ferrocenyl groups were placed quite remotely from the active site. The question as to when the substrate is added to the catalyst mixture, that is, before or after the oxidation of the (pre)catalyst, can result in dramatic changes as elegantly demonstrated by the Long group.^440^ In their case, coordination of l-lactide prior to the oxidation of the ferrocenyl groups in the titanium(IV) catalyst system **[(Ti)120]** (substrate first, oxidant second), sporting the ferrocenyl groups in close proximity to the active site, led to a change in coordination geometry. This, in turn, reversed the activity pattern observed for the opposite addition of reactants (oxidant first, substrate second).^440^

A nickel catalyst **[(Ni)104c]** (Figure 34, bottom; for free **104c**, see Figure 18) reported by the same group serves as an example of complications that can be encountered in RSC.^284^ Even though the appended ferrocenyl groups were, in principle, oxidized by the addition of a suitable oxidant, the necessary addition of methylalumoxane, which generally contains traces of trimethylaluminum, reduced the ferrocenium species again. Thus, the addition of oxidant did not affect the polymerization activity of **[(Ni)104c]**. In contrast, the addition of cobaltocene led to a distinct change in the density of the resulting polyethylene (from low- to medium-density), which the authors attributed to an acenaphthene-centered (marked in orange) reduction, in turn resulting in an increased electron density at the active nickel site.^441^ This example further serves to demonstrate that even purely organic ligands can act as redox noninnocent moieties during catalysis, and corresponding approaches are richly represented in the literature.^442,443^

With what, on a first glance, appear to be only cosmetic changes, Zhao and Chen used a similar α-diimine ligand for binding a methylpalladium(II) fragment (**[(Pd)105]**, Figure 34, bottom; for free **105**, see Figure 18).^15^ Even though the previously discussed catalysts mostly also contained two redox-active ferrocenyl groups in the ligand backbone, we believe Zhao and Chen to have been the first to prove that a stepwise oxidation of these moieties would endow the system with different properties. Before, such multistate switching, albeit being extensively used in terms of molecular machinery,^444−446^ had only once been demonstrated in the catalysis context for a “small” molecule, namely a rotor with organocatalytic activity toward a Michael addition reaction.^447^ Dealing with the potential complications of disproportionation or mixtures with differing oxidation states, Zhao and Chen prepared carbonyl derivatives of their palladium complexes and studied the IR stretching frequency of the C≡O bond, showing only one (albeit very weak) band to be present in each corresponding sample. While, with increasing degree of oxidation, the activity of **[(Pd)105]**^**+/2+/3+**^ in olefin polymerization drastically decreased, the topology, microstructure, and polydispersity of the corresponding products differed significantly between the three distinct activity states.^448^ This can be likely considered as the first full exploitations of the potential of a multiferrocene ligand system in RSC.

Diaconescu and Deng reported on a dimeric yttrium phenoxide complex supported by a ferrocene Schiff base ligand, [(salfen)Y(OPh)]_2_ (salfen = (*N*,*N*′-bis(2,4-di-*tert*-butylphenoxy)-1,1′-ferrocenediimine).^16^ As the complex contains two ferrocenylene groups from its two ligands, it can be oxidized twice in a stepwise fashion to access three oxidation states, all of which are stable under catalytic conditions. The catalytic activity toward cyclic esters decreased upon oxidation while the opposite trend was observed in epoxide polymerization. Employing different oxidation states allowed for the preparation of block copolymers.^16^ A recent review gives an overview of the development of valence-variable metal-based catalysts for redox-induced and redox-controlled switchable polymerization of cyclic esters, cyclic ethers, epoxides, and CO_2_ with a focus on potential applications and challenges for further studies.^449^

**Figure 35:**
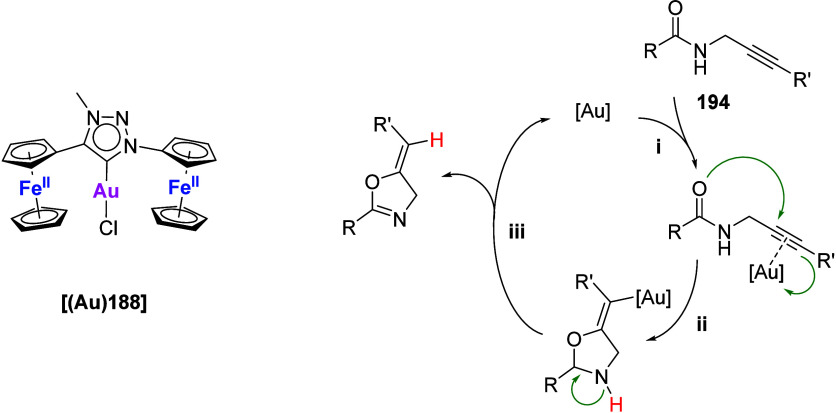
Ferrocene-containing gold(I) (pre)catalyst used in redox-switchable gold catalysis and proposed mechanism for the *5-exo-dig* cyclization of propargylic amides **194** to oxazoline derivatives (right; curved green arrows show the movement of electron pairs).

In 2022, Pérez-Sánchez et al. summarized the use of ferrocenyl gold complexes as efficient catalysts.^450^ In the context of this rapidly expanding field, switchable gold catalysis has only more recently become the subject of extensive research. In a seminal report in 2015, Sarkar and co-workers were able to show that a *C*-ferrocenylated mesoionic carbene bound to gold could be activated toward the *5-exo*-*dig* cycloisomerization of propargylic amide **194** (Figure 35), R = Ph, R′ = H) by oxidation, while the native complex was catalytically silent.^451^ The second entry into the series combined two ferrocenyl groups into the gold complex **[(Au)188]** (Figure 24), yet this compound was not investigated for its potential in a stepwise oxidation approach.^404^ Substituting one or both ferrocenyl groups by the much more electron-withdrawing cobaltocenium cations has led to extremely active catalysts in the conversion of **194**, however at the expense of any degree of *in situ* redox control.^452^**[(Au)188]** and related complexes can, as also shown by the Sarkar group, be used in other gold(I)-catalyzed reactions and the oxidation-induced activation has been investigated with multiple spectroscopic and computational means.^453^

**Figure 36:**
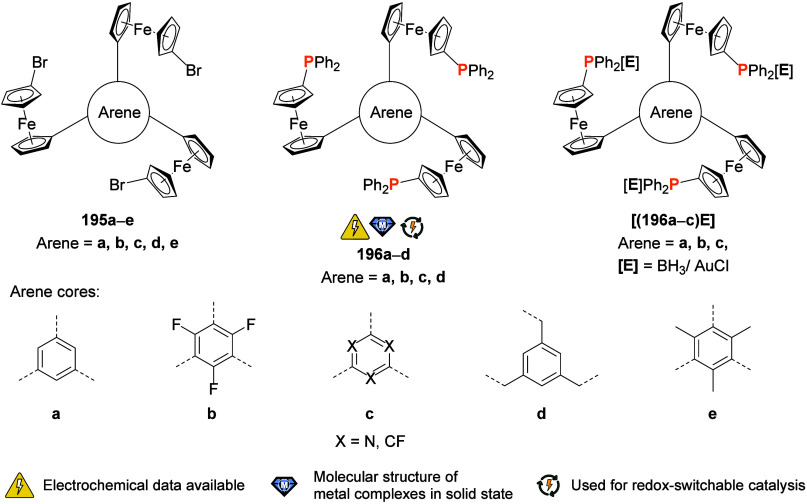
*C*_3_-Symmetric tris(ferrocenyl)arenes prepared by Hey-Hawkins et al.^14,476,477^

Over the past 20 years, *C*_3_-symmetry has increasingly been adopted into ligand design due to enhanced stability and outstanding performances in asymmetric catalysis due to a reduced possible number of transition states.^454,455^ Next to a vast number of nitrogen-containing compounds,^456−459^ several tris-phosphanes have made their prominent entries into the field,^460−464^ often based on the archetypical triphos ligand by Hewertson and Watson.^465^ However, the corresponding *C*_3_-symmetric ligands to feature three ferrocenylene groups, such as **35** (Figure 6) by Butler and co-workers^156^ and macrocycle **44** (Figure 7) by Mizuta and co-workers^182^ remained a curiosity. Motivated by the prominence of *C*_3_-symmetry in modern-day ligand design and the potential to exploit the use of three ferrocenyl groups for RSC, Hey-Hawkins and co-workers built upon their previous works on ferrocenylphosphanes^163,466−473^ and their applications in RSC^110,269,270^ by constructing tris-phosphanes based on a *C*_3_-symmetric tris(ferrocenyl)arene backbone (Figure 36). By incorporating different aromatic cores with varying electron-donating (**e**) or -withdrawing (**b** and **c**) as well as a tris-benzylic arene core (**d**), the electrochemical response as well as more subtle influences on the coordination behavior of the corresponding phosphanes become adjustable. Based on preliminary results regarding the preparation of 1,3,5-tris(1-bromo-1′-ferrocenylene)benzene^474^ and on results for nonsubstituted tris(ferrocenyl)arenes by the groups of Lang^126^ and Heck,^475^ this methodology was transferred to other arenes. The suitability of the resulting (1-bromo-1′-ferrocenylene)arenes **195** for subsequent derivatization toward the target phosphanes was followingly investigated. A new family of tris-phosphanes (**196a**–**d**) incorporating a redox-active, *C*_3_-symmetric tris(ferrocenyl)arene backbone was reported. These ligands are capable of forming both mono- and trinuclear gold(I) complexes; the latter were shown to act as four-state redox-switchable catalysts.^14,476,477^

The four oxidation states relating to the tris(ferrocenyl)arene backbone–non-, mono-, di-, and trioxidized–were identified by cyclic voltammetry using supporting electrolytes with the weakly coordinating [B(3,5-(CF_3_)_2_C_6_H_3_)_4_]^−^ anion (in SEs featuring [PF_6_]^−^, only one oxidation event was observed). Stoichiometric oxidation of **[196a(AuCl)**_**3**_**]** yielded isolable products **[196a(AuCl)_3_]**[TEF]*_n_* ([TEF] = [Al(O(*t*Bu^F^)_4_]^−^, (tetra(perfluoro-*tert*-butoxy)aluminate(−1) or teflonate; *n* = 1, 2, 3). This redox behavior was advantageously used in redox-switchable catalysis *ex* and *in situ*, as shown for the proof-of-principle ring-closing isomerization of *N*-(2-propyn-1-yl)benzamide (**194**) forming the corresponding oxazoline (see Figure 35).^14^ The influence of the central arene core was also demonstrated by electrochemical studies of the corresponding ruthenium(II) and rhodium(I) complexes.^478^ Findings from this study clearly illustrated the need to design systems with well-defined electrochemical responses, as particularly the triazine-based tris-phosphane **196c** showed strong deviations from fully reversible redox chemistry.^478^

Redox-switchable catalysis is of course not limited to “small” molecules and has also been demonstrated for ferrocene-containing dendrimers.^110^ Their high charge density upon oxidation leading to precipitation from nonpolar solvent systems, allowing for their recovery and recycling. More recently, Barner-Kowollik, P. Roesky, and teams have reported a poly(styrene-*co*-chloromethylstyrene) copolymer which they collapsed into a so-called single-chain nanoparticle (SCNP) by linking the chloromethyl groups of the preprepared polymer with 1,1′-dilithioferrocene.^24^ Substituting 1,1′-dilithioferrocene for dilithiated 2-ferrocenyl-1,10-phenanthroline, the resulting SCNPs now featured a binding site for palladium. Preliminary studies of a Pd-catalyzed hydroamination showed these heterometallic SCNPs to be catalytically active, while their potential for RSC remains to be explored.

## 4 Conclusions and Outlook

Ligands incorporating multiple ferrocene units are of interest from different perspectives. The most prominent feature of multiferrocene ligands stems from their ability to, reversibly, undergo redox processes, highlighting their distinctive electrochemical properties. As *n* redox-active groups give rise to *n* + 1 redox states, the selective targeting of a given state with unique properties can be achieved given the right experimental conditions or deliberately chosen unsymmetric ligand design. With respect but not limited to catalysts, such multistate redox-switchable systems thus provide intriguing starting points for fine-tuning and tempo-spatially controlling catalytic activity, influencing and gating the read-out of molecular sensors, or design stimuli-responsive molecular machinery and macroscopic materials. In the wake of the recently discovered more exotic oxidation states and first reports of follow-up chemistry,^479^ harnessing redox chemistry beyond the canonical Fe^II/III^ redox couple makes for exciting (if, at this moment, futuristic) prospects.

Making use of ferrocene’s propensity for planar chirality, multiferrocenyl systems introduce further opportunities for creating a tailored chiral environment for asymmetric catalysis. The steric bulk introduced by ferrocenyl(ene) groups has also been useful in this respect. The combination of these concepts, asymmetric catalysis and redox-switchability, however, remains strikingly underexplored. Given the many available and, sometimes even commercially, successful ligands with two or more ferrocenyl(ene) groups, the potential for manipulating catalytic activities, enabling catalyst recycling or discovering novel reactivities is certainly there.

Before developing new designer ligands, it might be interesting to consult the already existing libraries. Of course, not every reported ligand system will be a good fit–some are too tedious to prepare in quantities necessary for actual applications, others remain, sometimes surprisingly, undercharacterized with respect to crucial electrochemical characteristics: only in 2007 did the Nataro group report an in-depth investigation of the electrochemical behavior of Josiphos-type ligands first reported in 1996.^480^ As shown especially for ferrocenylphosphanes, complex electrochemistry can seem prohibitive for the free ligand, but turn out unproblematic once the donor atoms are engaged in coordinative bonds, or *vice versa*. Knowledge about the redox windows of the ferrocenyl(ene) groups, the catalytically active metal centers in isolation, and their combination, is thus crucial, and whether to install ferrocene groups in close proximity to the donor atoms or as more distant termini will depend on the targeted application. In addition, many ligands covered in this review are rotationally symmetric as *C*_2_-symmetry in particular has proven a useful concept in asymmetric catalysis–but this very symmetry will make the selective address of one over the other ferrocenyl(ene) group impossible, complicating attempts to toggle between more than just fully reduced and oxidized state.

With this review, we have attempted to cover various multiferrocene ligand classes including P, N, O, S, Se, and C donors as well as selected examples of other donor systems. Necessarily, our survey of the vast body of literature will have overlooked some more or less relevant entries into these groups, and we cannot claim this review to be comprehensive. However, we hope to provide a guide for the ongoing journey of the interested community and look forward to seeing the exciting applications that multiferrocene ligands can enable.
